# Carbodiimide‐Driven Dimerization and Self‐Assembly of Artificial, Ribose‐Based Amphiphiles

**DOI:** 10.1002/chem.202104116

**Published:** 2022-02-08

**Authors:** Jing Sun, Julian Vogel, Lisa Chen, A. Lennart Schleper, Tim Bergner, Alexander J. C. Kuehne, Max von Delius

**Affiliations:** ^1^ Institute of Organic Chemistry Ulm University Albert-Einstein-Allee 11 89081 Ulm Germany; ^2^ Institute of Macromolecular and Organic Chemistry Ulm University Albert-Einstein-Allee 11 89081 Ulm Germany; ^3^ Central Facility for Electron Microscopy Ulm University Albert-Einstein-Allee 11 89081 Ulm Germany; ^4^ DWI – Leibniz-Institute for Interactive Materials Forckenbeckstraße 50 52074 Aachen Germany

**Keywords:** amphiphile, dimerization, hydrolysis, pyrophosphate, ribonucleotide, self-assembly

## Abstract

The aqueous self‐assembly of amphiphiles into aggregates such as micelles and vesicles has been widely investigated over the past decades with applications ranging from materials science to drug delivery. The combination of characteristic properties of nucleic acids and amphiphiles is of substantial interest to mimic biological self‐organization and compartmentalization. Herein, we present ribose‐ and ribonucleotide‐based amphiphiles and investigate their self‐assembly as well as their fundamental reactivity. We found that various types of aggregates are formed, ranging in size from nanometers to micrometers and all amphiphiles exhibit aggregation‐induced emission (AIE) in solution as well as in the solid state. We also observed that the addition of 1‐ethyl‐3‐(3‐dimethylaminopropyl)carbodiimide (EDC) leads to rapid and selective dimerization of the amphiphiles into pyrophosphates, which decreases the critical aggregation concentration (CAC) by a factor of 25 when compared to the monomers. Since the propensity for amphiphile dimerization is correlated with their tendency to self‐assemble, our results may be relevant for the formation of rudimentary compartments under prebiotic conditions.

## Graphical abstract

**Two types of artificial ribose‐based amphiphiles** have been prepared and self‐assembled into various supramolecular structures, exhibiting aggregation‐induced emission. The molecularly dissolved amphiphiles can be converted selectively into dimeric pyrophosphates in the presence of carbodiimide EDC, giving rise to helical fibers with lengths at the micrometer scale.

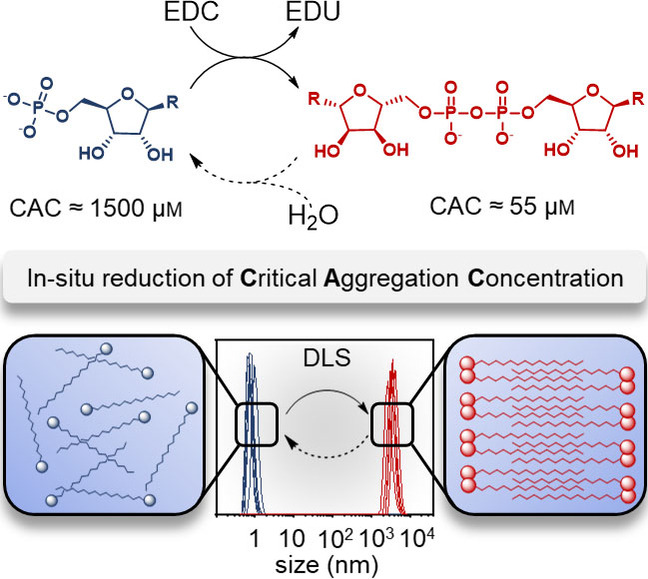

## Introduction

Molecular self‐assembly is the spontaneous equilibration of molecules into thermodynamically stable supramolecular structures.[^1^, ^2^, ^3^, ^4^, ^5^, ^6^, ^7^, ^8^, ^9^, ^10^] In water, this behavior is ubiquitous and underpins the formation of a wide variety of compartments and complex biological architectures. Biomolecule‐based amphiphiles and their aggregates[^11^, ^12^, ^13^, ^14^, ^15^, ^16^] are currently of particular interest, because they have been used to generate self‐healing materials,^[17]^ biosensors,^[18]^ and novel therapeutics.[^12^, ^19^] Much of the biological function of DNA and RNA is based on the capability of the fundamental building blocks to form highly specific hydrogen bonds.[^20^, ^21^] Thus, the structural amalgamation of nucleic acids with macrocyclic[^22^, ^23^] or amphiphilic properties has the potential to generate hierarchical self‐assemblies with unexpected structures and properties.

Although nucleotide/ribonucleotide‐based amphiphiles have been reported previously,[^24^, ^25^, ^26^, ^27^, ^28^, ^29^] the generation of a dissipative reaction network[^30^, ^31^] based on the aggregation of these building blocks is still elusive. Inspired by recent work on dissipative assemblies of carboxylic anhydrides[^32^, ^33^] and ATP‐metal complexes,[^34^, ^35^, ^36^, ^37^] we hypothesized that artificial ribose‐based amphiphiles could exhibit similar behavior, when driven to dimerize into phosphodiesters or pyrophosphates by a chemical fuel and then left to hydrolyze after consumption of the fuel. While the forward reaction has been studied extensively by Szostak and Richert from the perspective of prebiotically plausible oligonucleotide formation and lately even translation,[^38^, ^39^, ^40^, ^41^, ^42^, ^43^, ^44^, ^45^, ^46^, ^47^] the hydrolysis of phosphodiesters and pyrophosphates has received somewhat less attention.[^48^, ^49^]

In this context, we report the design, synthesis, and self‐assembly, of ribose‐ and ribonucleotide‐based amphiphiles, composed of the polar ribomonophosphate or ribonucleotide head group and a nonpolar alkyl tail group. Nine different compounds with unique properties were successfully synthesized and characterized. The amphiphiles self‐assemble into various superstructures ranging in size from nanometers to micrometers, which can be tuned by varying the alkyl chain length. We demonstrated that the chemical “fuel” 1‐ethyl‐3‐(3‐dimethylaminopropyl)carbodiimide (EDC) drove the dimerization of these amphiphiles selectively into pyrophosphates that were up to 25 times more prone to aggregate than the corresponding monomers. We further investigated a wide range of conditions for the hydrolysis of the pyrophosphate dimers and found that, while possible in principle, the successful conditions are too harsh to allow closing the dissipative pyrophosphate‐based reaction cycle, at least for this first generation of amphiphiles.

## Results and Discussion

### Synthesis, characterization and aggregation of monomeric amphiphiles

**Scheme 1:**
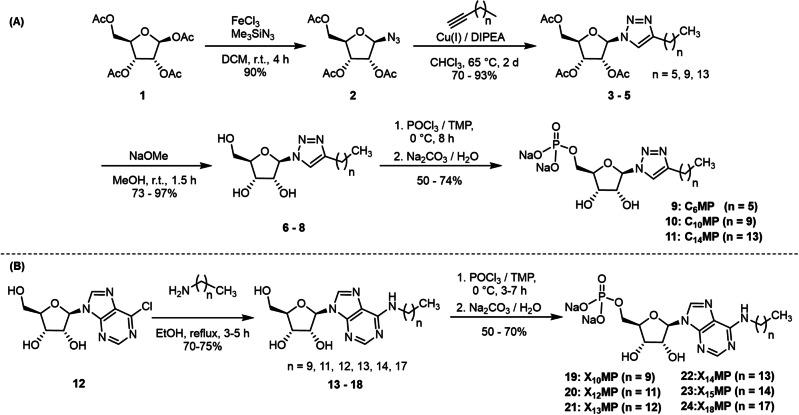
Synthetic routes towards two types of amphiphiles. A) Triazole‐based ribomonophosphates (**C_n_MP**) and B) ribonucleotide amphiphiles (**X_n_MP**), respectively.

To explore the self‐assembly of ribonucleotide‐based amphiphiles, we synthesized two different types of amphiphiles, including triazole‐based ribomonophosphates, herein named **C_n_MP**, and derivatives of natural ribonucleotides, named **X_n_MP** (n denotes the length of the alkyl chain). These two types of amphiphiles were designed in order to explore the influence of π–π interactions and hydrogen bonding on their self‐assembly, while remaining relatively close to the structure of natural nucleotides. As shown in Scheme 1, the **C_n_MPs** were synthesized from commercially available 1,2,3,5‐tetra‐O‐acetyl‐*β*‐D‐ribofuranose (**1**) by acetate/azide substitution (**2**),^[50]^ followed by copper‐catalyzed 1,3‐dipolar Huisgen‐cycloaddition (**3**–**5**),^[51]^ deacylation (**6**–**8**), and subsequent phosphorylation (**9**–**11**) in 50–74 % yields. Similarly, the **X_n_MPs** were synthesized in two steps by nucleophilic aromatic substitution of 6‐chloropurine riboside (**12**) and phosphorylation (**19**–**24**) in 50–70 % yields.^[52]^ All amphiphiles were characterized by nuclear magnetic resonance spectroscopy (^1^H NMR, ^13^C NMR, and ^31^P NMR) and high‐resolution mass spectrometry (HR‐MS) (Figures S19‐S80).

**Figure 1:**
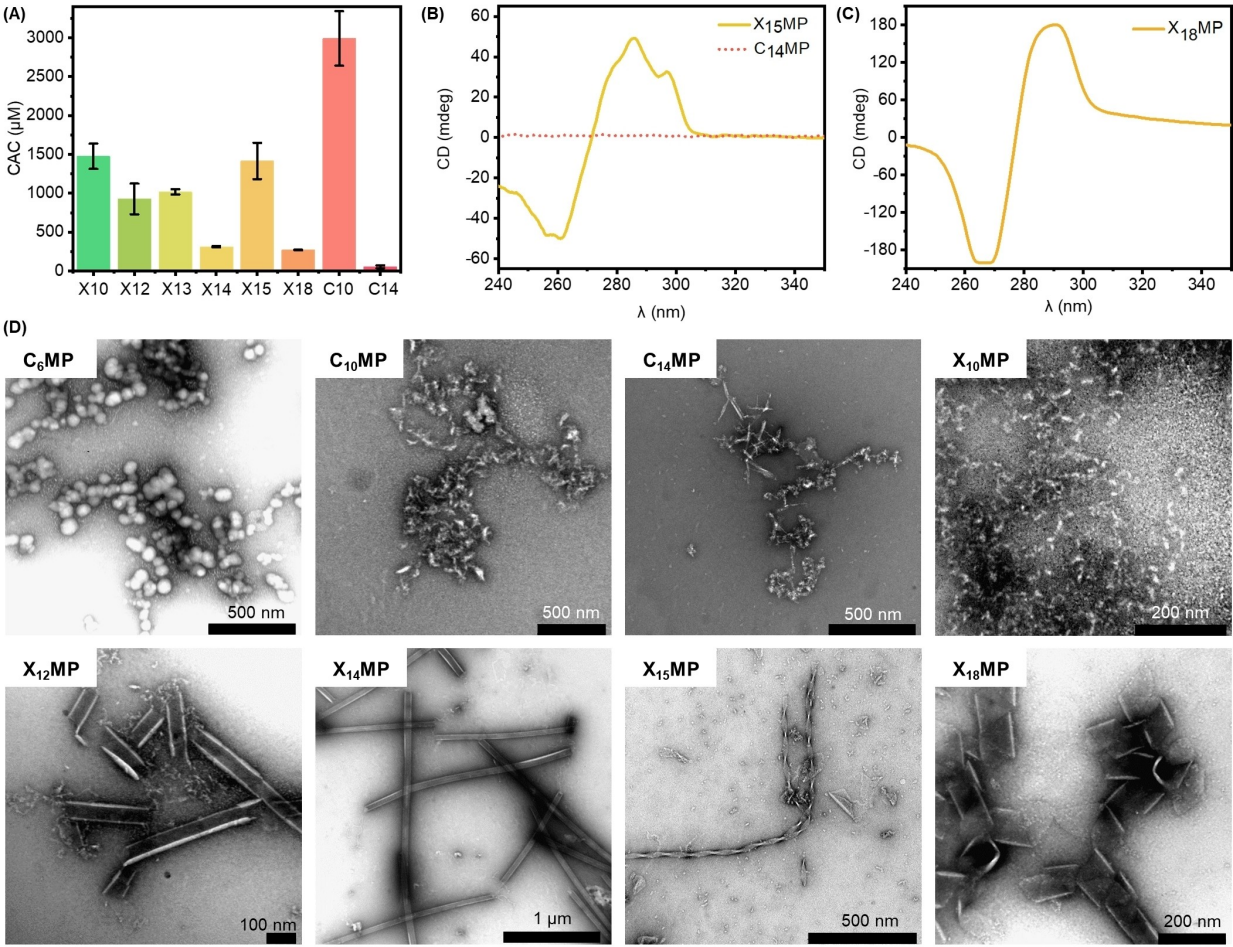
Characterization of amphiphiles. A) The critical aggregation concentration (CAC) of ribose‐based amphiphiles was determined using 1,6‐diphenyl‐1,3,5‐hexatriene (DPH) as a fluorescent dye. Three parallel experiments were performed to determine the average CAC values. B–C) Circular dichroism (CD) spectrum of representative amphiphiles in 5 mm HEPES buffer (pH 7.0) at 25 °C, including (B) **X_15_MP** (2 mm) and **C_14_MP** (0.16 mm) and (C) **X_18_MP** (0.8 mm), respectively. D) Transmission electron microscopy (TEM) images above CAC of spherical aggregates of **C_6_MP** (18 mm), ill‐defined aggregates of **C_10_MP** (6 mm), **C_14_MP** (0.16 mm), **X_10_MP** (2.0 mm), semi‐tubes of **X_12_MP** (1.3 mm), hollow tubes of **X_14_MP** (2.2 mm), helical fibers of **X_15_MP** (5.0 mm), and helical ribbons of **X_18_MP** (0.5 mm) formed by the self‐assembly of amphiphiles in 5 mm HEPES buffer (pH 7.0). All samples were negatively stained with UO_2_(OAc)_2_.

The critical aggregation concentration (CAC) of ribonucleotide amphiphiles was first investigated using the nonpolar fluorescent dye 1,6‐diphenyl‐1,3,5‐hexatriene (DPH).^[36]^ This dye is almost non‐emissive in an aqueous solution due to aggregation‐caused quenching. However, once the CAC of the amphiphiles is reached, the dye will be encapsulated in the nonpolar compartment formed by the hydrophobic tails, leading to an increase in the photoluminescence intensity. To determine the CAC of each amphiphile, we carried out photoluminescence titrations of DPH (10 μm, λ_ex_=355 nm, λ_em_=428 nm) in 4‐(2‐hydroxyethyl)‐1‐piperazineethanesulfonic acid (HEPES) buffer (pH 7.0) in the presence of increasing concentrations of amphiphiles (Figures 1A and S1). As expected, it was found that the CAC decreases as the aliphatic chain length increases. However, the CAC of **X_15_MP** was determined to be around 1.5±0.2 mm, which was nearly as high as that of **X_10_MP**. This unusual behavior might be related to an odd‐even effect, which is known to occur in alkylated light‐emitting dyes.[^53^, ^54^] In agreement with this reasoning, the CAC of **X_13_MP** represents an outlier within the decreasing trend from **X_10_MP** to **X_14_MP** (Figure 1A). The CAC of **C_6_MP** could not be investigated by this method, as its aliphatic chain is presumably too short to incorporate DPH into aggregates. It was estimated, however, by making use of the AIE properties (see below). Moreover, dynamic light scattering (DLS) was used to determine the average size of aggregates in solution (Figure S2). Analysis of the data for the different amphiphile solutions (in concentrations about two times above CAC) yielded hydrodynamic diameters around 75–250 nm, indicating the formation of aggregates.

We used a variety of analytical techniques to explore the structures of these aggregates. The formation of spherical aggregates with diameters of 75–100 nm for **C_6_MP** was confirmed by transmission electron microscopy (TEM) (Figures 1D and S3). The longer alkyl chain lengths in **C_n_MPs** lead to ill‐defined aggregates (Figures 1D and S3). Similarly, **X_10_MP** and **X_13_MP** also show random aggregation above their CAC (Figures 1D and S3). However, a completely different morphology was observed for the longer **X_n_MPs** in the HEPES buffer (Figure 1D), which might be due to stronger hydrophobic interactions induced by the longer alkyl chains. When the chain length was between 12 and 14, the amphiphiles formed tubular structures, including a semi‐tube (**X_12_MP**) and a hollow tube (**X_14_MP**). This hollow tube structure of **X_14_MP** was further confirmed by scanning electron microscopy (SEM) (Figure S4). Interestingly, helical aggregates were observed consistently for chain lengths exceeding 14, such as helical fibers (**X_15_MP**) and helical ribbons (**X_18_MP**) (Figure 1D). The helical characteristics were confirmed by circular dichroism (CD) spectroscopy (Figures 1B and 1C). The results corroborate helical aggregation as confirmed by the Cotton effect at wavelengths between 260–300 nm. It should be noted that the pristine adenosine monophosphate with a chiral environment of ribose, **X_15_MP** below its CAC (Figure S5) and **C_14_MP** above its CAC (Figure 1B) did not show any significant optical activity in the CD spectrum due to the lack of aggregation. The fact that **X_14_MP** forms well‐defined structures, while **C_14_MP** does not, suggests that hydrogen bonding or π–π stacking are at least equally important as the hydrophobic effect for determining the aggregates’ structure.[^55^, ^56^] Presumably, either the larger π‐surface or the hydrogen bond acceptor atoms in the purine moiety lead to the self‐assembly of sheets that commonly exhibit a helical pitch.^[57]^

### Unexpected observation of aggregation‐induced emission (AIE)

**Figure 2:**
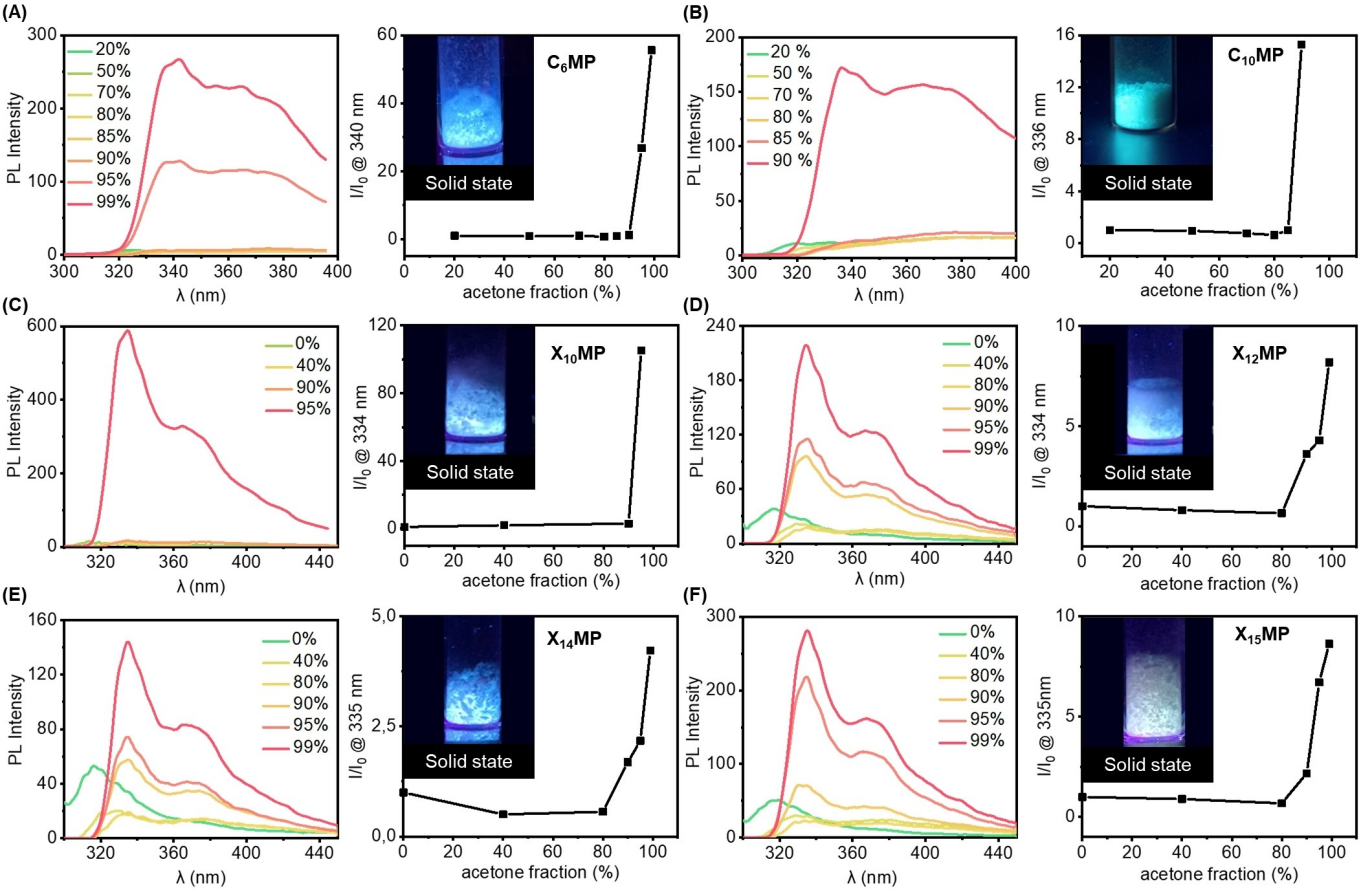
Unexpected aggregation‐induced emission (AIE) behavior of ribose‐based amphiphiles. Acetone was chosen as an anti‐solvent. The PL spectra of amphiphiles and the plot of maximum emission wavelength in H_2_O‐acetone mixtures with different acetone fractions (*f*_a_), including (A) **C_6_MP**, (B) **C_10_MP**, (C) **X_10_MP**, (D) **X_12_MP**, (E) **X_14_MP**, (F) **X_15_MP**. Data points in the I/I_0_ plots are connected to guide the eye, I_0_ and I represent the PL intensity in pure water and H_2_O/acetone at the maximum emission wavelength, respectively. Excitation wavelength: 280 nm. Insets show photos of all amphiphiles in solid state under 365 nm UV lamp irradiation.

It is well documented that natural nucleotides or ribonucleotides are typically not photoluminescent.^[58]^ In stark contrast, the ribose‐based amphiphiles reported herein exhibit typical AIE properties, as evidenced by their respective photoluminescence (PL) spectra measured in a solvent mixture (H_2_O/acetone) with varying acetone fractions, which functions as anti‐solvent. As shown in Figure 2, the solution of these amphiphiles exhibited no or relatively weak emission when the ratio of the anti‐solvent *f*_a_ was below 90 %. Interestingly, the emission increased significantly when *f*_a_ was increased to 90 %‐95 % to induce aggregation. TEM confirmed the self‐assembly in high acetone fractions (Figure S6). These aggregated structures were less well‐defined than in pure HEPES buffer above CAC. Nevertheless, random aggregates and helical fibers were observed when *f*_a_ was above 95 %. In addition, all these amphiphiles emitted light in the blue spectrum, when irradiated in the solid state with ultraviolet (UV) light (365 nm). Of note, **C_10_MP** emitted greenish light under UV irradiation, which is different from all other amphiphiles that emitted blue light (Figure 2B). We further determined the PL quantum yield (QY) of representative amphiphiles in the solid state. Accordingly, we observed PL QYs of 13 % for **C_10_MP** and 14 % for **C_6_MP**, which is far below state‐of‐the‐art AIE dyes,[^59^, ^60^] but interesting for compounds lacking typical AIE motifs (Figure S7).

Beyond these studies in solid state and with varying solvent compositions, we found that the PL intensities of amphiphiles were continuously enhanced with increasing concentration in aqueous solutions (Figure S8). When the concentration is above its CAC, the photoluminescence intensities of amphiphiles are almost linearly related to their concentrations. These results further corroborate that the alkyl chain functionalized ribonucleotide amphiphiles have AIE properties. We suggest that this unexpected AIE behavior is explained by “clusteroluminescence”,^[61]^ specifically due to intermolecular through‐space conjugation of the π‐systems and/or electron‐rich heteroatoms. In related aromatic^[62]^ or carbohydrate[^63^, ^64^, ^65^] systems, this type of through‐space conjugation was identified to open up radiative decay pathways. In turn, we were able to determine the CAC of our ribonucleotides by determining the change in AIE during concentration‐dependent luminescence measurements in HEPES buffer and found these values to be in good agreement with the ones determined before by the addition of DPH dye (Figure S8).

### Carbodiimide‐driven formation of pyrophosphate‐linked amphiphile dimers

**Figure 3:**
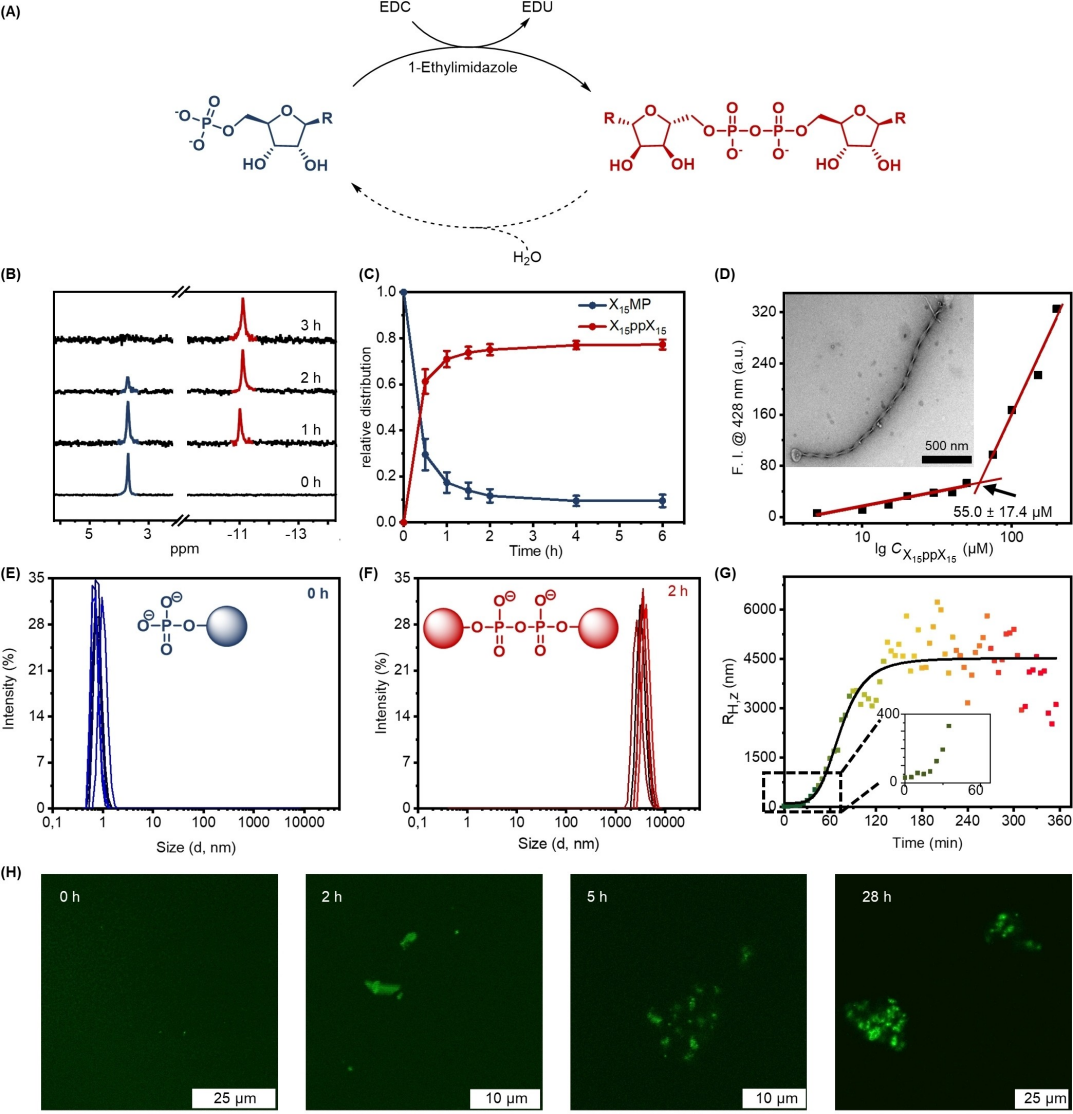
A) The EDC‐triggered dimerization of **X_15_MP**, yielding the formation of pyrophosphate **X_15_ppX_15_**. B) ^31^P NMR of the dimerization reaction over time. C) The kinetics of the dimerization reaction over 6 h by HPLC. 80 % of the monomer is converted to the dimer within 6 h. Three parallel experiments were performed for the HPLC kinetics study. D) CAC determination of isolated **X_15_ppX_15_**. The insert shows the assembled structure of isolated **X_15_ppX_15_** (100 μm in 0.5 mm HEPES buffer) confirmed by TEM. E–H) Characterization of the dimerization of 500 μm **X_15_MP** in condensation buffer, driven by 50 eq. of EDC. Size distribution (E) before and (F) after dimerization monitored by DLS (data curves represent ten replicate experiments). G) Sigmoidal kinetics of aggregation as observed by DLS. H) Confocal images over time, showing the formation of aggregates. Samples were prepared using 2.5 μm C153.

We proceeded to explore the reaction of the monomeric phosphates with the water‐soluble carbodiimide EDC and nucleophilic catalyst 1‐ethylimidazole (EtIm) (Figure 3A).[^39^, ^66^] Initially, the different amphiphiles were compared and the amount of EDC was optimized to achieve EDC‐driven dimerization. It was found that 50–100 equivalents of EDC led to rapid dimer formation, and **X_15_MP** reacted especially efficiently when compared to other amphiphiles (Figure S9). In general, the **X_n_MPs** were more prone to dimerization than the **C_n_MPs**, suggesting that there is a correlation between propensity for aggregation and dimerization. Exemplarily, **X_15_MP** (0.5 mm, below CAC) was dimerized upon addition of EDC (50 eq.) in a 0.5 mm 3‐(N‐morpholino)propanesulfonic acid (MOPS) buffer, containing 1‐ethylimidazole (0.15 mm) and MgCl_2_ (0.08 mm) (pH 7.5). These reaction conditions were derived from Richert and co‐workers and are referred to as “condensation buffer.”^[46]^ As shown in Figure 3B, the ^31^P NMR signal (∼3.8 ppm) of **X_15_MP** gradually decreased and vanished after 3 h. Meanwhile, a new signal around ‐11.0 ppm appeared and accumulated, indicating the formation of pyrophosphate **X_15_ppX_15_**, based on the characteristic chemical shift.[^47^, ^67^] While non‐substituted adenosine monophosphates also form some phosphodiesters,[^47^, ^67^] we did not observe this for **X_15_MP**. This selective transformation behavior might be attributed to steric hindrance induced by the long alkyl chains.

Subsequently, the reaction kinetics were studied by high‐performance liquid chromatography (HPLC) under different temperatures, including 25 °C, 10 °C, and 0 °C (Figures 3C, S10A and S11A–C). When the dimerization was performed at room temperature (25 °C), HPLC analysis indicated that 80 % of **X_15_MP** was consumed and converted to pyrophosphate **X_15_ppX_15_** within 6 h. The second‐order rate constant was determined to be 2.1×10^−6^ M^−1^ s^−1^ at a concentration of **X_15_MP** of 0.5 mm (below CAC) (Figure S10B). While the polymerization of (activated) ribonucleotides was reported to be faster at temperatures in^[68]^ or near^[66]^ the eutectic ice phase, we observed that the reaction rate was slowed down when decreasing the temperature. Decreasing the pH from 7.5 to 6.0 did not have any significant effect on the reaction kinetics (Figure S11). In addition, the kinetics of the hydrolysis of EDC during phosphate activation was investigated by ^1^H NMR spectroscopy (Figure S12A). We found that EDC is consumed faster in the presence of monophosphates than in the pure buffer (Figure S12B) and that the rate of consumption is comparable for different monophosphates (Figure S12C).

To gain additional insights into EDC‐triggered dimerization of **X_15_MP**, we successfully isolated the pyrophosphate by semi‐preparative HPLC (Figures S81–84). Notably, the CAC value of **X_15_ppX_15_** was determined to be 55.0±17.4 μm, which is over 25 times lower than that of **X_15_MP** (Figure 3D). The ultra‐low CAC value of **X_15_ppX_15_** leads to the rapid formation of large aggregates during the reaction, yielding helical fibers (Figures 3D and S13).

We also monitored the EDC‐driven dimerization of **X_15_MP** by DLS to find out at which point of time aggregation starts to occur. In stark contrast to the non‐aggregating starting material **X_15_MP** (0.5 mm, below CAC), the formation of **X_15_ppX_15_** led to a significant increase in the hydrodynamic diameter (∼1 μm) in aqueous solution, indicating the formation of large aggregates (Figures 3E–F). The formation of helical aggregates in solution upon dimerization was further confirmed by cryogenic TEM (cryo‐TEM) (Figure S14).

The aggregation kinetics were monitored by DLS (Figure 3G), and the data shows a rapid increase in average hydrodynamic diameter ca. 30 minutes after addition of EDC (25 mm) to a solution of X_15_MP (0.5 mm) and then entering a plateau after ca. 120 minutes. Such a sigmoidal kinetic curve typically suggests a cooperative effect, in this case between self‐assembly and pyrophosphate formation. After adding EDC, pyrophosphates form slowly and start to aggregate as soon as their CAC is reached. At this point, incorporation of **X_15_MP** into the hydrophobic aggregates accelerates pyrophosphate formation, presumably due to increased effective molarity.^[69]^ In the absence of EDC, all control experiments (i: **X_15_MP**+1‐ethyl‐3‐(3‐dimethylaminopropyl)urea (EDU) in condensation buffer; ii: EDU in condensation buffer; iii: blank, pure condensation buffer) showed that the average hydrodynamic diameters remained constant over time (Figure S15), indicating that the formation of pyrophosphate is indeed responsible for the observed aggregation. Moreover, confocal laser scanning microscopy (CLSM) studies directly visualized large aggregate formation (Figure 3H). Accumulation of the coumarin 153 (C153) fluorophore in the aggregates allowed these to be detected after adding EDC. Similarly, the control experiments (i: **X_15_MP**+EDU+C153 in condensation buffer; ii: EDU+C153 in condensation buffer; iii: blank, C153 in condensation buffer) did not show any distinguishable fluorescent structures from CLSM images over time, suggesting that the aggregates can be formed only in the presence of EDC (Figure S16).

### Systematic study of pyrophosphate hydrolysis

**Figure 4:**
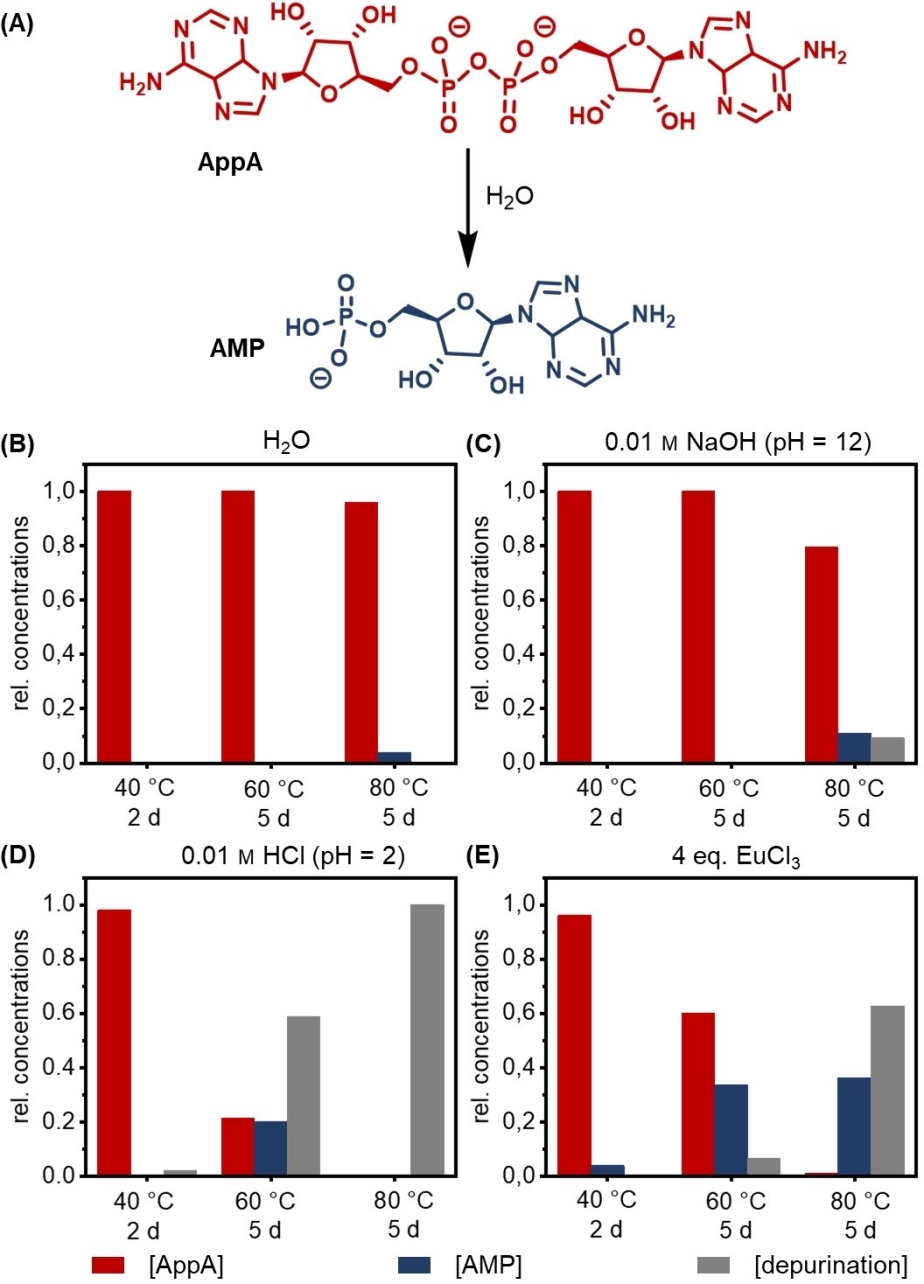
Investigation of pyrophosphate hydrolysis under different conditions using **AppA** as a model. A) Reaction scheme. B–E) 1 mm solutions of **AppA** were stirred at 40 °C for two days before the temperature was increased first to 60 °C and then to 80 °C for another five days each. Hydrolysis conditions were (B) H_2_O, (C) 0.01 m NaOH (pH=12), (D) 0.01 m HCl (pH=2), (E) 4 eq. EuCl_3_ (pH≈6). Relative concentrations were obtained by HPLC.

After establishing the EDC‐driven dimerization process, we investigated the hydrolysis of pyrophosphates, because suitable hydrolysis conditions could render the dimer‐based aggregates described previously transient. We chose to study the hydrolysis of commercially available non‐aggregating adenosine pyrophosphate (**AppA**) as a model reaction (Figure 4A) and monitored the process by HPLC‐MS. The relative concentrations were obtained from the diode array detector signal under the reasonable assumption that the molar absorptivity of the pyrophosphate **AppA** is twice that of adenosine monophosphate (**AMP**). A 1 mm solution of **AppA** was subjected to various conditions, including pure water, 0.01 m HCl solution (pH 2.0), 0.01 m NaOH solution (pH 12.0), and 4 mm EuCl_3_ solution (pH≈6) (Figures 4 and S17).

Because pyrophosphates are known to be relatively stable in aqueous solution,[^29^, ^39^, ^70^] temperatures above room temperature were applied to increase the rate of hydrolysis. It was found that under neutral conditions, **AppA** is relatively inert and only starts to hydrolyze very slowly at 80 °C (Figure 4B). When incubated at 100 °C for one week, some **AppA** was converted to **AMP**, but a significant part decomposed (Figure S17A). We suspect that depurination by cleavage of the hemiaminal ether is the cause of decomposition.[^71^, ^72^, ^73^, ^74^] Similarly, no hydrolysis occurred in the sample treated with 0.01 m NaOH solution at 40–60 °C (Figure 4C). In fact, **AppA** was mostly stable under strong basic conditions even at 100 °C for one week (Figure S17B). By contrast, depurination was the dominant reaction at 60–80 °C under acidic (0.01 m HCl) reaction conditions (Figure 4D).

As lanthanide salts have been reported to catalyze the hydrolysis of phosphodiester bonds in RNA,[^75^, ^76^] EuCl_3_ was tested and yielded more encouraging results (Figure 4E). At 40 °C and 60 °C, the hydrolysis was faster than depurination but still too slow to establish a dissipative reaction network, because even after five days at 60 °C there was still around 60 % of the pyrophosphate left. When increasing the temperature to 80 °C, competing depurination became dominant. Applying these conditions to the alkyl chain substituted **AppA** (namely **X_15_ppX_15_**) led to a similar extent of depurination (Figure S18). Overall, the pyrophosphate bond in **AppA** derivatives seems too stable to be hydrolyzed under conditions where no depurination occurs, which unfortunately makes dissipative systems based on a labile pyrophosphate bond in RNA derivatives inaccessible.

As a last resort, enzymatic hydrolysis of **X_15_ppX_15_** was considered. It is known that pyrophosphatase can break the diphosphate bond efficiently under mild conditions.[^49^, ^77^] A 365 μm solution of **X_15_ppX_15_** was incubated at 37 °C in a 0.5 mm HEPES buffer (pH=7.5) with the enzyme for 3 days. To our surprise, **X_15_ppX_15_** was not hydrolyzed with this specific enzyme (Table S1). This behavior might be due to aggregation or the long alkyl chains that prevent inclusion into the enzyme pocket.

## Conclusion

In conclusion, we report the design, synthesis, self‐assembly, and dimerization of novel ribonucleotide‐based amphiphiles. A family of negatively charged amphiphiles with different chain lengths were synthesized, including natural ribonucleotide‐based amphiphiles and triazole‐based ribonucleotide analogues. The resulting amphiphiles self‐assemble into different superstructures in aqueous solutions ranging in size from nanometers to micrometers, including micelles, tubes, helical fibers, and ribbons. To our surprise, we observed that these amphiphiles, when irradiated with UV light, exhibit aggregation‐induced emission. We were able to demonstrate that these monophosphate amphiphiles can be dimerized to form pyrophosphates in the presence of EDC. When carried out below the CAC of the monomer, this type of experiment therefore leads to the rapid, in situ formation of large aggregates at the micrometer scale. Unfortunately, our efforts to complete the dissipative reaction cycle by controlling the kinetics of pyrophosphate hydrolysis were unsuccessful, despite promising results using EuCl_3_. Nevertheless, this work contains valuable lessons for those who seek to address a crucial weakness of the RNA world theory by combining fuel‐driven oligomerization with compartmentalization.

## Experimental Section

**Dimerization of X_15_MP and monitoring by HPLC and DLS**: 0.30 mg (0.5 μmol, 500 μm) **X_15_MP** were dissolved in 1 mL 3‐(N‐morpholino)propanesulfonic acid (MOPS) buffer (0.5 mm MOPS, 0.15 mm 1‐ethylimidazole, 0.08 mm MgCl_2_, pH=7.50, filtered through a 0.45 μm syringe filter) and 4.78 mg (25 μmol, 25 mm, 50 eq.) EDC*HCl were added. The progress of dimerization and aggregation was monitored by HPLC (254 nm) and DLS (see below). For HPLC, an Ascentis C8 (100×4.6 mm, 3 μm) analytical column and a mobile phase comprising MeCN and 50 mm NH_4_HCO_3_ buffer (pH=8.00) (gradient condition: 6 : 4 (0–3 minutes), 6 : 4–7 : 3 (3–4 minutes), 7 : 3 (4–15 minutes) with a flow rate of 1.0 mL min^−1^ at 60 °C was used.

**Determination of critical aggregation concentration (CAC)**: A stock solution of the amphiphiles in buffer solution and a dilution series with the same buffer were prepared. A 1 mm solution of diphenylhexatriene (DPH) in THF was added to each sample to give a final concentration of 10 μm. The samples were vortexed and equilibrated overnight in the dark. Photoluminescence spectroscopy of each sample was recorded at *λ*_ex_=355 nm on a PerkinElmer FL 6500 spectrophotometer. The emission intensity at *λ*_em_=428 nm was monitored and plotted against the logarithmic concentration. A linear regression (y_n_=m_n_x+a_n_) through the data points before and after the sudden increase in intensity were calculated, respectively. The x‐value of the intersection of these two lines was used to calculate the CAC.

**Aggregation induced emission (AIE)**: To prove that the amphiphiles exhibit an aggregation‐induced emission effect, we performed an anti‐solvent experiment by the addition of acetone (anti‐solvent) to an aqueous solution of amphiphiles which's total concentration was kept constant and below the CAC and the emission intensity with *λ*_ex_=280 nm and *λ*_em_=334–340 nm of the amphiphiles was measured. Photoluminescence spectra in water/acetone mixtures were recorded on a PerkinElmer FL 6500 spectrophotometer. Solid state PL QYs were determined using an integrating sphere.

**Transmission electron microscopy (TEM)**: Amphiphiles in concentrations about two times above CAC were dissolved in either H_2_O or 5 mm HEPES buffer and deposited on copper grids. Negative staining with uranyl acetate was performed. Control experiments with pure water and pure HEPES buffer did not show any assembled structures. TEM measurements were performed on a Zeiss EM10 microscope with an acceleration voltage of 120 kV.

**Cryogenic transmission electron microscopy (Cryo‐TEM)**: **X_15_MP** (below CAC) or **X_15_MP** assembly in condensation buffer (after 2 h) were adhered to a freshly glow‐discharged holey carbon grid. After blotting, the grids were vitrified in liquid ethane by a Vitrobot FP 5350/60 (FEI, Eindhoven, Netherlands). The cryo‐TEM grids were analyzed in a JEM‐2100F (JEOL) at 200 kV and a temperature of −150 °C using a Gatan cryo‐holder. Contrast enhancement was performed with the software ImageJ and Adobe Photoshop.

**Scanning electron microscopy (SEM)**: 5 mm X_14_MP and X_18_MP in 5 mm HEPES buffer were deposited on a silicon wafer and sputtered with a layer of gold after drying in vacuum before measurement. The morphology of both samples was examined by SEM (ZEISS EVO MA microscope).

**Circular dichroism (CD) spectroscopy**: CD spectra of 5 mm **X_15_MP** and **X_18_MP** in 5 mm HEPES buffer were measured at 25 °C in a 1 mm quartz cuvette on Jasco J‐810 CD spectrometer equipped with Julabo F12 temperature controller.

**Dynamic light scattering (DLS)**: The size of the amphiphiles and the assemblies were measured by DLS on a Nano‐Zetasizer (Malvern Instruments) at 25 °C with a scattering angle of 173° at *λ*=633 nm.

**Confocal laser scanning microscopy (CLSM)**: Confocal microscopy images of all samples were recorded with a TCS SP8 confocal microscope using the Leica Application Suite X (LASX) with the LIGHTNING wizard. A droplet of the mixture was placed between two glass slides for confocal measurements. The fluorescent dye (C153) was excited at a wavelength of either 405 nm or 514 nm. Image analysis was performed with the software ImageJ and Adobe Photoshop.

## Conflict of interest

The authors declare no conflict of interest.

1

## Supporting information

As a service to our authors and readers, this journal provides supporting information supplied by the authors. Such materials are peer reviewed and may be re‐organized for online delivery, but are not copy‐edited or typeset. Technical support issues arising from supporting information (other than missing files) should be addressed to the authors.

Supporting Information

## Data Availability

The data that support the findings of this study are available from the corresponding author upon reasonable request.
