# Supplementary material for: Carbodiimide‐Driven Dimerization and Self‐Assembly of Artificial, Ribose‐Based Amphiphiles
Source: Chemistry. 2022 Feb 8;28(13):e202104116. doi: 10.1002/chem.202104116 (PMC9303926; doi:10.1002/chem.202104116)
Supplement: Supplementary file 1 — Supporting Information [file CHEM-28-0-s001.pdf]

Chemistry–A European Journal

Supporting Information

Carbodiimide-Driven Dimerization and Self-Assembly of Artificial, Ribose-Based Amphiphiles

Jing Sun, Julian Vogel, Lisa Chen, A. Lennart Schleper, Tim Bergner, Alexander J. C. Kuehne, and Max von Delius*

Table of Contents

Experimental section. S3

General and Materials S3

Synthesis S3

2,3,5-tri-*O*-acetyl-*β*-D-ribofuranosyl azide 2 .... S3

Deprotection to 4-Alkyl-1-(*β*-D-arabinofuranosyl)-1,2,3-triazoles 6 – 8 .... S5

Phosphorylation to 4-Alkyl-1-(5’-disodium phosphate-β-D-arabinofuranosyl)-1,2,3-triazoles (9 - 11) S6

Amine substitution to 6-chloropurine ribosides (13-18) S7

Phosphorylation to alkyl adenosine ribosides (19-24) S10

Isolation of X15ppX15 pyrophosphate S13

Supplementary data S14

NMR spectra S24

References S57

Experimental section.

General and Materials

^{1}H NMR and ^{13}C NMR spectra were recorded on a Bruker (400 MHz or 600 MHz) Instrument. Chemical shifts (*δ*) are reported in ppm and referenced to the residual solvent peaks. All fluorescence spectra were measured on a PerkinElmer LS 55 spectrophotometer at 25 °C using 1 mL cuvettes. Samples were dissolved in an appropriate solvent and measured in the same solvent. Data analysis was carried out using Origin 2019b. CD spectra were measured in a 1 mm quartz cuvette on a Jasco J-810 CD spectrometer equipped with a Julabo F12 temperature controller. TEM measurements were performed on a Zeiss EM10 microscope with an acceleration voltage of 120 kV, SEM on a ZEISS EVO MA microscope. Confocal microscopy images of all samples were recorded with a TCS SP8 confocal microscope using the Leica Application Suite X (LASX). DLS measurements were performed on a Nano-Zetasizer (Malvern Instruments) at 25 °C with a scattering angle of 173° and a wavelength of λ = 633 nm.

1,2,3,5-tetra-O-acetyl-*β*-D-ribofuranose, 6-chloropurine riboside, trimethylsilyl azide (95%), decylamine (95%), pentadecylamine (96%), dodecylamine (99%), octadecylamine (99%), 1-hexyne (97%), 1-dodecyne (98%), 1-hexadecyne (90%), phosphorus(V) oxychloride (99%), *N*-(3-dimethylaminopropyl)-*N'*-ethylcarbodiimide hydrochloride (EDC), 1-ethylimidazole (95%), trimethyl phosphate (99%), anhydrous ethanol, sodium methoxide (5.4 M in MeOH), $FeCl_3$, and K_2CO_3 were obtained from Sigma-Aldrich (Germany) or TCI (Germany) and used without further purification. All the solvents were analytical grade and used without further purification.

Synthesis

2,3,5-tri-*O*-acetyl-*β*-D-ribofuranosyl azide 2

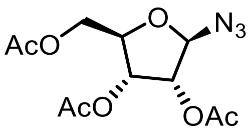

Under inert conditions, 1.00 g (3.14 mmol) **1** and 15.1 mg (0.09 mmol, 0.03 eq.) $FeCl_3$ was dissolved in 10 mL anhydrous DCM and stirred for 5 min. 0.63 mL (4.78 mmol, 1.5 eq.) Me_3SiN_3 were dissolved in 3 mL anhydrous DCM and the solution was added slowly under an inert atmosphere. The reaction was stirred at room temperature and monitored by TLC (EtOAc/Cyclohexane 1:2, R_f = 0.35). After 4 h, the reaction was quenched with a saturated $NaHCO_3$ solution. The two phases were separated, and the aqueous phase was extracted with DCM. The combined organic phases were washed with brine and

dried over $MgSO_4$ before DCM was removed. Drying under vacuum yielded 0.85 g (2.82 mmol, 90%) of a colorless oil. Spectra are in good agreement with the literature.[1]

^{1}H-NMR (400 MHz, $CDCl_3$): δ [ppm] = 2.07 (s, 3 H, OAc), 2.12 (2x s, 6 H, 2x OAc), 4.14 (dd, 1 H, *J* = 12.2 Hz, 4.3 Hz, 5'), 4.33 – 4.37 (m, 1 H, 4'), 4.41 (dd, 1 H, *J* = 12.2 Hz, 3.2 Hz, 5'), 5.13 (dd, 1 H, *J* = 4.8 Hz, 2.0 Hz, 3'), 5.31 – 5.37 (m, 2 H, 2' & 1').

^{13}C-NMR (100 MHz, $CDCl_3$): δ [ppm] = 20.6, 20.6, 20.8, 63.1, 70.6, 74.6, 79.5, 92.8, 169.5, 169.7, 170.7.

Huisgen cycloaddion to 4-Alkyl-1-(2',3',5'-tri-O-acetyl-*β*-D-ribofuranosyl)-1,2,3-triazoles 3 - 5

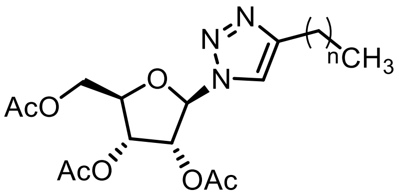

452 mg (1.50 mmol) **2** was dissolved in 20 mL anhydrous chloroform and degassed. 575 mg (3.00 mmol, 2 eq.) CuI and 1.31 mL (7.50 mmol, 5 eq.) DIPEA were added and the mixture was stirred for 5 min. 4.50 mmol (3 eq.) alkyne (1-octyne, 1-dodecyne, 1-hexadecyne, respectively) were added and the mixture was stirred for 2 d at 65 °C under an inert atmosphere. $CDCl_3$ was evaporated and direct column chromatography (DCM/EtOAc 7:1, R_f = 0.35, 0.4, 0.5, respectively) yielded between 70% and 93% of the products as colourless oils. Spectra are in good agreement with the literature.[2]

4-Hexyl-1-(2',3',5'-tri-O-acetyl-*β*-D-ribofuranosyl)-1,2,3-triazole 3 (n = 5)

^{1}H-NMR (400 MHz, $CDCl_3$): δ [ppm] = 0.87 (t, 3 H, *J* = 7.1 Hz, CH_3), 1.29-1.41 (br m, 6 H, 3 x CH_2), 1.66 (m, 2 H, CH_2), 2.08 (s, 3 H, OAc), 2.12 (2x s, 6 H, 2x OAc), 2.70 (t, 2 H, *J* = 7.9 Hz, CH_2), 4.22 (dd, 1 H, *J* = 12.2 Hz, 4.3 Hz, 5'), 4.40 (dd, 1 H, *J* = 12.2 Hz, 3.1 Hz, 5''), 4.46 (m, 1 H, 4'), 5.62 (t, 1 H, *J* = 5.4 Hz, 3'), 5.81 (dd, 1 H, *J* = 5.2 Hz, 3.9 Hz, 2'), 6.13 (d, 1 H, *J* = 3.8 Hz, 1'), 7.44 (s, 1 H, C=CH).

^{13}C-NMR (100 MHz, $CDCl_3$): δ [ppm] = 14.1, 20.5, 20.5, 22.7, 29.3, 29.4, 29.5, 29.6, 31.9, 62.9, 70.7, 74.3, 80.8, 169.3, 169.5, 170.4. Some signals overlap.

4-Decyl-1-(2',3',5'-tri-O-acetyl-β-D-ribofuranosyl)-1,2,3-triazole 4 (n = 9)

^{1}H-NMR (400 MHz, $CDCl_3$): δ [ppm] = 0.87 (t, 3 H, *J* = 7.1 Hz, CH_3), 1.22-1.33 (br m, 14 H, 7 x CH_2), 1.66 (m, 2 H, CH_2), 2.07 (s, 3 H, OAc), 2.11 (2x s, 6 H, 2x OAc), 2.70 (t, 2 H, *J* = 7.7 Hz, CH_2), 4.21 (dd, 1 H, *J* = 12.2 Hz, 4.3 Hz, 5'), 4.40 (dd, 1 H, *J* = 12.2 Hz, 3.1 Hz, 5''), 4.46 (m, 1 H, 4'), 5.61 (t, 1 H, *J* = 5.3 Hz, 3'), 5.80 (dd, 1 H, *J* = 5.2 Hz, 3.9 Hz, 2'), 6.11 (d, 1 H, *J* = 3.8 Hz, 1'), 7.44 (s, 1 H, C=CH).

^{13}C-NMR (100 MHz, $CDCl_3$): δ [ppm] = 14.1, 20.4, 20.5, 20.7, 21.2, 22.5, 22.6, 28.9, 29.1, 31.5, 62.9, 70.8, 74.3, 80.7, 169.3, 169.5, 170.4. Some signals overlap.

4-Tetradecyl-1-(2',3',5'-tri-O-acetyl-β-D-ribofuranosyl)-1,2,3-triazole 5 (n = 13)

^{1}H-NMR (400 MHz, $CDCl_3$): δ [ppm] = 0.88 (t, 3 H, *J* = 7.1 Hz, CH_3), 1.22-1.41 (br m, 22 H, 11 x CH_2), 1.67 (m, 2 H, CH_2), 2.08 (s, 3 H, OAc), 2.12 (2x s, 6 H, 2x OAc), 2.72 (t, 2 H, *J* = 7.7 Hz, CH_2), 4.22 (dd, 1 H, *J* = 12.2 Hz, 4.2 Hz, 5'), 4.41 (dd, 1 H, *J* = 12.2 Hz, 3.1 Hz, 5''), 4.46 (m, 1 H, 4'), 5.62 (t, 1 H, *J* = 5.4 Hz, 3'), 5.81 (dd, 1 H, *J* = 5.2 Hz, 3.9 Hz, 2'), 6.13 (d, 1 H, *J* = 3.8 Hz, 1'), 7.44 (s, 1 H, C=CH).

^{13}C-NMR (100 MHz, $CDCl_3$): δ [ppm] = 14.1, 20.5, 20.5, 20.7, 20.7, 25.6, 29.3, 29.3, 29.4, 29.6, 29.6, 29.7, 31.9, 62.9, 70.8, 74.3, 80.7, 89.8, 119.7, 169.3, 169.5, 170.4. Some signals overlap.

Deprotection to 4-Alkyl-1-(*β*-D-arabinofuranosyl)-1,2,3-triazoles 6 – 8

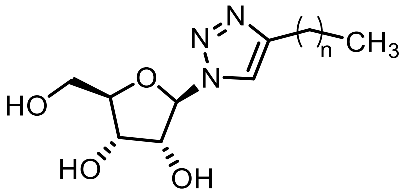

1.00 mmol of the 4-alkyl-1-(2',3',5'-tri-O-acetyl-*β*-D-ribofuranosyl)-1,2,3-triazoles **3 – 5** were dissolved in 10 – 25 mL anhydrous MeOH and 111 µL (0.60 mmol, 0.6 eq.) NaOMe in MeOH (5.4 M) were added. The mixture was stirred for 1.5 h at room temperature under an inert atmosphere before neutralizing it with 400 µL HCl/ether (2 M) (0.80 mmol, 0.8 eq.) and stirred for 15 min. Water was added and extracted with EtOAc. The organic phase was dried over $MgSO_4$. Evaporation of the solvents yielded 82 - 97% of the desired products as colorless to slightly yellow solids. Spectra were in good agreement with the literature.[3]

4-Hexyl-1-(β-D-arabinofuranosyl)-1,2,3-triazole 6 (n = 5)

^{1}H NMR (400 MHz, MeOH-d_4): δ [ppm] = 0.90 (t, 3 H, *J* = 6.9 Hz, CH_3), 1.29-1.41 (br m, 6 H, 3 x CH_2), 1.66 (m, 2 H, CH_2), 2.69 (t, 2 H, *J* = 7.7 Hz, CH_2), 3.67 (dd, 1 H, *J* = 12.2 Hz, 4.3 Hz, 5'), 3.80 (dd, 1 H, *J* = 12.2 Hz, 3.2 Hz, 5''), 4.10 (m, 1 H, 4'), 4.27 (t, 1 H, *J* = 5.0 Hz, 3'), 4.46 (t, 1 H, *J* = 4.5 Hz, 2'), 5.98 (d, 1 H, *J* = 4.1 Hz, 1'), 7.98 (1 H, s, C=CH).

^{13}C-NMR (100 MHz, MeOH-d_4): δ [ppm] = 14.2, 23.4, 26.1, 29.7, 30.3, 32.5, 62.7, 71.7, 76.8, 86.8, 94.0, 121.6, 149.2.

4-Decyl-1-(β-D-arabinofuranosyl)-1,2,3-triazole 7 (n = 9)

^{1}H NMR (400 MHz, MeOH-d_4): δ [ppm] = 0.88 (t, 3 H, *J* = 6.7 Hz, CH_3), 1.21-1.40 (br m, 14 H, 7 x CH_2), 1.66 (m, 2 H, CH_2), 2.69 (t, 2 H, *J* = 7.7 Hz, CH_2), 3.67 (dd, 1 H, *J* = 12.2 Hz, 4.3 Hz, 5'), 3.80 (dd, 1 H, *J* = 12.2 Hz, 3.1 Hz, 5''), 4.10 (m, 1 H, 4'), 4.27 (t, 1 H, *J* = 5.0 Hz, 3'), 4.46 (t, 1 H, *J* = 4.5 Hz, 2'), 5.98 (d, 1 H, *J* = 4.0 Hz, 1'), 7.98 (s, 1 H, C=CH).

^{13}C-NMR (100 MHz, MeOH-d$_4$): δ 14.5, 23.7, 26.3, 30.3, 30.5, 30.5, 30.5, 30.7, 30.7, 33.1, 62.9, 71.9, 77.0, 87.0, 94.2, 121.8, 149.4.

4-Tetradecyl-1-(β-D-arabinofuranosyl)-1,2,3-triazole 8 (n = 13)

^{1}H NMR (400 MHz, MeOH-d$_4$): δ [ppm] = 0.89 (t, 3 H, *J* = 6.8 Hz, CH_3), 1.22-1.38 (br m, 22 H, 11 x CH_2), 1.66 (m, 2 H, CH_2), 2.68 (t, 2 H, *J* = 7.7 Hz, CH_2), 3.67 (dd, 1 H, *J* = 12.2 Hz, 4.3 Hz, 5'), 3.80 (dd, 1 H, *J* = 12.2 Hz, 3.2 Hz, 5''), 4.10 (m, 1 H, 4'), 4.29 (t, 1 H, *J* = 5.0 Hz, 3'), 4.46 (t, 1 H, *J* = 4.5 Hz, 2'), 5.98 (d, 1 H, *J* = 4.1 Hz, 1'), 7.98 (s, 1 H, C=CH).

^{13}C-NMR (100 MHz, MeOH-d$_4$): δ [ppm] = 24.6, 23.7, 26.3, 30.3, 30.5, 30.6, 30.7, 30.8, 33.1, 62.89, 71.9, 77.0, 87.1, 94.3, 121.8, 149.4. Some signals overlap.

Phosphorylation to 4-Alkyl-1-(5'-disodium phosphate-β-D-arabinofuranosyl)-1,2,3-triazoles (9 - 11)

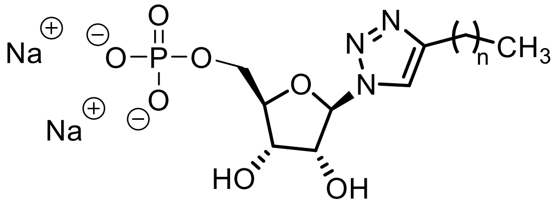

0.50 mmol of the 4-Alkyl-1-(*β*-D-arabinofuranosyl)-1,2,3-triazoles **6 - 8** were co-evaporated with MeCN three times and pre-dried in vacuo overnight. **6 - 8** were dissolved in 5 - 10 mL anhydrous $PO(OMe)_3$ (TMP) and stirred for 30 min at 0 °C. 56 µL (0.60 mmol, 1.2 eq.) $POCl_3$ were dissolved in 0.5 mL anhydrous $PO(OMe)_3$ and added to the mixture, which was stirred for 8 h at 0°C under an inert atmosphere. After 2 h another charge (0.6 mmol) of $POCl_3$ in $PO(OMe)_3$ was added. The mixture was quenched with 4 mL of H_2O, neutralized with Na_2CO_3 (pH ≈ 8) and stirred for 30 min at 0 °C. The precipitate was dissolved in H_2O. The aqueous phase was washed with ether three times. Purification was performed by MPLC (C18, H_2O/MeCN 98:2 – 5:95). Solvents were removed by lyophilization to obtain the desired products as colorless powders in 50 - 74% yield.

4-Hexyl-1-(5'-disodium phosphate-*β*-D-arabinofuranosyl)-1,2,3-triazole 9 (n = 5), C_6MP

^{1}H NMR (400 MHz, D_2O): δ [ppm] = 0.81 (t, 3 H, *J* = 6.5 Hz, CH_3), 1.19-1.31 (br m, 6 H, 3 x CH_2), 1.64 (m, 2 H, CH_2), 2.74 (t, 2 H, *J* = 7.4 Hz, CH_2), 4.00 – 4.12 (br m, 2 H, 5' & 5''), 4.37 (m, 1 H, 4'), 4.44 (t, 1 H, *J* = 4.5 Hz, 3'), 4.64 (t, 1 H, *J* = 5.1 Hz, 2'), 6.12 (d, 1 H, *J* = 5.2 Hz, 1'), 8.15 (s, 1 H, C=CH).

^{13}C-NMR (100 MHz, D_2O): δ [ppm] = 12.4, 20.9, 23.5, 26.8, 27.5, 29.8, 63.6, 69.6, 74.0, 83.4, 91.0, 120.2, 148.5.

^{31}P-NMR (162 MHz, HEPES buffer pH = 7.50, against 85% H_3PO_4): δ [ppm] = 3.78.

HRMS ESI(-) ($C_{13}H_{23}N_3O_7P$, 364.12791): m/z = 364.12794 ($[M]^-$) (Δ = 0.08 ppm).

4-Decyl-1-(5'-disodium phosphate-*β*-D-arabinofuranosyl)-1,2,3-triazole 10 (n = 9), C_{10}MP

^{1}H NMR (400 MHz, D_2O): δ [ppm] = 0.78 (t, 3 H, *J* = 6.6 Hz, CH_3), 1.13-1.30 (br m, 14 H, 7 x CH_2), 1.55 (m, 2 H, CH_2), 2.58 (t, 2 H, *J* = 7.6 Hz, CH_2), 3.93 – 4.05 (br m, 2 H, 5' & 5''), 4.28 (m, 1 H, 4'), 4.44 (t, 1 H, *J* = 5.0 Hz, 3'), 4.59 (t, 1 H, *J* = 4.7 Hz, 2'), 6.00 (d, 1 H, *J* = 4.2 Hz, 1'), 7.84 (s, 1 H, C=CH).

^{13}C-NMR (100 MHz, D_2O): δ [ppm] = 13.4, 22.1, 24.5, 28.1, 28.4, 28.5, 28.5, 28.7, 28.7, 31.2, 64.2, 70.7, 74.9, 84.5, 91.8, 121.2, 149.4.

^{31}P-NMR (162 MHz, HEPES buffer pH = 7.50, against 85% H_3PO_4): δ [ppm] = 3.73.

HRMS ESI(-) ($C_{17}H_{31}N_3O_7P$, 420.19051): m/z = 420.19070 ($[M]^-$) (Δ = 0.45 ppm).

4-Tetradecyl-1-(5'-disodium phosphate-*β*-D-arabinofuranosyl)-1,2,3-triazole 11 (n = 13), C_{14}MP

^{1}H NMR (400 MHz, D_2O): δ [ppm] = 0.83 (t, 3 H, *J* = 6.5 Hz, CH_3), 1.15-1.35 (br m, 22 H, 11 x CH_2), 1.59 (m, 2 H, CH_2), 2.69 (t, 2 H, *J* = 7.4 Hz, CH_2), 3.88 – 4.01 (br m, 2 H, 5' & 5''), 4.30 (m, 1 H, 4'), 4.45 (t, 1 H, *J* = 4.5 Hz, 3'), 4.62 (m, 1 H, 2'), 6.00 (d, 1 H, *J* = 5.2 Hz, 1'), 7.86 (s, 1 H, C=CH).

^{13}C-NMR (100 MHz, MeOH-d_4): δ 13.0, 22.3, 25.0, 29.0, 29.1, 29.1, 29.3, 29.4, 29.4, 29.4, 31.7, 64.4, 71.2, 75.8, 85.0, 92.9, 119.9, 148.8. Some signals overlap.

^{31}P-NMR (162 MHz, MeOH-d_4, against 85% H_3PO_4): δ [ppm] = 2.12.

HRMS ESI(-) ($C_{21}H_{39}N_3O_7P$, 476.25311): m/z = 476.25320 ($[M]^-$) (Δ = 0.19 ppm).

Amine substitution to 6-chloropurine ribosides (13-18)

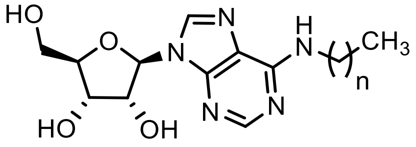

Compounds **13-18** were prepared as previously described.[4]

To a solution of 500 mg 6-chloropurine riboside **12** (1.74 mmol, 1.0 eq.) in anhydrous EtOH (10 mL), 1-alkyl amine (8.7 mmol, 5.0 eq.) was added under N_2 atmosphere and stirred at 90°C until the reaction was finished. After cooling, the crude product precipitated and was filtered off, washed with cold EtOH, and dried. The pure product was obtained either by washing the crude product with Et_2O or column chromatography with DCM:MeOH (10:1).

2-(6-(decylamino)-9H-purin-9-yl)-5-(hydroxymethyl)tetrahydrofuran-3,4-diol (n = 9)

^{1}H NMR (500 MHz, DMSO-*d*$_6$) δ [ppm] = 8.33 (s, 1H, H-8), 8.20 (s, 1H, H-2), 7.84 (s, 1H, NH), 5.90 (d, *J* = 6.2 Hz, 1H, H-1'), 5.46 (dd, *J* = 7.3, 4.3 Hz, 1H, OH-5'), 5.43 (d, *J* = 6.3 Hz, 1H, OH-2'), 5.18 (d, *J* = 4.6 Hz, 1H, OH-3'), 4.62 (q, *J* = 5.9 Hz, 1H, H-2'), 4.17 (td, *J* = 4.8, 3.0 Hz, 1H, H-3'), 3.99 (q, *J* = 3.4 Hz, 1H, H-4'), 3.69 (dt, *J* = 12.1, 3.9 Hz, 1H, H-5'), 3.57 (ddd, *J* = 11.6, 7.2, 3.5 Hz, 1H, H-5'), 3.51-3.41 (m, 2H, $C\underline{H}_2NH$), 1.58 (p, *J* = 7.0 Hz, 2H, $C\underline{H}_2CH_2NH$), 1.30-1.17 (m, 14H, $(CH_2)_7$), 0.86-0.80 (t, *J* = 6.8 Hz, 3H, CH_3).

^{13}C NMR (126 MHz, DMSO-*d*$_6$) δ [ppm] = 154.7 (C-6), 152.3 (C-2), 148.1 (C-4), 139.6 (C-8), 119.8 (C-5), 88.1 (C-1'), 86.0 (C-4'), 73.6 (C-2'), 70.7 (C-3'), 61.7 (C-5'), 38.9 ($\underline{C}H_2NH$), 31.3 ($\underline{C}H_2CH_2NH$), 29.1 (CH_2), 29.0 (CH_2), 28.9 (CH_2), 28.8 (CH_2), 28.7 (CH_2), 26.4 (CH_2), 22.1 (CH_2), 13.9 (CH_3).

2-(6-(dodecylamino)-9H-purin-9-yl)-5-(hydroxymethyl)tetrahydrofuran-3,4-diol (n= 11)

^{1}H NMR (500 MHz, DMSO-*d*$_6$) δ [ppm] = 8.34 (s, 1H, H-8), 8.20 (s, 1H, H-2), 7.84 (s, 1H, NH), 5.90 (d, *J* = 6.1 Hz, 1H, OH-5'), 5.46 (dd, *J* = 7.4, 4.4 Hz, 1H, OH-2'), 5.43 (d, *J* = 6.2 Hz, 1H, OH-3'), 5.18 (d, *J* = 4.5 Hz, 1H, H-1'), 4.63 (q, *J* = 5.8 Hz, 1H, H-2'), 4.18 (td, *J* = 4.7, 2.9 Hz, 1H, H-3'), 3.99 (q, *J* = 3.3 Hz, 1H, H-4'), 3.70 (dt, *J* = 12.1, 4.0 Hz, 1H, H-5'), 3.58 (ddd, *J* = 12.1, 7.4, 3.5 Hz, 1H, H-5'), 3.46 (dd, *J* = 7.0, 5.1 Hz, 2H, $C\underline{H}_2NH$), 1.59 (p, *J* = 7.0 Hz, 2H, $C\underline{H}_2CH_2NH$), 1.31-1.19 (m, 18H, $(CH_2)_9$), 0.87-0.81 (t, *J* = 7.5 Hz, 3H, CH_3).

^{13}C NMR (126 MHz, DMSO-*d*$_6$) δ [ppm] = 154.7 (C-6), 152.3 (C-2), 148.2 (C-4), 139.6 (C-8), 119.8 (C-5), 88.1 (C-1'), 86.0 (C-4'), 73.6 (C-2'), 70.7 (C-3'), 61.7 (C-5'), 38.9 ($\underline{C}H_2NH$), 31.3 ($\underline{C}H_2CH_2NH$), 29.1 (CH_2), 29.0 (CH_2), 28.9 (CH_2), 28.9 (CH_2), 28.8 (CH_2), 28.8 (CH_2), 28.7 (CH_2), 26.4 (CH_2), 22.1 (CH_2), 13.9 (CH_3).

2-(6-(tridecylamino)-9H-purin-9-yl)-5-(hydroxymethyl)tetrahydrofuran-3,4-diol (n= 12)

^{1}H NMR (400 MHz, DMSO-*d*$_6$) δ [ppm] = 8.33 (s, 1H, H-8), 8.19 (s, 1H, H-2), 7.86 (s, 1H, NH), 5.88 (d, *J* = 6.1 Hz, 1H, OH-5'), 5.43 (m, 1H, OH-2'), 5.43 (m, 1H, OH-3'), 5.18 (m, 1H, H-1'), 4.62 (m, 1H, H-2'), 4.16 (m, 1H, H-3'), 3.97 (q, *J* = 3.3 Hz, 1H, H-4'), 3.68 (d, *J* = 12.1, 1H, H-5'), 3.55 (m, 1H, H-5'), 3.45 (m, 2H, $C\underline{H}_2NH$), 1.58 (p, *J* = 7.0 Hz, 2H, $C\underline{H}_2CH_2NH$), 1.33-1.16 (m, 20H, $(CH_2)_{10}$), 0.87-0.81 (t, *J* = 7.5 Hz, 3H, CH_3).

^{13}C NMR (100 MHz, DMSO-*d*$_6$) δ [ppm] = 154.7 (C-6), 152.4 (C-2), 148.2 (C-4), 139.6 (C-8), 119.8 (C-5), 88.0 (C-1'), 85.9 (C-4'), 73.5 (C-2'), 70.7 (C-3'), 61.7 (C-5'), 31.3 ($\underline{C}H_2CH_2NH$), 29.1 (CH_2), 29.0 (CH_2), 28.9 (CH_2), 28.7 (CH_2), 26.4 (CH_2), 22.1 (CH_2), 13.9 (CH_3). Some signals overlap.

2-(hydroxymethyl)-5-(6-(tetradecylamino)-9H-purin-9-yl)tetrahydrofuran-3,4-diol (n = 13)

^{1}H NMR (500 MHz, DMSO-*d*$_6$) δ [ppm] = 8.33 (s, 1H, H-8), 8.19 (s, 1H, H-2), 7.83 (s, 1H, NH), 5.90 (d, *J* = 6.1 Hz, 1H, H-1'), 5.46 (dd, *J* = 7.4, 4.4 Hz, 1H, OH-5'), 5.42 (d, *J* = 6.3 Hz, 1H, OH-2'), 5.17 (d, *J* = 4.7 Hz, 1H, OH-3'), 4.62 (q, *J* = 5.9 Hz, 1H, H-2'), 4.17 (td, *J* = 4.8, 2.9 Hz, 1H, H-3'), 3.99 (q, *J* = 3.3 Hz, 1H, H-4'), 3.69 (dt, *J* = 12.2, 4.0 Hz, 1H, H-5'), 3.57 (ddd, *J* = 11.6, 7.4, 3.5 Hz, 1H, H-5'), 3.46 (m, 2H, CH_2NH), 1.58 (p, *J* = 7.1 Hz, 2H, CH_2CH_2NH), 1.21 (m, 22H, $(CH_2)_{11}$), 0.83 (t, *J* = 6.8 Hz, 3H, CH_3).
^{13}C NMR (126 MHz, DMSO-*d*$_6$) δ [ppm] = 154.7 (C-6), 152.3 (C-2), 148.2 (C-4), 139.6 (C-8), 119.8 (C-5), 88.1 (C-1'), 86.0 (C-4'), 73.6 (C-2'), 70.7 (C-3'), 61.7 (C-5'), 39.0 (CH_2NH), 31.3 (CH_2CH_2NH), 29.2 (CH_2), 29.1 (CH_2), 28.9 (CH_2), 28.9 (CH_2), 28.8 (CH_2), 28.7 (CH_2), 28.69 (CH_2), 28.6 (CH_2), 26.5 (CH_2), 22.1 (CH_2), 22.0 (CH_2),13.9 (CH_3).

2-(hydroxymethyl)-5-(6-(pentadecylamino)-9H-purin-9-yl)tetrahydrofuran-3,4-diol (n = 14)

^{1}H NMR (600 MHz, DMSO-*d*$_6$) δ [ppm] = 8.33 (s, 1H, H-8), 8.21 (s, 1H, H-2), 7.83 (s, 1H, NH), 5.87 (d, J = 6.2 Hz, 1H, H-1'), 5.43 (s, 2H, OH-5' and OH-2'), 5.23 - 5.12 (m, 1H, OH-3'), 4.61 (t, J = 5.6 Hz, 1H, H-2'), 4.14 (dd, J = 5.0, 3.0 Hz, 1H, H-3'), 3.96 (q, J = 3.4 Hz, 1H, H-4'), 3.67 (dd, J = 12.1, 3.6 Hz, 1H, H-5'), 3.55 (dd, J = 12.1, 3.6 Hz, 1H, H-5'), 3.49 – 3.42 (m, 2H, CH_2NH), 1.57 (p, J = 7.1 Hz, 2H, CH_2CH_2NH), 1.23 (d, J = 3.3 Hz, 24H, $(CH_2)_{12}$), 0.84 (t, *J* = 6.9 Hz, 3H, CH_3).
^{13}C NMR (151 MHz, DMSO-*d*$_6$) δ [ppm] = 154.7 (C-6), 152.4 (C-2), 148.2 (C-4), 139.6 (C-8), 119.7 (C-5), 88.0 (C-1'), 85.9 (C-4'), 73.5 (C-2'), 70.7 (C-3'), 61.7 (C-5'), 39.1 (CH_2NH), 31.3 (CH_2CH_2NH), 29.1 (CH_2), 29.0 (CH_2), 28.8 (CH_2), 28.7 (CH_2), 26.4 (CH_2), 26.3 (CH_2), 22.1 (CH_2), 13.9 (CH_3).

2-(6-(heptadecylamino)-9H-purin-9-yl)-5-(hydroxymethyl)tetrahydrofuran-3,4-diol (n = 17)

^{1}H NMR (500 MHz, DMSO-*d*$_6$) δ [ppm] = 8.33 (s, 1H, H-8), 8.19 (s, 1H, H-2), 7.88 (s, 1H, NH), 5.87 (d, *J* = 6.2 Hz, 1H, H-1'), 5.44 (dd, *J* = 9.1, 5.3 Hz, 2H, OH-2' & OH-5'), 5.18 (d, *J* = 4.6 Hz, 1H, OH-3'), 4.60 (q, *J* = 5.7 Hz, 1H, H-2'), 4.14 (td, *J* = 4.8, 2.9 Hz, 1H, H-3'), 3.96 (q, *J* = 3.4 Hz, 1H, H-4'), 3.67 (dt, *J* = 12.1, 4.1 Hz, 1H, H-5'), 3.55 (ddd, *J* = 11.6, 7.2, 3.6 Hz, 1H, H-5'), 3.51-3.40 (m, 2H, CH_2NH), 1.57 (p, *J* = 6.8 Hz, 2H, CH_2CH_2NH), 1.22 (m, 30H, $(CH_2)_{15}$), 0.88-0.80 (t, *J* = 6.0 Hz, 3H, CH_3).
^{13}C NMR (126 MHz, DMSO-*d*$_6$) δ [ppm] = 154.6 (C-6), 151.9 (C-2), 148.5 (C-4), 139.0 (C-8), 119.3 (C-5), 87.9 (C-1'), 85.6 (C-4'), 73.4 (C-2'), 70.3 (C-3'), 61.4 (C-5'), 38.8 (CH_2NH), 30.9 (CH_2CH_2NH), 28.9 (CH_2-CH_2), 28.6 (CH_2-CH_2), 28.5 (CH_2-CH_2), 28.5 (CH_2-CH_2), 28.4 (CH_2-CH_2), 28.4 (CH_2-CH_2), 28.3 (CH_2-CH_2), 28.2 (CH_2-CH_2), 28.2 (CH_2-CH_2), 28.1 (CH_2-CH_2), 26.9 (CH_2-CH_2), 26.0 (CH_2-CH_2), 25.5 (CH_2-CH_2), 21.6 (CH_2-CH_2), 21.5 (CH_2-CH_2), 13.3 (CH_3).

Phosphorylation to alkyl adenosine ribosides

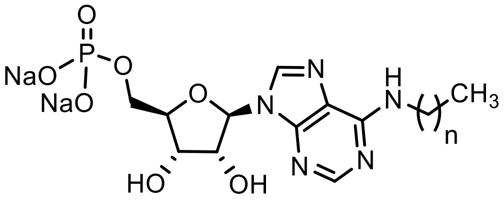

0.50 mmol of the compound **13 - 18** were co-evaporated with MeCN three times and pre-dried in vacuo overnight. **13 - 18** were dissolved in 10 mL anhydrous TMP and stirred for 30 min at 0 °C. 1.5-2.0 eq. $POCl_3$ were added to the mixture and stirred for 3-8 h at 0°C under an inert atmosphere. After the starting material was consumed completely (monitored by TLC), the reaction mixture was quenched with 5 mL of H_2O, neutralized with Na_2CO_3, stirred for 30 min at 0 °C, and washed with Et_2O (5 × 10 mL). The aqueous phase was further purified by MPLC (C18, H_2O/MeCN 95:5 – 5:95). Solvents were removed by lyophilization to obtain the desired products as white powders in 50 - 70% yield.

Note: The chemical shift and the signal broadness (and multiplicity) of compounds **19 - 24** depend strongly on the concentration and may therefore vary.

X_{10}MP

^{1}H NMR (400 MHz, D_2O) δ [ppm] = 8.23 (s, 1H, H-8), 8.04 (s, 1H, H-2), 5.91 (d, *J* = 4.8 Hz, 1H, H-1'), 4.53 (t, *J* = 5.0 Hz, 1H, H-2'), 4.37 (t, *J* = 4.8 Hz, 1H, H-3'), 4.22 (q, *J* = 4.0 Hz, 1H, H-4'), 4.02 (q, *J* = 7.3, 6.7 Hz, 2H, H-5'), 3.31 (s, 2H, C<u>H</u>$_2$NH), 1.48 (q, *J* = 8.0, 7.5 Hz, 2H, C<u>H</u>$_2$CH$_2$NH), 1.15 (d, *J* = 20.0 Hz, 14H, $(CH_2)_7$), 0.77 (t, *J* = 6.6 Hz, 3H, CH_3).

^{13}C NMR (101 MHz, D_2O) δ 154.1 (C-6), 152.7 (C-2), 147.4 (C-4), 138.4 (C-8), 118.5 (C-5), 87.1 (C-1'), 83.5 (C-4'), 74.5 (C-2'), 70.1 (C-3'), 64.1 (C-5'), 40.6 (<u>C</u>H$_2$NH), 31.8 (<u>C</u>H$_2$CH$_2$NH), 29.7 (CH_2), 29.6 (CH_2), 29.4 (CH_2), 29.3 (CH_2), 29.1 (CH_2), 26.9 (CH_2), 22.5 (CH_2), 13.7 (CH_3).

^{31}P NMR (162 MHz, D_2O) δ [ppm] = 1.88.

HRMS ESI(-) ($C_{20}H_{33}N_5O_7P^-$, 486.2123): m/z = 486.2136 ($[M]^-$) (Δ = 2.67 ppm).

X_{12}MP

^{1}H NMR (400 MHz, D_2O) δ [ppm] = 8.26 (s, 1H, H-8), 8.03 (s, 1H, H-2), 5.91 (d, J = 4.7 Hz, 1H, H-1'), 4.55 (t, J = 5.0 Hz, 1H, H-2'), 4.35 (t, J = 4.8 Hz, 1H, H-3'), 4.22 (q, J = 4.3 Hz, 1H, H-4'), 3.94 (tt, J = 12.7, 5.2 Hz, 2H, H-5'), 3.32 (dt, J = 23.4, 10.3 Hz, 2H, C<u>H</u>$_2$NH), 1.51 (p, J = 7.4, 6.8 Hz, 2H, C<u>H</u>$_2$CH$_2$NH), 1.18 (s, 18H, $(CH_2)_9$), 0.81 (t, J = 6.6 Hz, 3H, CH_3).

^{13}C NMR (101 MHz, D_2O) δ [ppm] = 154.2 (C-6), 152.8 (C-2), 147.46 (C-4), 138.6 (C-8), 118.5 (C-5), 87.0 (C-1'), 83.7 (C-4'), 74.3 (C-2'), 70.3 (C-3'), 63.8 (C-5'), 40.6 (<u>C</u>H$_2$NH), 31.9 (<u>C</u>H$_2$CH$_2$NH), 29.9 (CH_2),

29.8 (CH_2), 29.8 (CH_2), 29.5 (CH_2), 29.4 (CH_2), 29.2 (CH_2), 29.1 (CH_2), 27.0 (CH_2), 22.6 (CH_2), 13.8 (CH_3).

^{31}P NMR (162 MHz, D_2O) δ [ppm] = 4.02.

HRMS ESI(-) ($C_{22}H_{37}N_5O_7P^-$, 514.2436): m/z = 514.2465 ($[M]^-$) (Δ = 5.64 ppm).

X_{13}MP

^{1}H NMR (600 MHz, D_2O) δ [ppm] = 8.34 (s, 1H, H-8), 8.10 (s, 1H, H-2), 5.98 (s, 1H, H-1'), 4.62 (s, 1H, H-2'), 4.41 (s, 1H, H-3'), 4.27 (s, 1H, H-4'), 3.97 (s, 2H, H-5'), 3.41 (d, J = 32.0 Hz, 2H, C$\underline{H_2}$NH), 1.55 (s, 2H, C$\underline{H_2}$$CH_2$NH), 1.18 (s, 20H, $(CH_2)_{10}$), 0.80 (s, 3H, CH_3).

^{13}C NMR (150 MHz, D_2O) δ [ppm] = 154.4 (C-6), 152.8 (C-2), 147.6 (C-4), 138.7 (C-8), 118.7 (C-5), 87.0 (C-1'), 83.8 (C-4'), 74.3 (C-2'), 70.4 (C-3'), 63.7 (C-5'), 40.6 ($\underline{C}H_2$NH), 31.8 ($\underline{C}H_2CH_2$NH), 29.7 (CH_2), 29.3 (CH_2), 29.1 (CH_2), 26.8 (CH_2), 22.5 (CH_2), 13.8 (CH_3). Some signals overlap.

^{31}P NMR (243 MHz, D_2O) δ [ppm] = 4.02.

HRMS ESI(-) ($C_{22}H_{37}N_5O_7P^-$, 528.25930): m/z = 528.25931 ($[M]^-$) (Δ = 0.02 ppm).

X_{14}MP

^{1}H NMR (400 MHz, D_2O) δ [ppm] = 8.16 (s, 1H, H-8), 7.94 (s, 1H, H-2), 5.81 (s, 1H, H-1'), 4.44 (s, 1H, H-2'), 4.25 (s, 1H, H-3'), 4.12 (s, 1H, H-4'), 3.84 (d, J = 15.3 Hz, 2H, H-5'), 3.22 (s, 2H, C$\underline{H_2}$NH), 1.43 (s, 2H, C$\underline{H_2}$$CH_2$NH), 1.14 (s, 22H, $(CH_2)_{11}$), 0.75 (t, J = 6.1 Hz, 3H, CH_3).

^{13}C NMR (101 MHz, D_2O) δ [ppm] = 154.2 (C-6), 152.8 (C-2), 147.5 (C-4), 138.6 (C-8), 118.5 (C-5), 87.0 (C-1'), 83.7 (C-4'), 74.3 (C-2'), 70.3 (C-3'), 63.8 (C-5'), 40.7 ($\underline{C}H_2$NH), 32.0 ($\underline{C}H_2CH_2$NH), 30.0 (CH_2), 30.0 (CH_2), 29.7 (CH_2), 29.6 (CH_2), 29.5 (CH_2), 29.44(CH_2), 29.4 (CH_2), 29.3 (CH_2), 29.2 (CH_2), 27.1 (CH_2), 22.6 (CH_2), 13.8 (CH_3).

^{31}P NMR (162 MHz, D_2O) δ [ppm] = 4.07.

HRMS ESI(-) ($C_{24}H_{41}N_5O_7P^-$, 542.2749): m/z = 542.2742 ($[M]^-$) (Δ = 1.29 ppm).

X_{15}MP

^{1}H NMR (400 MHz, D_2O) δ [ppm] = 8.28 (s, 1H, H-8), 8.06 (s, 1H, H-2), 5.93 (s, 1H, H-1'), 4.56 (s, 1H, H-2'), 4.36 (s, 1H, H-3'), 4.23 (s, 1H, H-4'), 3.95 (s, 2H, H-5'), 3.34 (s, 2H, C$\underline{H_2}$NH), 1.53 (s, 2H, C$\underline{H_2}$$CH_2$NH), 1.22 (s, 24H, $(CH_2)_{12}$), 0.83 (s, 3H, CH_3).

^{13}C NMR (101 MHz, D_2O) δ [ppm] = 154.2 (C-6), 152.8 (C-2), 147.5 (C-4), 138.6 (C-8), 118.6 (C-5), 87.0 (C-1'), 83.7 (C-4'), 74.3 (C-2'), 70.3 (C-3'), 63.8 (C-5'), 40.6 ($\underline{C}H_2$NH), 32.0 ($\underline{C}H_2CH_2$NH), 30.0 (CH_2), 29.9 (CH_2), 29.5 (CH_2), 29.2 (CH_2), 27.0 (CH_2), 22.6 (CH_2), (CH_3). Some signals overlap.

^{31}P NMR (162 MHz, D_2O) δ [ppm] = 4.08.

HRMS ESI(-) ($C_{25}H_{43}N_5O_7P^-$, 556.2906): m/z = 556.2901 ($[M]^-$) (Δ = 0.90 ppm).

X_{18}MP

^{1}H NMR (400 MHz, D_2O) δ [ppm] = 8.20 (s, 1H, H-8), 8.01 (s, 1H, H-2), 5.85 (s, 1H, H-1'), 4.47 (s, 1H, H-2'), 4.30 (s, 1H, H-3'), 4.16 (s, 1H, H-4'), 3.91 (s, 2H, H-5'), 3.30 (s, 2H, C$\underline{H}_2$NH), 1.54 (s, 2H, C$\underline{H}_2$$CH_2$NH), 1.29 (s, 30H, $(CH_2)_{15}$), 0.88 (s, 3H, CH_3).

^{13}C NMR (101 MHz, D_2O) δ [ppm] = 154.1 (C-6), 152.7 (C-2), 147.4 (C-4), 138.4 (C-8), 118.5 (C-5), 87.0 (C-1'), 83.5 (C-4'), 74.3 (C-2'), 70.2 (C-3'), 63.8 (C-5'), 40.7 ($\underline{C}H_2$NH), 32.2 ($\underline{C}H_2CH_2$NH), 30.5 (CH_2), 29.8 (CH_2), 29.2 (CH_2), 27.30 (CH_2), 22.8 (CH_2), 13.86 (CH_3). Some signals overlap.

^{31}P NMR (162 MHz, D_2O) δ [ppm] = 4.18.

HRMS ESI(-) ($C_{28}H_{49}N_5O_7P^-$, 598.3375): m/z = 598.3399 ($[M]^-$) (Δ = 3.68 ppm).

Isolation of X15ppX15 pyrophosphate

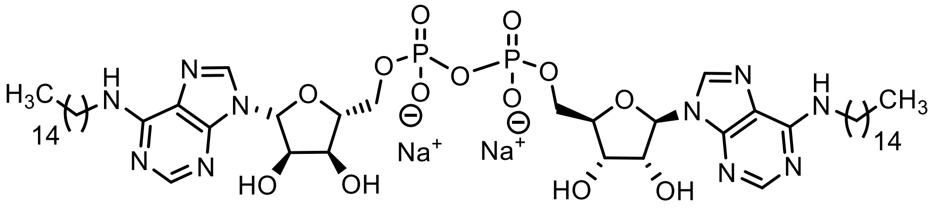

After dimerization of X_{15}MP with 50 eq. EDC in condensation buffer for 6 h (according to the standard procedure), **X15ppX15** was pre-purified by MPLC on an Interchim PF-15C18HP-F0025 reverse phase column and finally purified by semi-preparative HPLC on a Supelco Ascentis C8 (25cm x 10 mm, 5 µm) column with a mobile phase of MeCN / 50 mM NH_4HCO_3 (7:3) and a flow rate of 3.0 mL/min at 60 °C (retention time: 9-12 min). The product was dried by lyophilization.

^{1}H NMR (600 MHz, MeOH-d$_4$) δ [ppm] = 8.71 (s, 2H, H-8), 8.46 (s, 2H, H-2), 6.29 (d, J=4.8 Hz, 2H, H-1'), 5.72 (s, 2H, NH), 4.83 (s, 2H, H-2'), 4.73 (t, ^3J=4.1 Hz, 2H, H-3'), 4.56 (m, 4H, H5'), 4.51 (m, 2H, H-4'), 3.87 (m, 4H, CH_2), 1.91 (m, 4H, CH_2), 1.69 – 1.45 (m, 48 H, CH_2), 1.14 (t, ^3J=7.1 Hz, 6H, CH_3).
^{13}C NMR (150 MHz, MeOH-d$_4$) δ [ppm] = 140.8, 120.3, 89.7, 85.6, 76.7, 71.8, 66.5, 54.8, 33.1, 30.9, 30.7, 30.6, 30.5, 28.1, 23.8. 14.5. Some quartinary carbon signals are not visible due to poor solubility of the compound, some signals overlap.
^{31}P NMR (243 MHz, MeOH-d$_4$) δ [ppm] = -11.01.
HRMS ESI(-) ($C_{50}H_{84}N_{10}O_{13}P_2^-$, 1095.5773): m/z = 1095.5841 ($[M]^-$) (Δ = 6.21 ppm).

Supplementary data

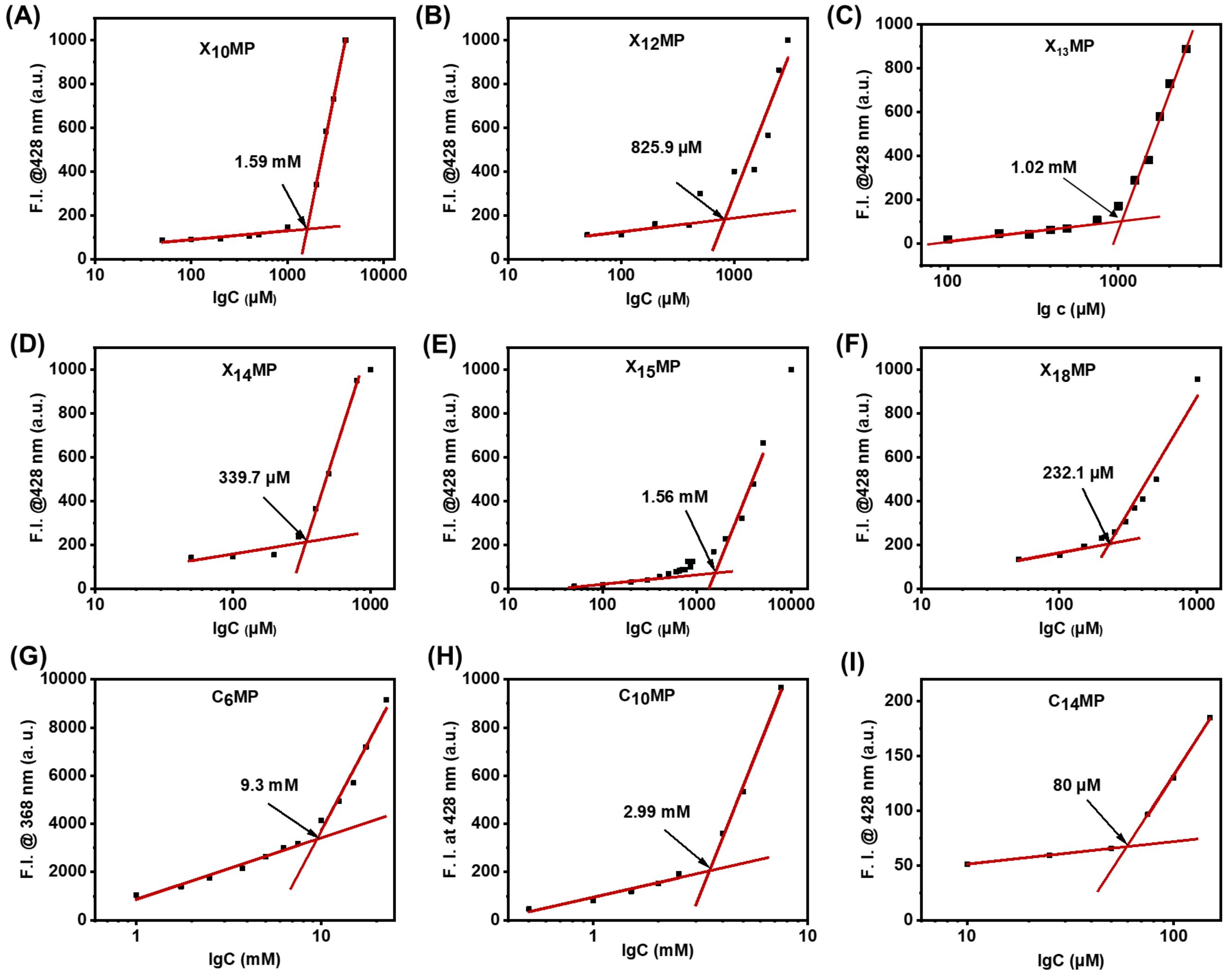

Figure S1. The CAC of all ribonucleotide-based amphiphiles was determined by monitoring the fluorescence intensity of DPH dye (10µM) at 428 nm in a dilution series of amphiphiles. Aggregation of the amphiphiles leads to incorporation of the dye in the apolar regions of the aggregates and hence leads to increasing fluorescence.

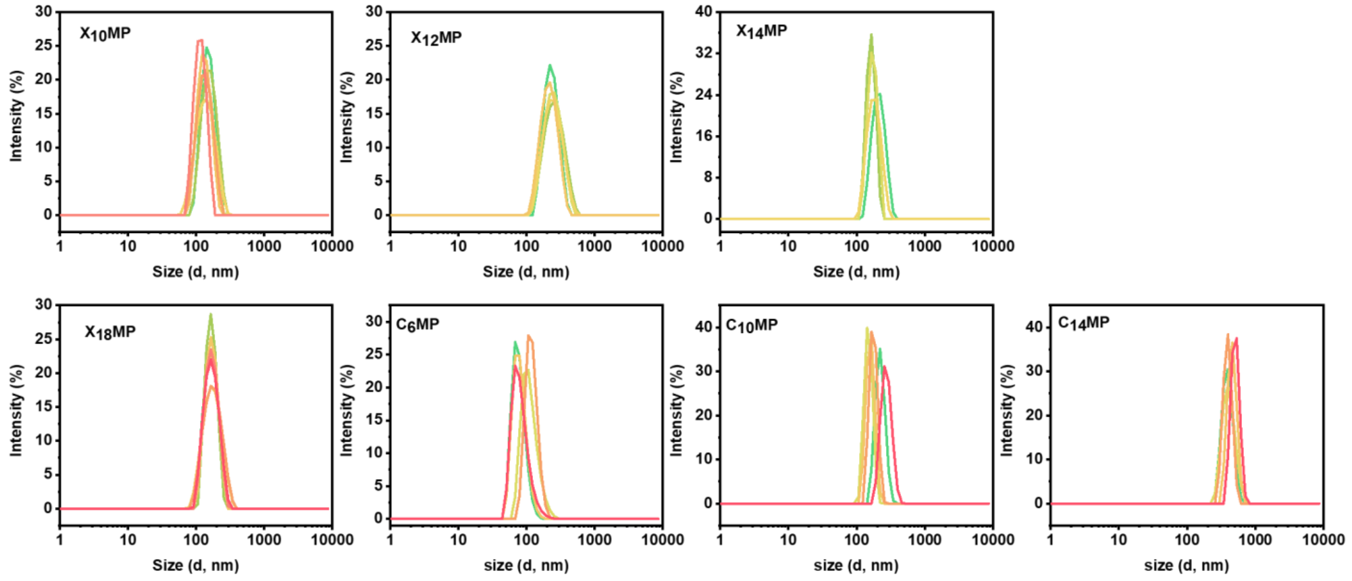

Figure S2. The hydrodynamic diameters of ribonucleotide-based amphiphiles were determined by DLS. Amphiphiles were dissolved in 5 mM HEPES buffer in concentrations above the respective CAC (**C_6MP**: 18 mM, **C_{10}MP**: 6 mM, **C_{14}MP**: 160 µM, **X_{10}MP**: 2.0 mM, **X_{12}MP**: 1.3 mM, **X_{14}MP:** 2.2 mM, **X_{18}MP**: 0.5 mM).

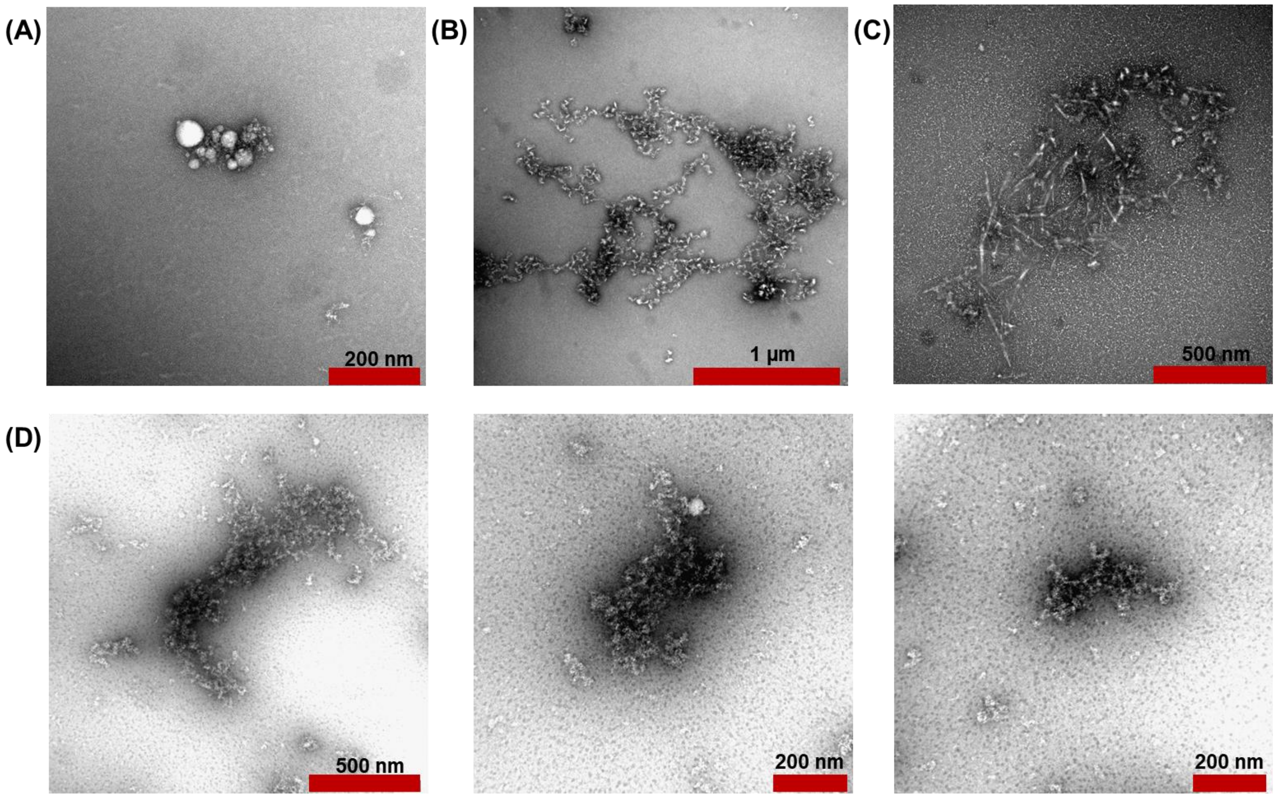

Figure S3: The additional TEM images of (**A**) C_6MP, (**B**) C_{10}MP, (**C**) C_{14}MP and (**D**) X_{13}MP in HEPES buffer (pH = 7.50).

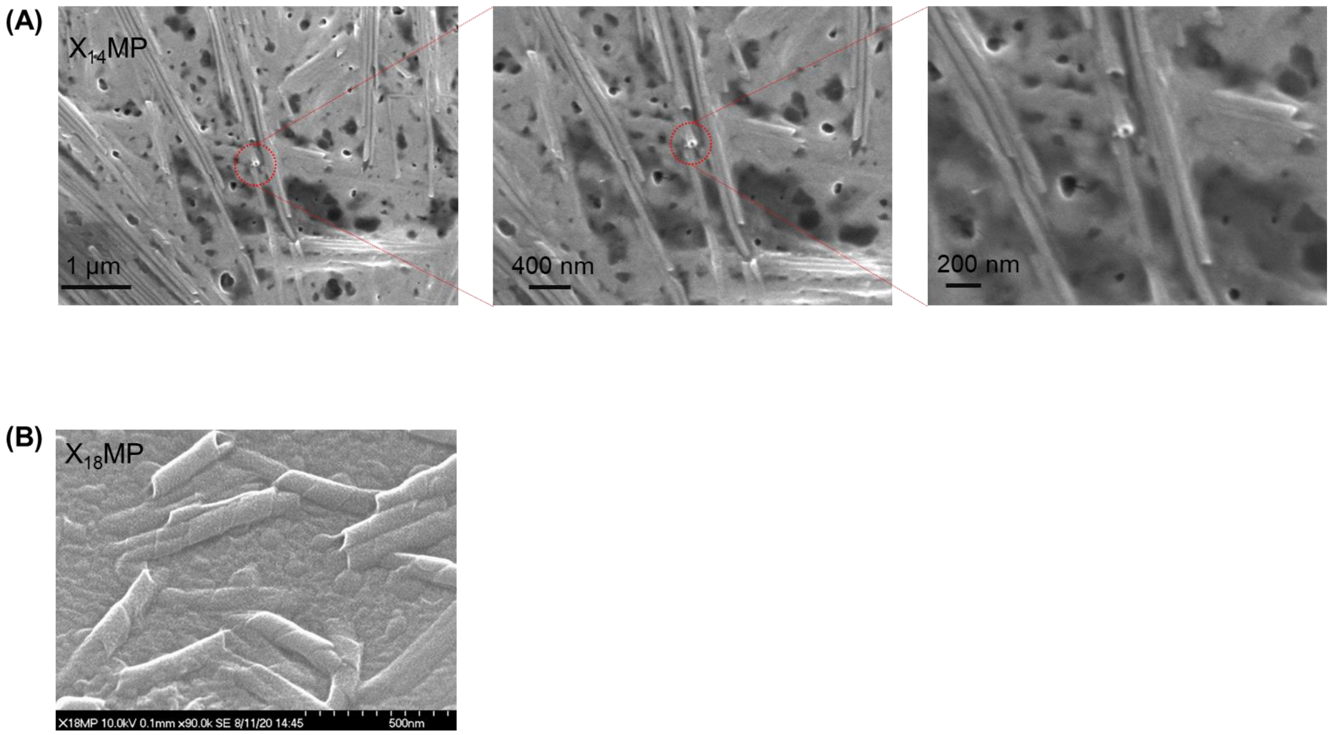

Figure S4. SEM images of (**A**) $X_{14}MP$ and (**B**), $X_{18}MP$, respectively. The SEM images clearly indicate that $X_{14}MP$ self-assembles into hollow tube structures and $X_{18}MP$ self-assembles into helical ribbons.

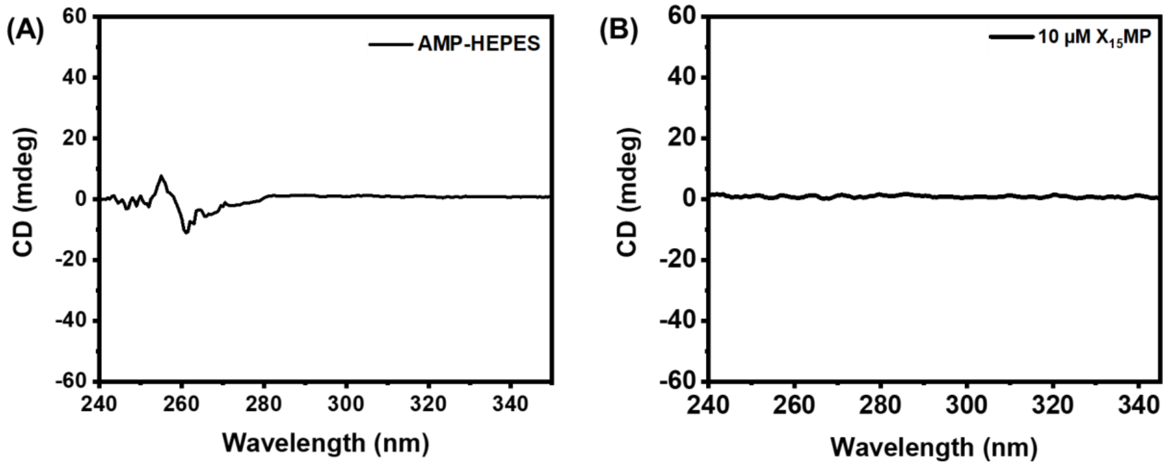

Figure S5. The CD spectrum of (**A**) pristine AMP and (**B**) $X_{15}MP$ (10 μM) in HEPES buffer. The result suggests that AMP and $X_{15}MP$ (below CAC) do not exhibit any CD signal under the test conditions.

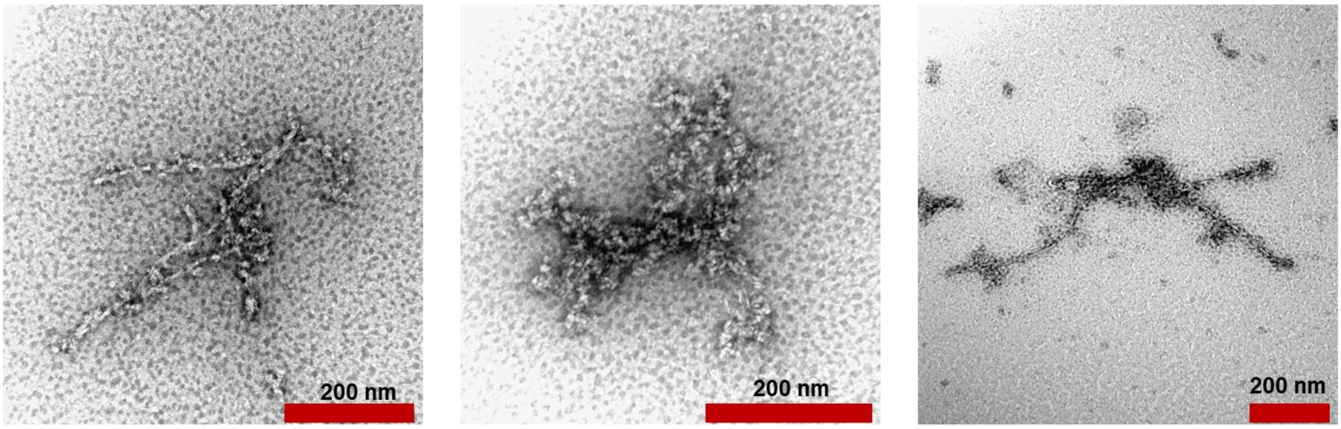

Figure S6. TEM images of $X_{15}MP$ (0.5 µmol, total concentration 0.5 mM, below CAC) in 50 µL H_2O, precipitated with 950 µL acetone (f_a = 95%) show a mixture of random aggregates and helical fibers.

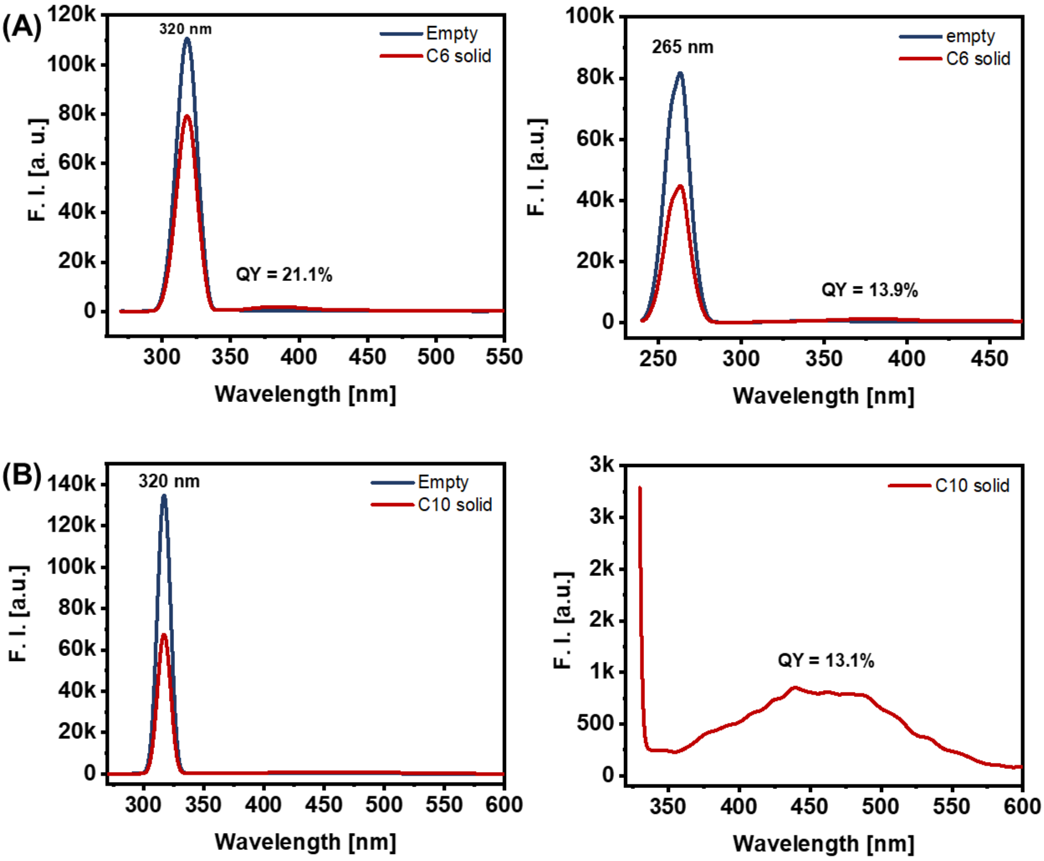

Figure S7. The fluorescence quantum yield (QY) of (**A**) C_6MP at 320 nm or 265 nm excitation wavelength and (**B**) $C_{10}MP$ in solid state was calculated around 21.1 %, 13.9% and 13.1%, respectively. The blue curve resembles the empty cuvette measurement and the red curve shows the spectrum of the C_nMP solids. The excitation intensity was calculated as the difference between the blue and the red curve area at the excitation wavelength. The emission intensity was obtained as the integral of the whole emission band between 350 and 550 nm. The QY was calculated as follows: $\Phi = \frac{\int excitation\ wavelength\ (empty) - \int excitation\ wavelength\ (CnMP)}{\int emission\ band\ (CnMP)}$.

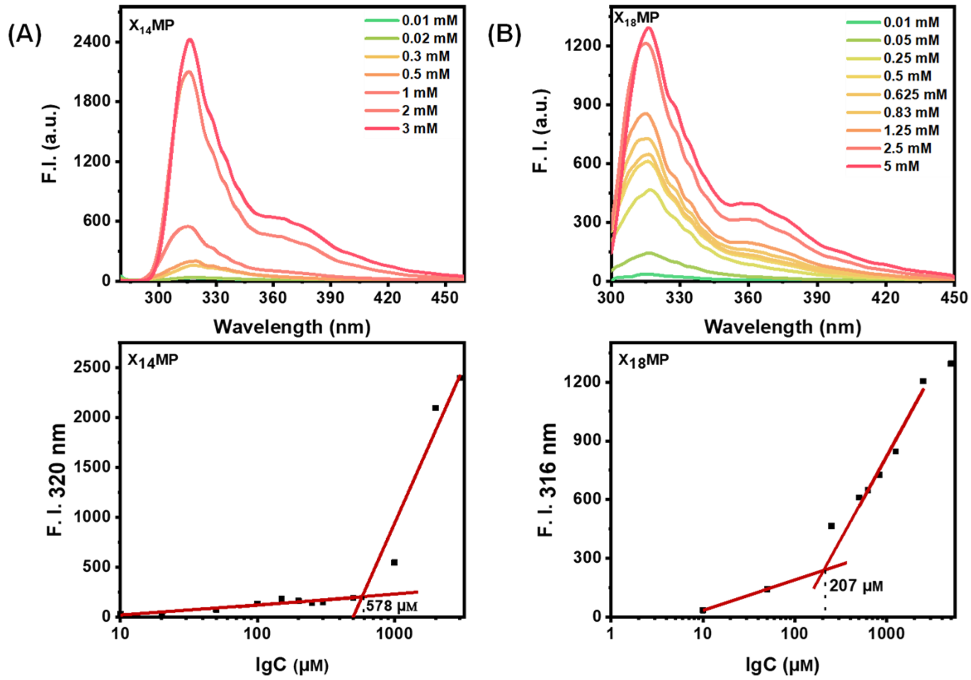

Figure S8. The concentration-dependent fluorescence behavior of (**A**) X_{14}MP and (**B**) X_{18}MP. The results show that the fluorescence intensity increases as the concentration of the monomer increases. In addition, the data can be used to determine the CAC of corresponding amphiphiles.

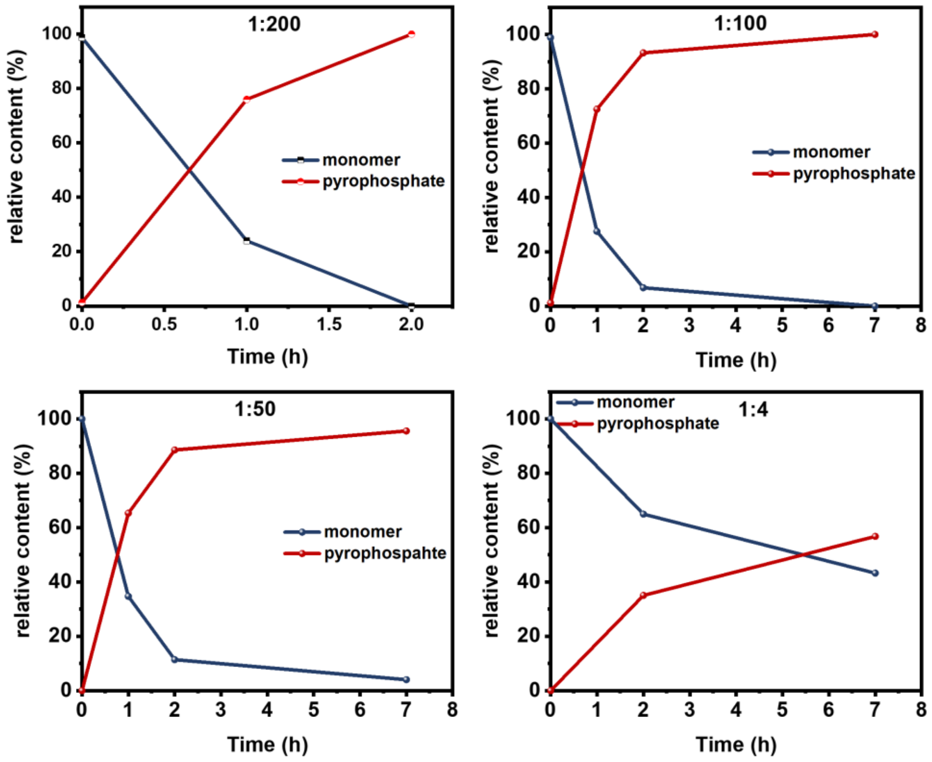

Figure S9. The molar ratio (X_{15}MP:EDC) effect on the EDC-triggered dimerization reaction of X_{15}MP was evaluated, including 1:200, 1:100, 1:50, and 1:4. X_{15}MP concentration was 500 μM in condensation buffer.

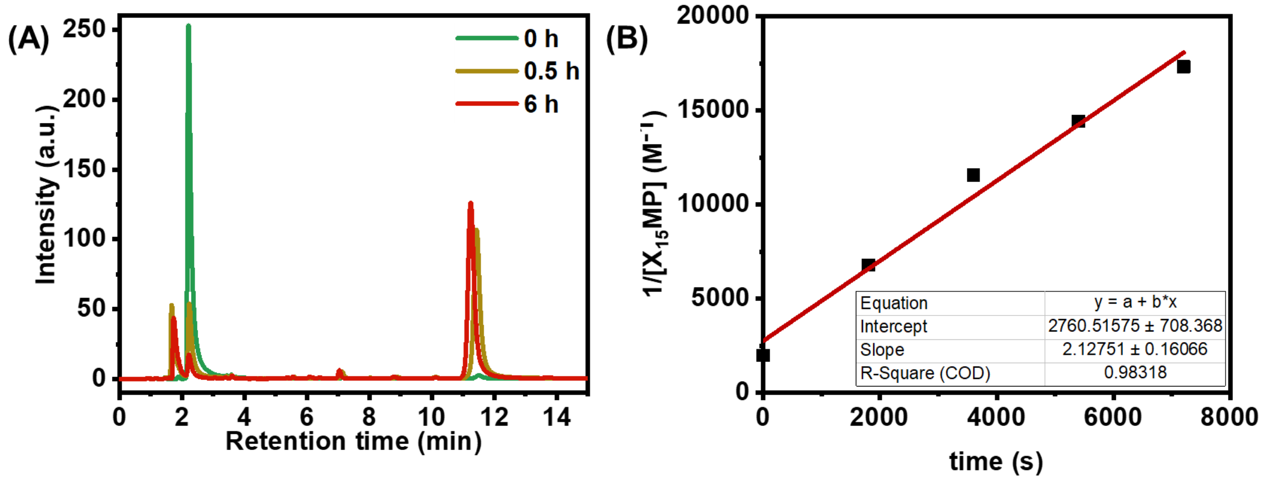

Figure S10. The reaction kinetics of the dimerization of X_{15}MP. (**A**) The HPLC chromatogram of the dimerization of X_{15}MP in the presence of EDC over 6 h. The X_{15}MP peak (2.2 min) decreases while the X15ppX15 (11.5 min) forms. (**B**) Experimental determination of the second-order rate constant of the reaction between X_{15}MP (0.5 mM) and EDC (25 mM), where $\frac{1}{[X15MP][M-1]}$ is plotted against the reaction time (s) and the slope equals the rate constant.

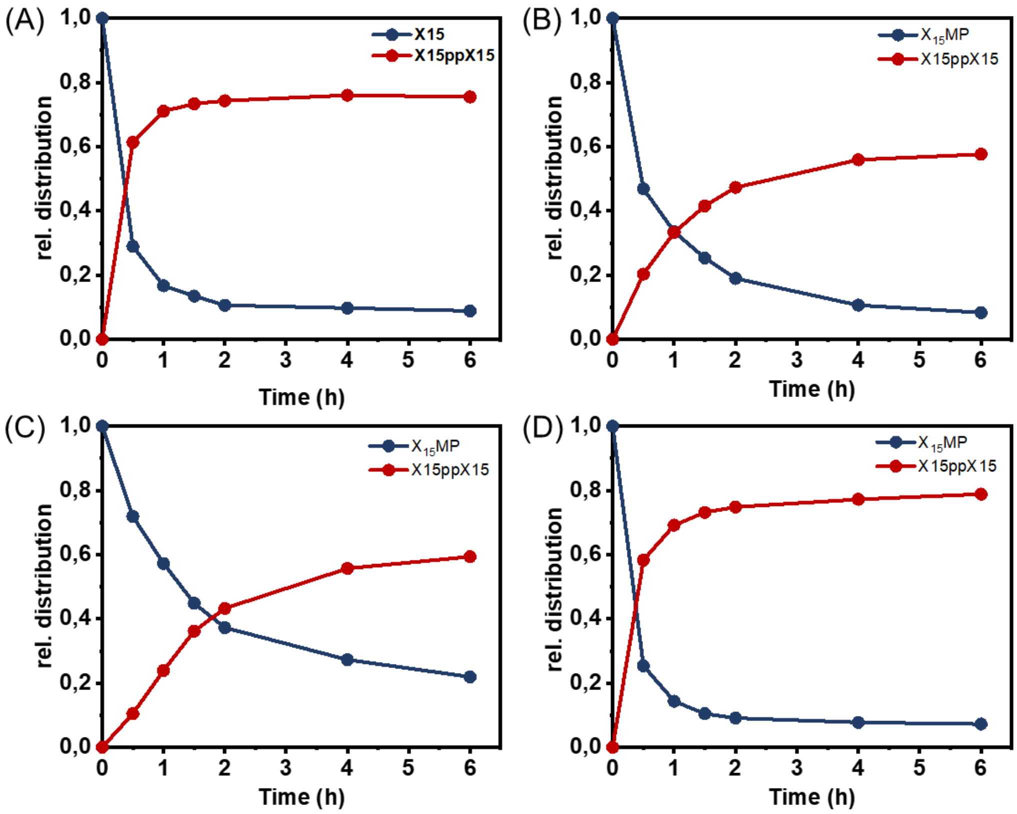

Figure S11. Further kinetic studies of the dimerization of **X_{15}MP** (0.5 mM, below CAC) in condensation buffer (**A**) at 25 °C (pH = 7.5) (these are the standard conditions), (**B**) at 10 °C (pH = 7.5), (**C**) at 0 °C (pH = 7.5) and (**D**) at pH = 6.0 (T = 25 °C), . respectively

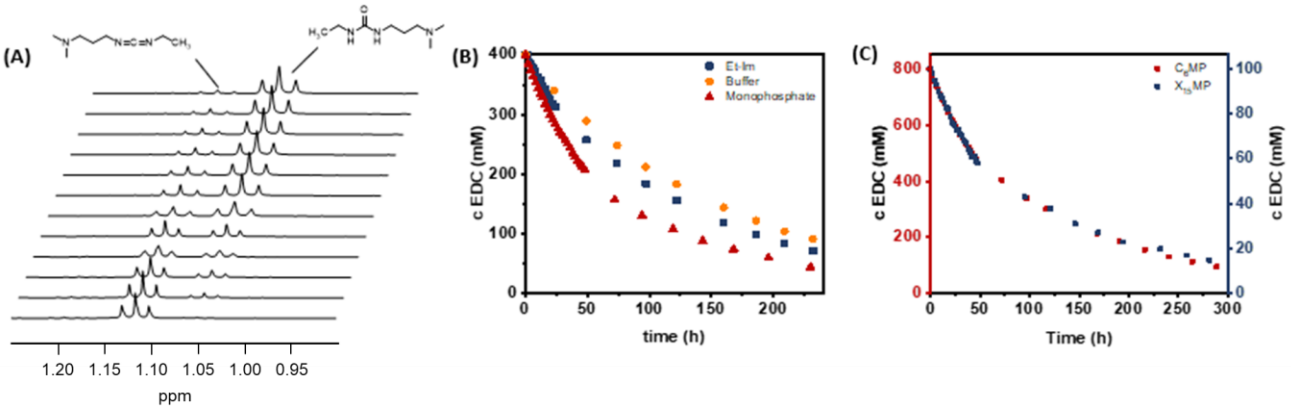

Figure S12. The hydrolysis kinetics of EDC. (**A**) The ^{1}H NMR shows how EDC is used up and EDU forms over time. (**B**) Comparison of three different conditions: Hydrolysis of EDC (0.4 M) in pure MOPS buffer (0.5 M MOPS, 0.08 M MgCl2, 10% D_2O) (orange), hydrolysis in the buffer containing 1-ethylimidazole (0.5 M MOPS, 0.15 M EtIm, 0.08 M $MgCl_2$) (blue), hydrolysis due to monophosphate activation (5 mM C_6MP) (red). The data show that a major part of the EDC in the system is consumed by the activation of the monophosphate. (**C**) Comparison of consumption of EDC (160 eq. compared to the monophosphates, respectively) in a system with different monophosphates, C_6MP (red) and X_{15}MP (blue). The activation chemistry of different monophosphates is similar.

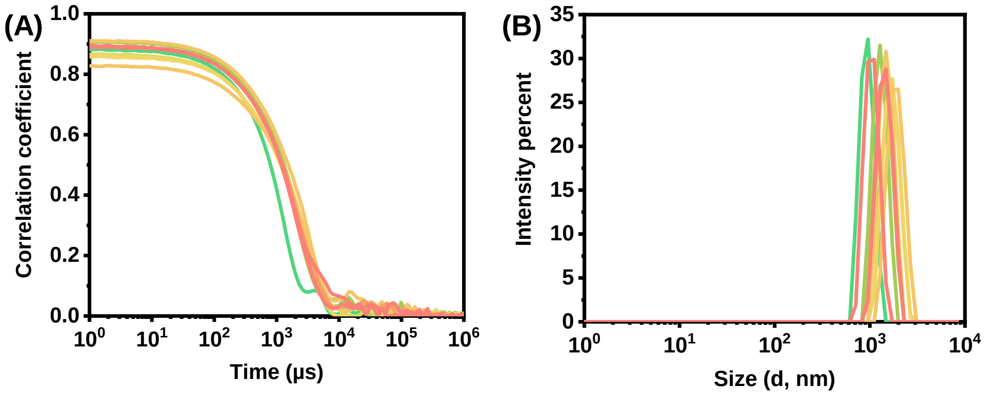

Figure S13. Characterization of isolated X15ppX15 (200 μM) by DLS. (**A**) Correlograms (**B**) Size distribution. The results indicated that X15ppX15 formed large aggregates when the concentration of X15ppX15 was above its CAC.

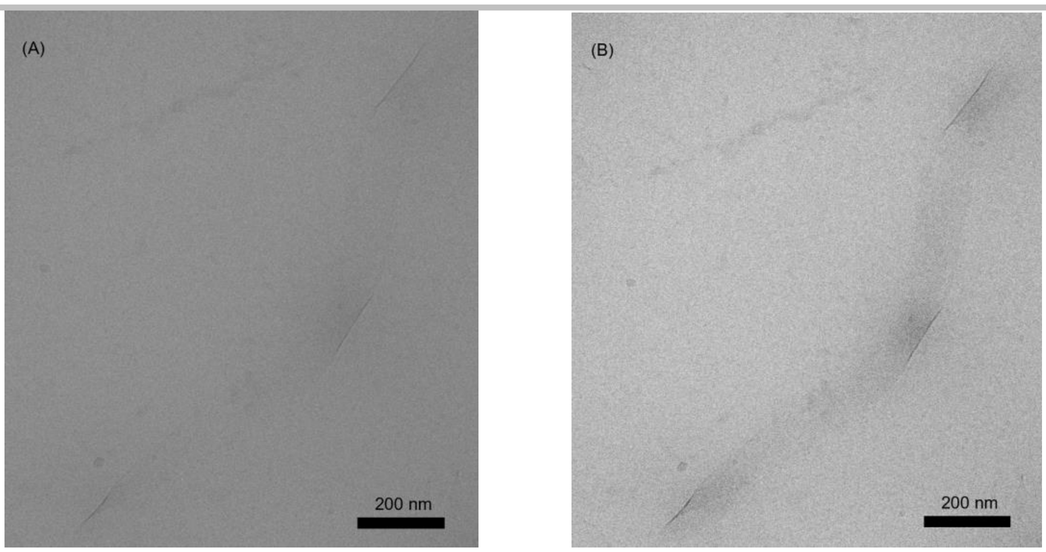

Figure S14. Cryo-TEM of the X15MP dimerization. A 500 μM (below CAC) solution of X15MP in condensation buffer was dimerized with 50 eq. EDC and cryo-TEM was measured after 2 h. (**A**) Original image and (**B**) image after enhancing the contrast with the softwares ImageJ and Adobe Photoshop. The formation of helical aggregates in solution is confirmed.

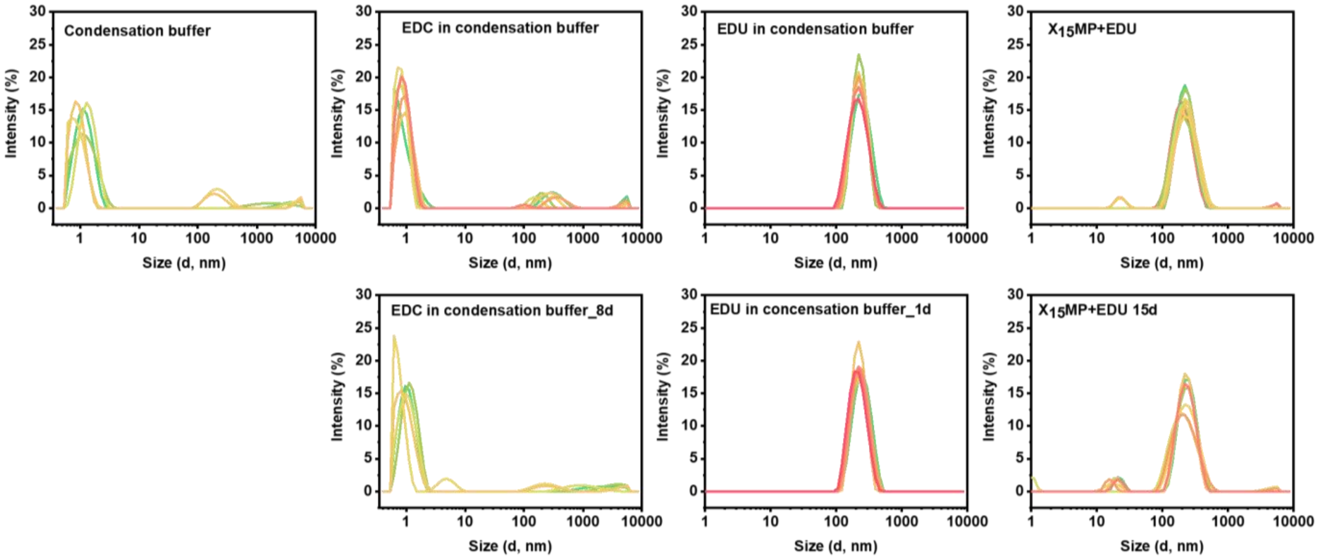

Figure S15. The control DLS experiments, including (i) condensation buffer, (ii) EDC in condensation buffer over different times, (iii) EDU in condensation buffer over different times, (iv) X15MP+EDC in condensation buffer over different time. All the results indicate that the control experiments do not form large aggregates.

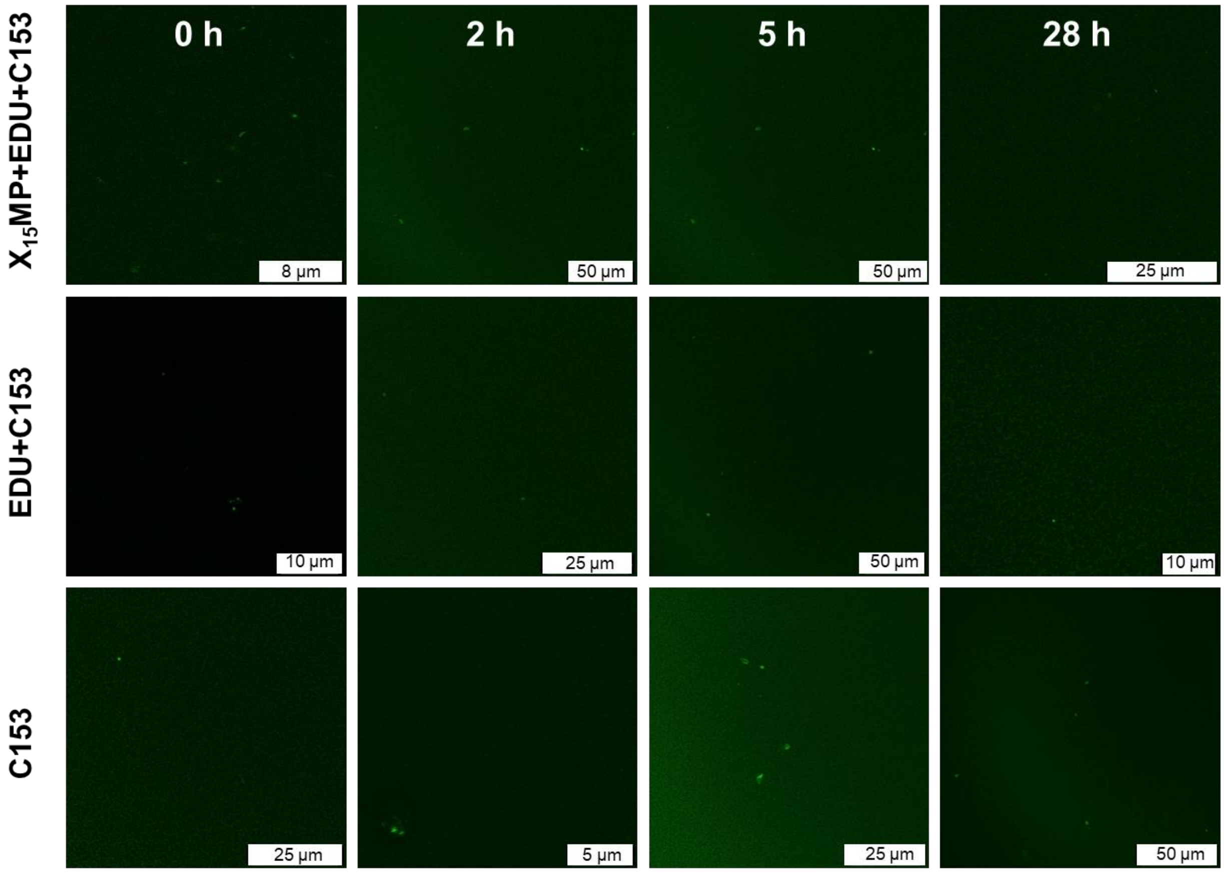

Figure S16. The control CLSM experiments, including (i) X_{15}MP+EDU+C153, (ii) EDU+C153, (iii) C153. The CLSM images over time indicate that there is no aggregation formed under these control conditions. (C153: Coumarin-153 dye).

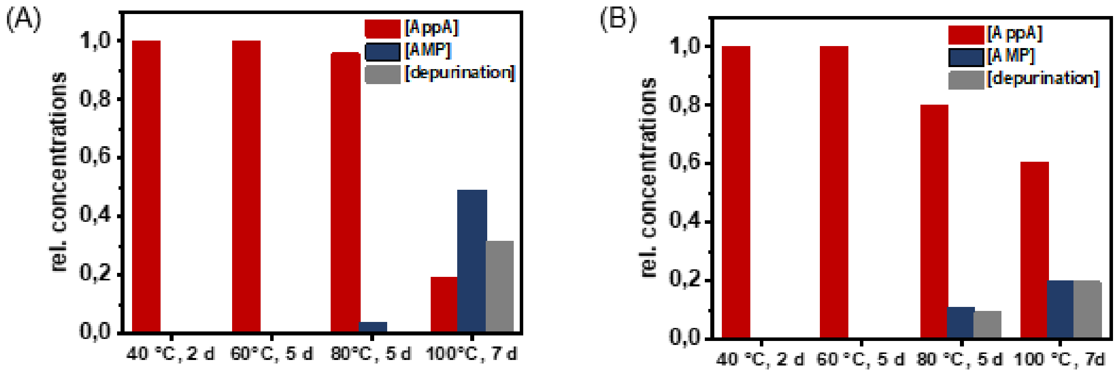

Figure S17. Additional data on the hydrolysis of AppA under (**A**) neutral and (**B**) alkaline (0.02 M NaOH) conditions.

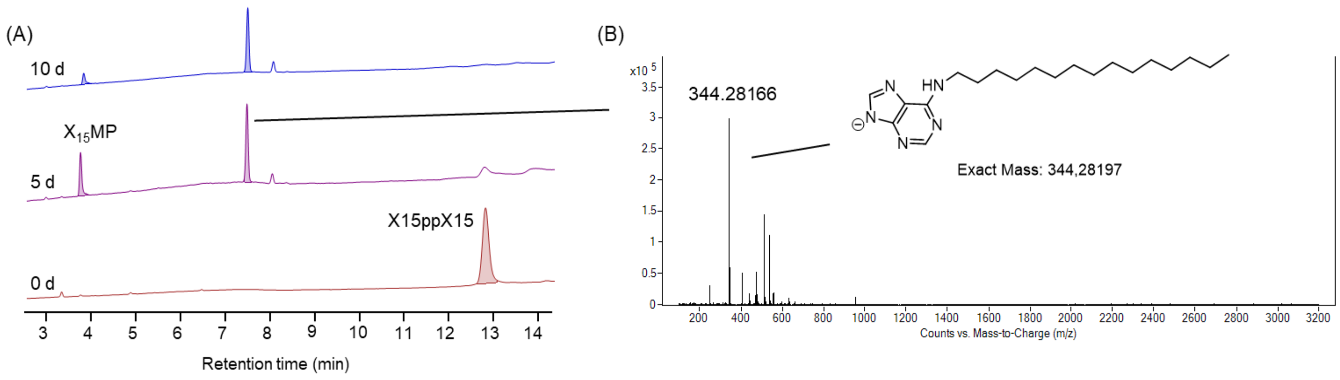

Figure S18. Hydrolysis of X15ppX15 monitored by HPLC-MS. (**A**) Stacked chromatograms after 0, 5 and 10 days. (**B**) Mass spectrum of the depurination product. A 1 mM solution of X15ppX15 in aqueous solution containing 4 eq. $EuCl_3$ (pH ≈ 6) was stirred at 60 °C. HPLC was performed on an Agilent Infinity Lab Poroshell 120 EC-C18 column (3.0 x 100mm, 2.7 µm).

Table S1. The hydrolysis studies of X15ppX15 under different conditions, including pH, lanthanide salt, temperature, and enzyme.

	Conditions	Product
	MES buffer, pH 5.87, 25°C	pyrophosphate
	MES buffer, pH 5.87, 37°C	pyrophosphate
	MES buffer+100µM Tb^{3+}, pH 6.04, 25°C	pyrophosphate
X15:EDC = 1:50	MES buffer+100µM Tb^{3+}, pH 6.04, 37°C	pyrophosphate
	MES buffer+100µM Eu^{3+}, pH 5.92, 25°C	pyrophosphate
	MES buffer+100µM Eu^{3+}, pH 5.92, 37°C	pyrophosphate
	HEPES buffer, pH 7.46+Enzyme, 37°C	pyrophosphate
X15ppX15	HEPES buffer, pH 7.46+Enzyme, 37°C	pyrophosphate

NMR spectra

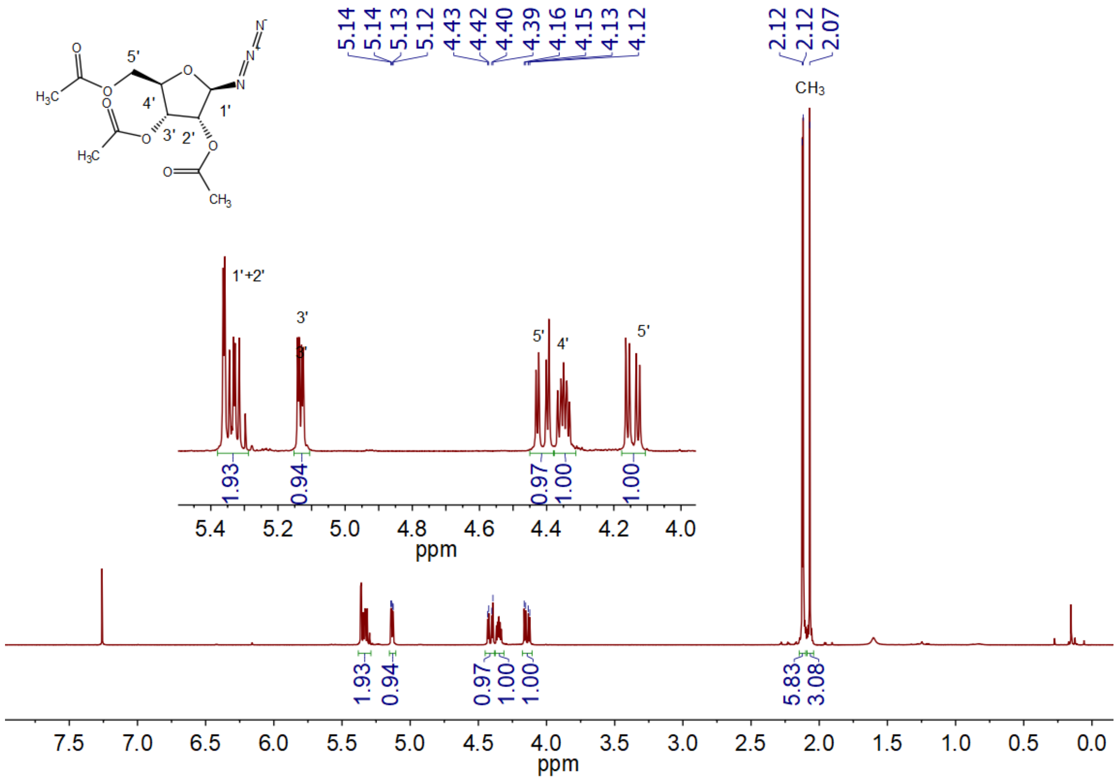

Figure S19. ^{1}H NMR spectrum of compound **2** ($CDCl_3$, 400 MHz, 295 K).

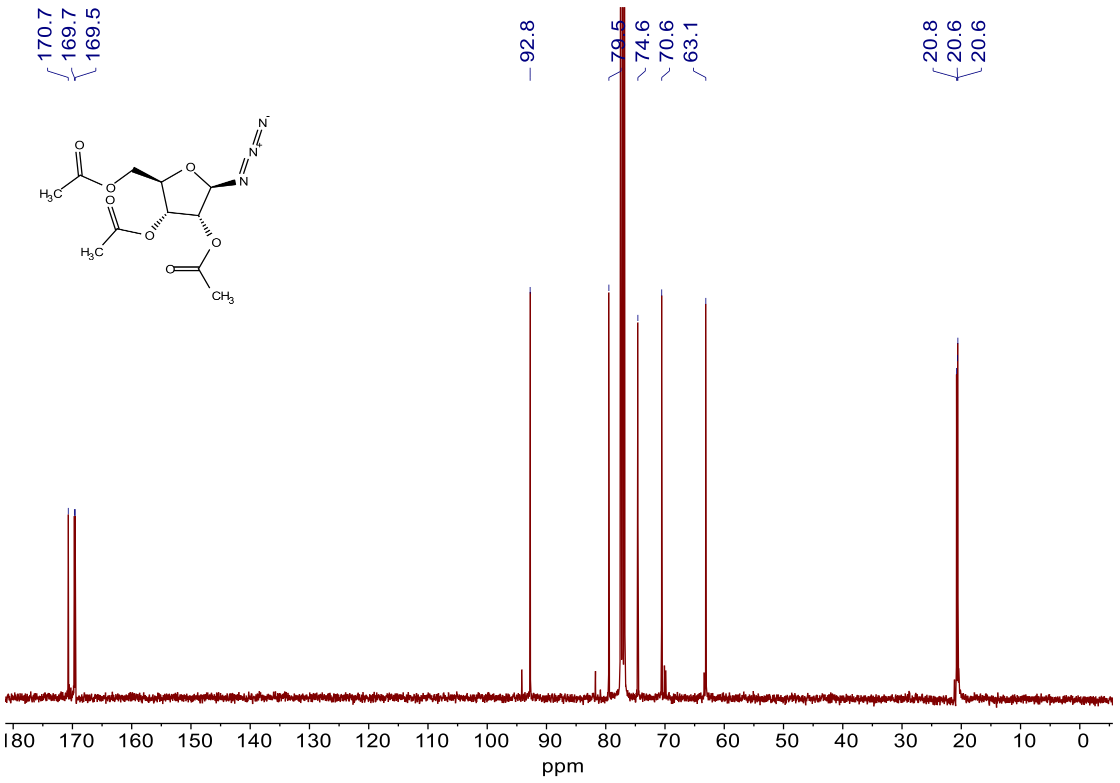

Figure S20. ^{13}C NMR spectrum of compound **2** ($CDCl_3$, 101 MHz, 295 K).

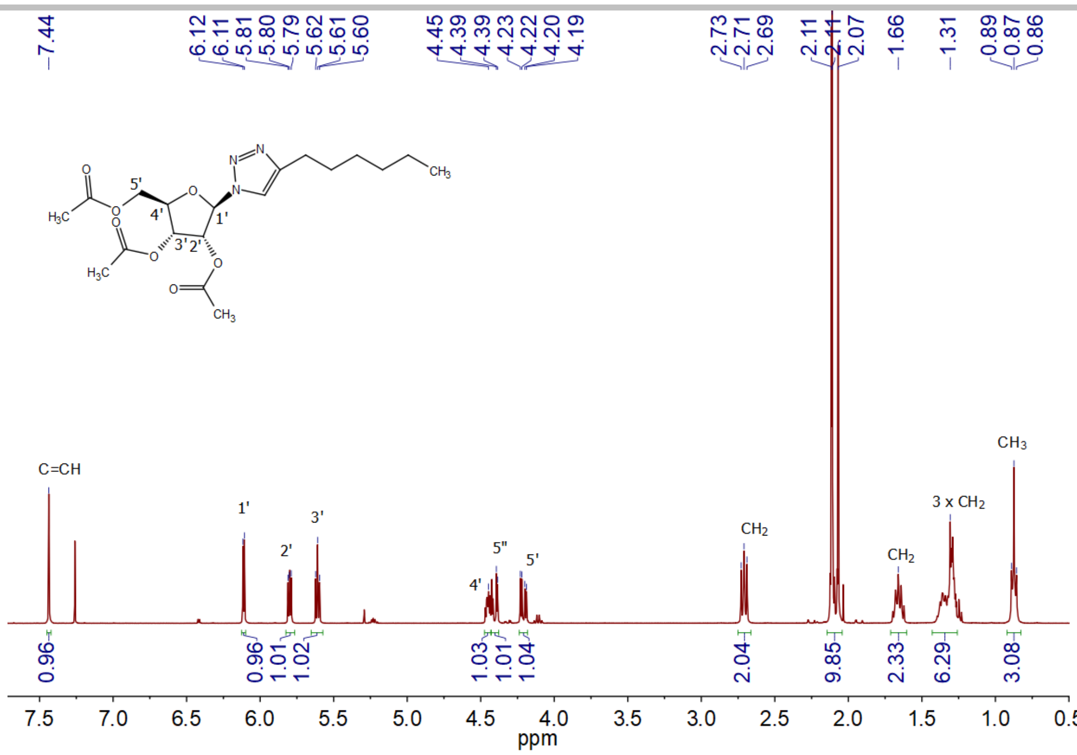

Figure S21. ^{1}H NMR spectrum of compound **3** ($CDCl_3$, 400 MHz, 295 K).

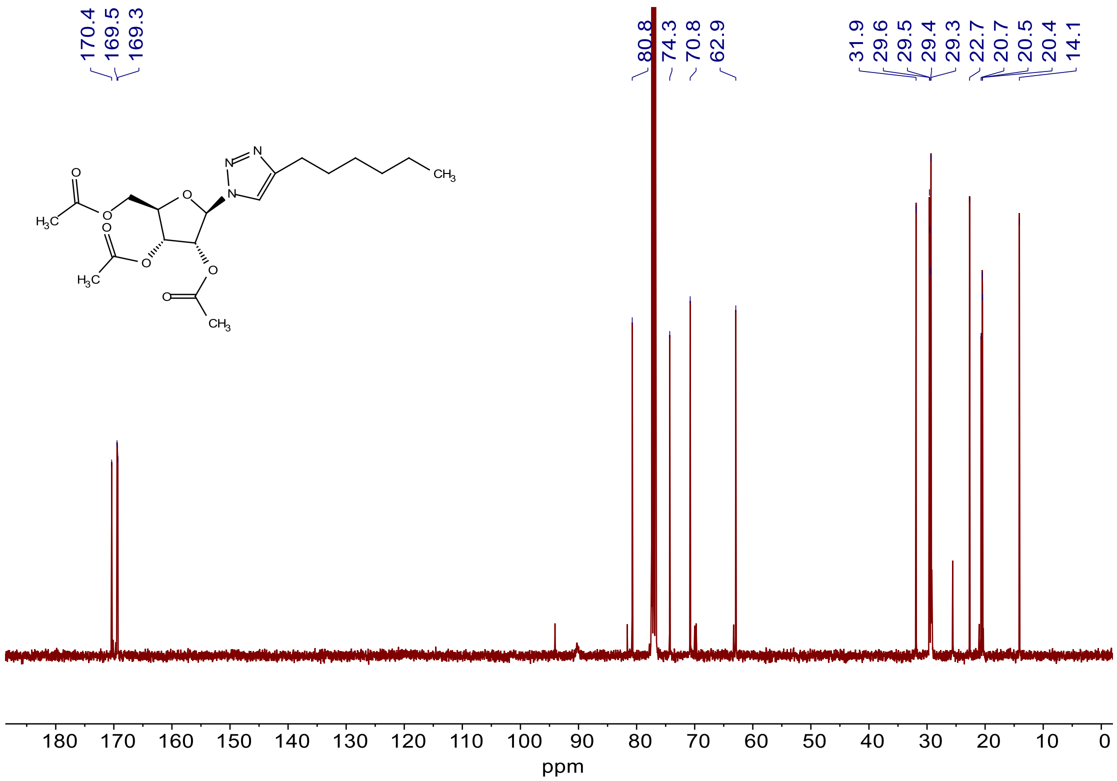

Figure S22. ^{13}C NMR spectrum of compound **3** ($CDCl_3$, 101 MHz, 295 K).

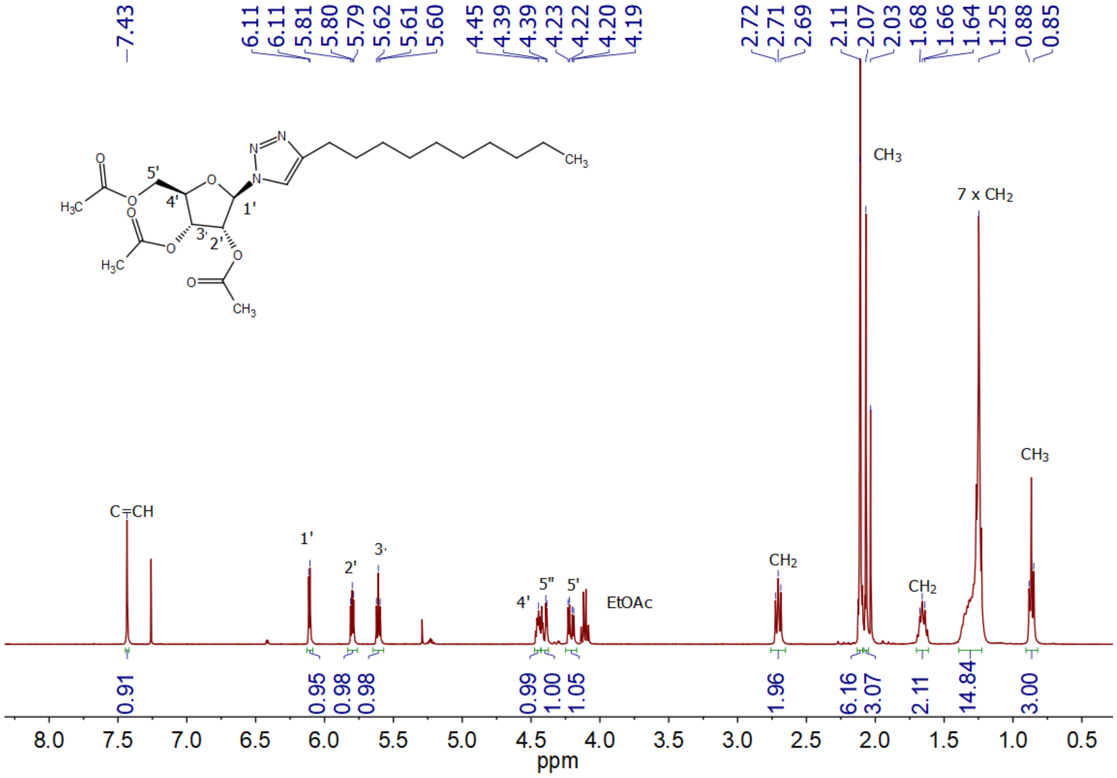

Figure S23. ^{1}H NMR spectrum of compound **4** ($CDCl_3$, 400 MHz, 295 K).

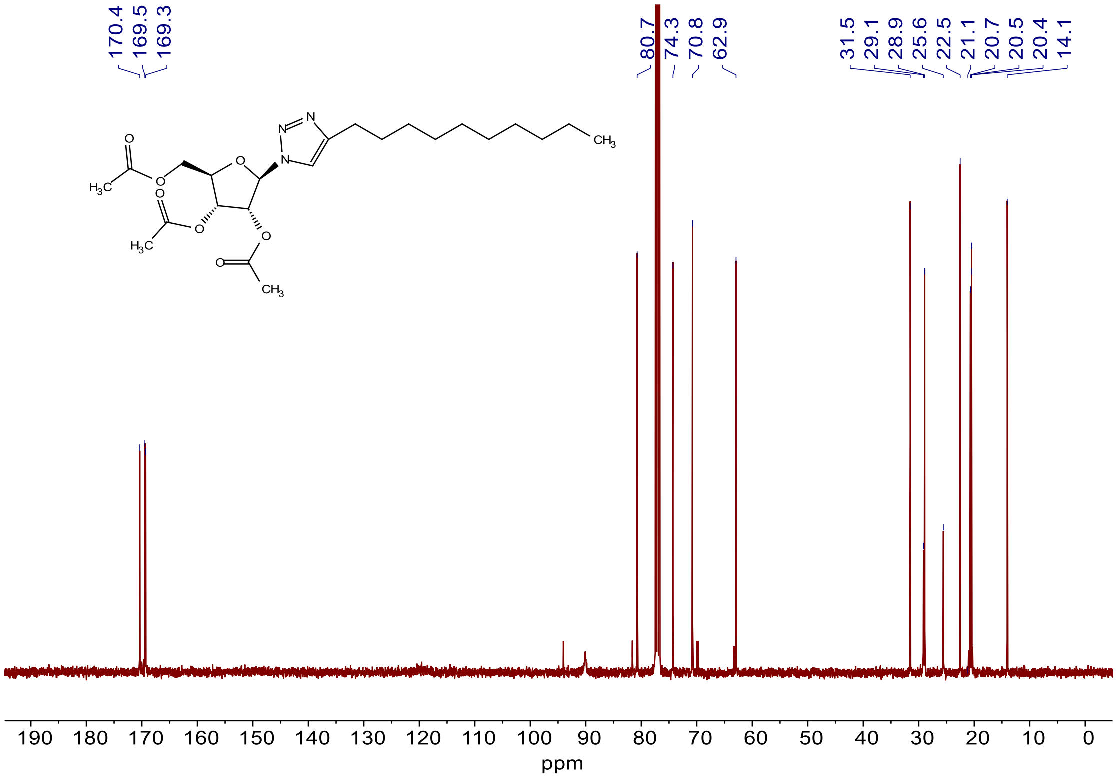

Figure S24. ^{13}C NMR spectrum of compound **4** ($CDCl_3$, 101 MHz, 295 K).

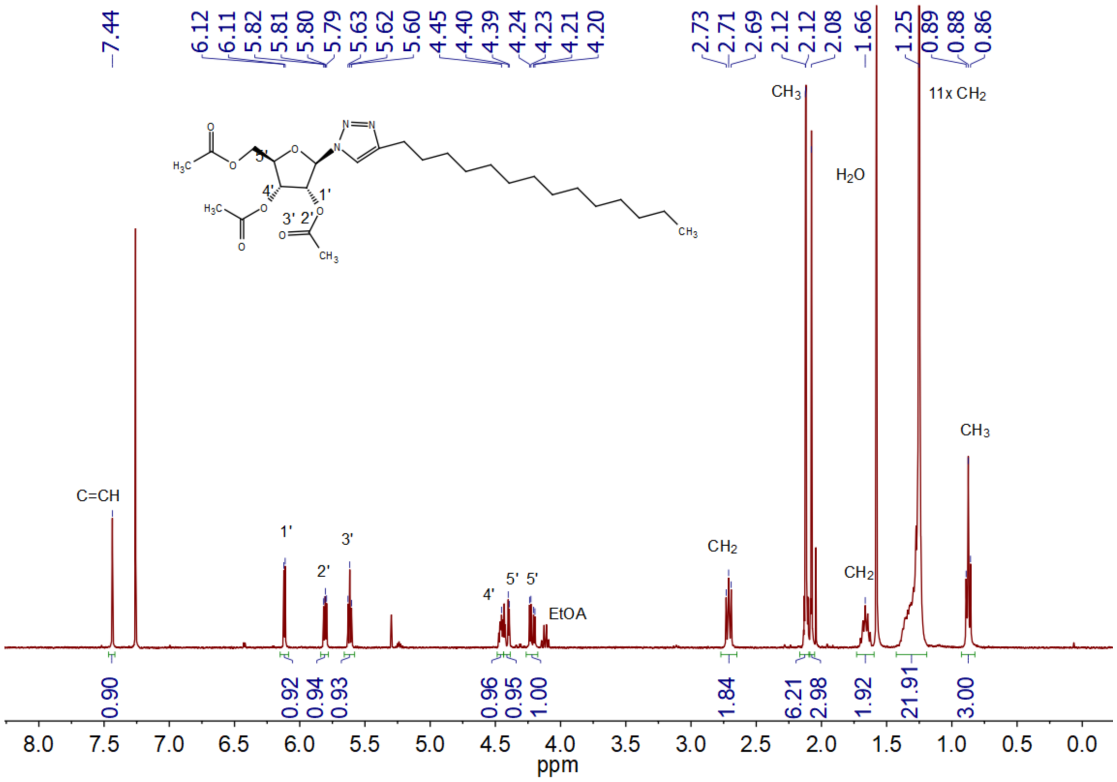

Figure S25. ^{1}H NMR spectrum of compound **5** ($CDCl_3$, 400 MHz, 295 K).

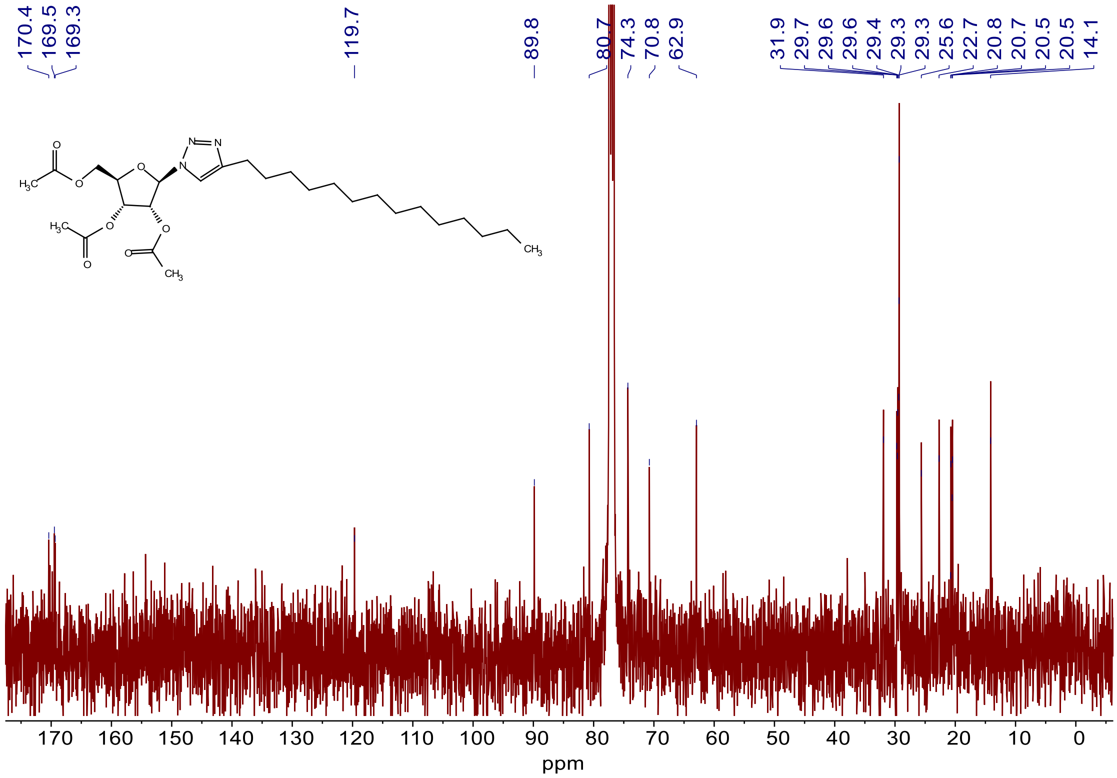

Figure S26. ^{13}C NMR spectrum of compound **5** ($CDCl_3$, 101 MHz, 295 K).

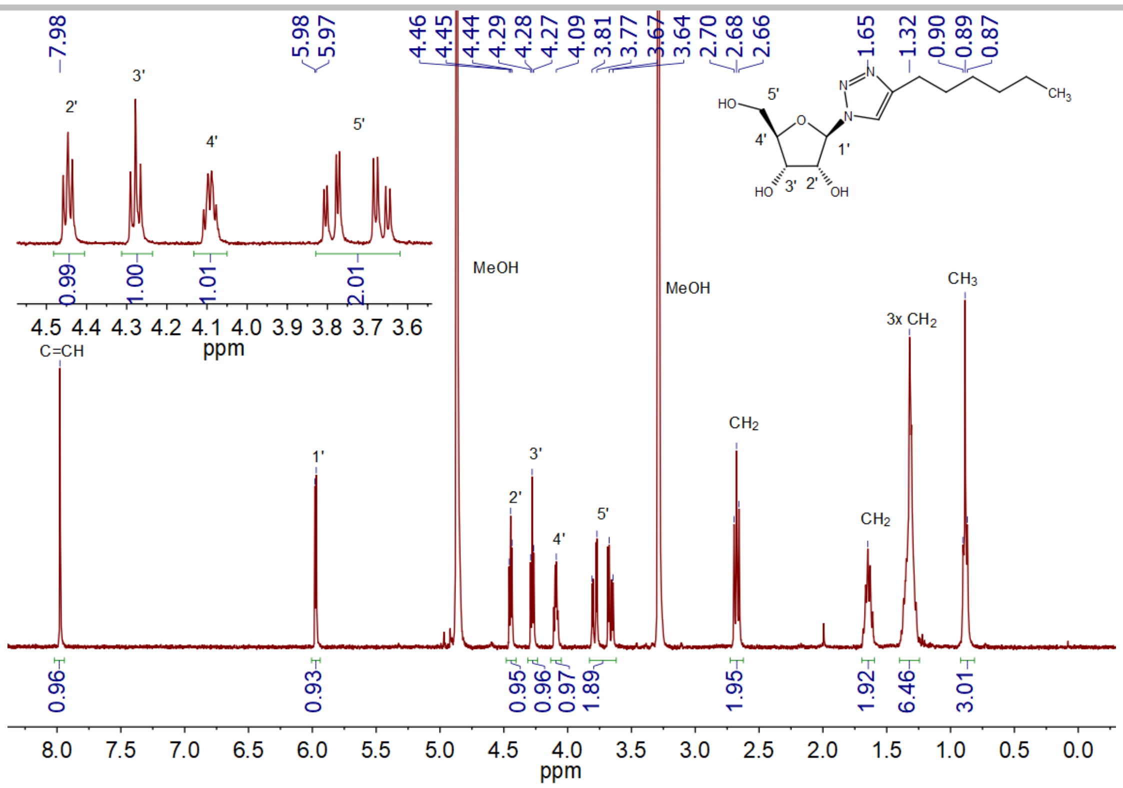

Figure S27. ^{1}H NMR spectrum of compound **6** ($CDCl_3$, 400 MHz, 295 K).

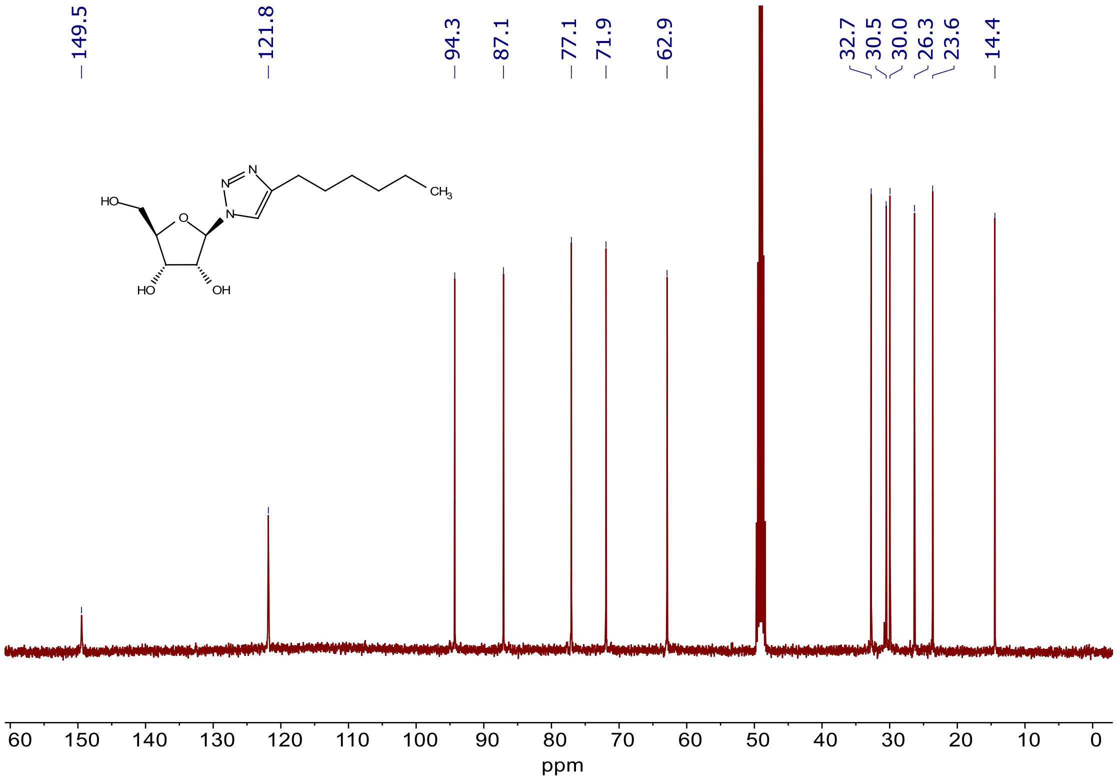

Figure S28. ^{13}C NMR spectrum of compound **6** ($CDCl_3$, 101 MHz, 295 K).

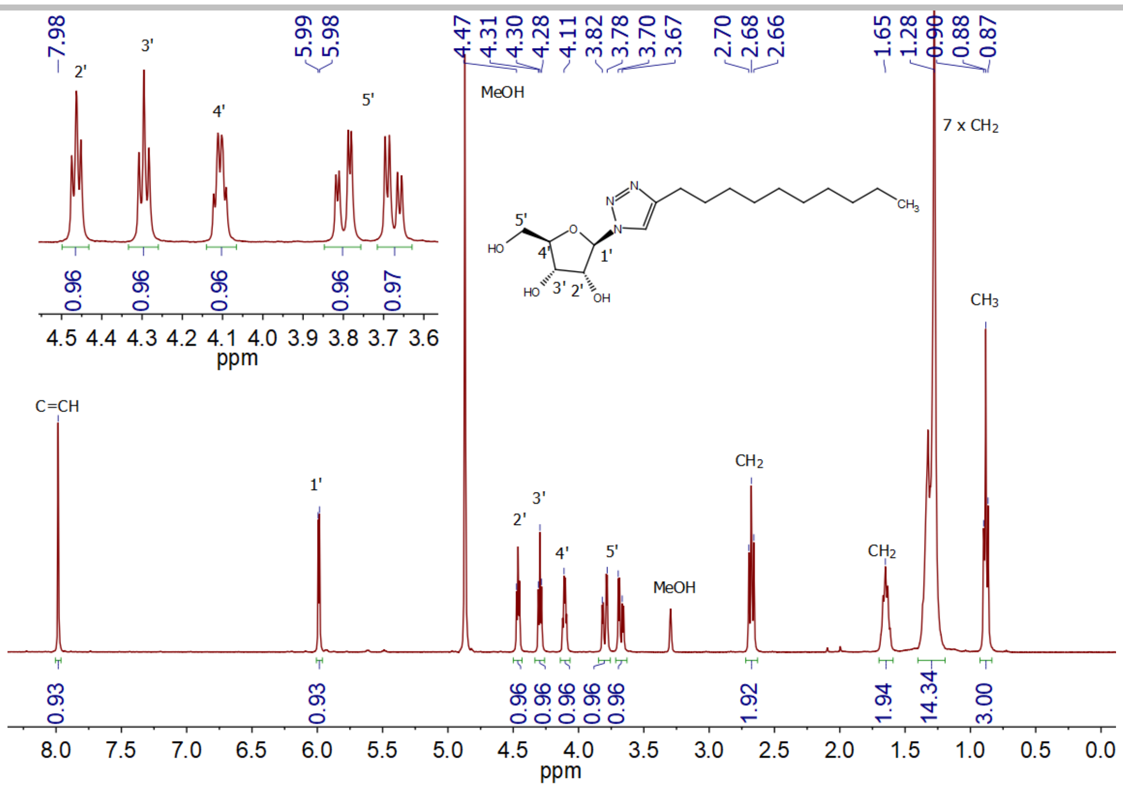

Figure S29. ^{1}H NMR spectrum of compound **7** ($CDCl_3$, 400 MHz, 295 K).

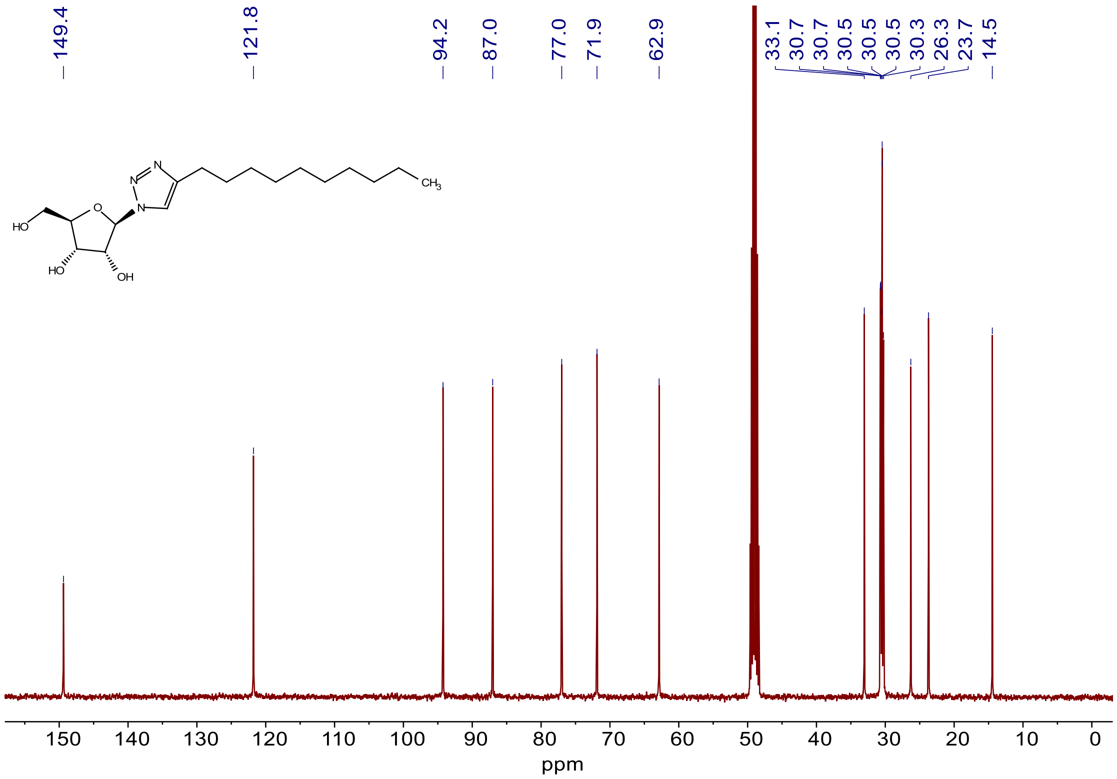

Figure S30. ^{13}C NMR spectrum of compound **7** ($CDCl_3$, 101 MHz, 295 K).

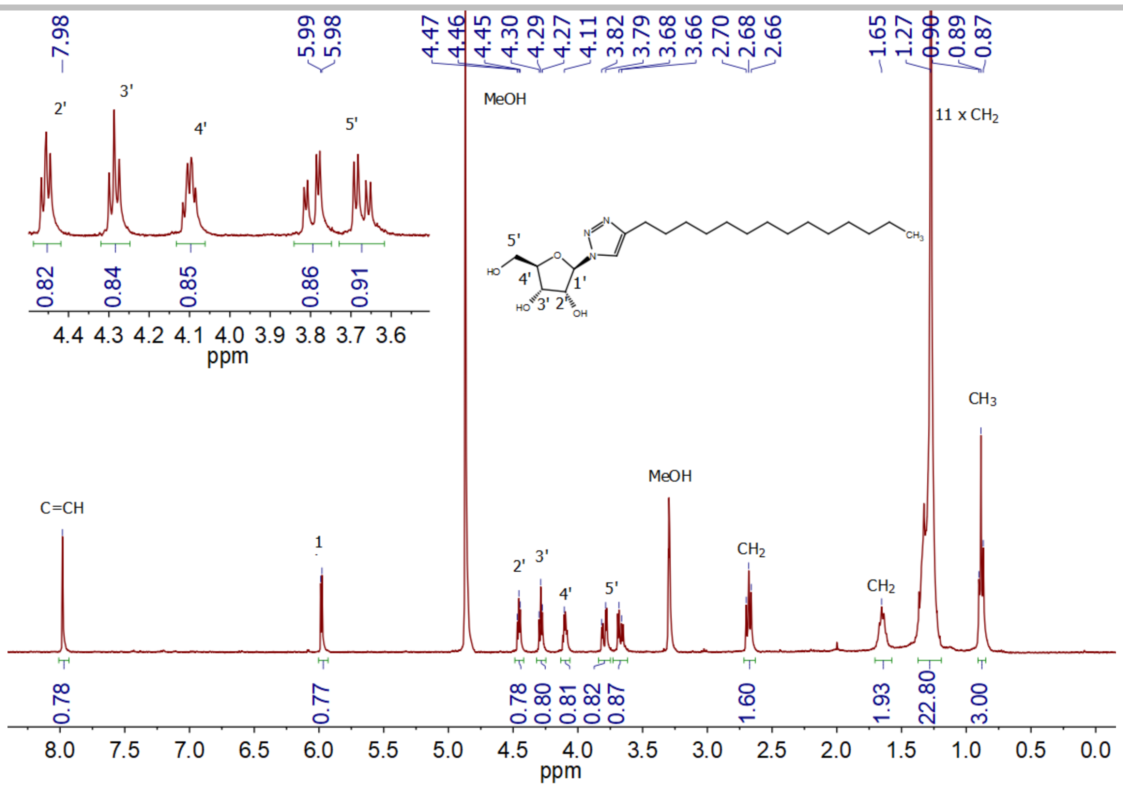

Figure S31. ^{1}H NMR spectrum of compound **8** ($CDCl_3$, 400 MHz, 295 K).

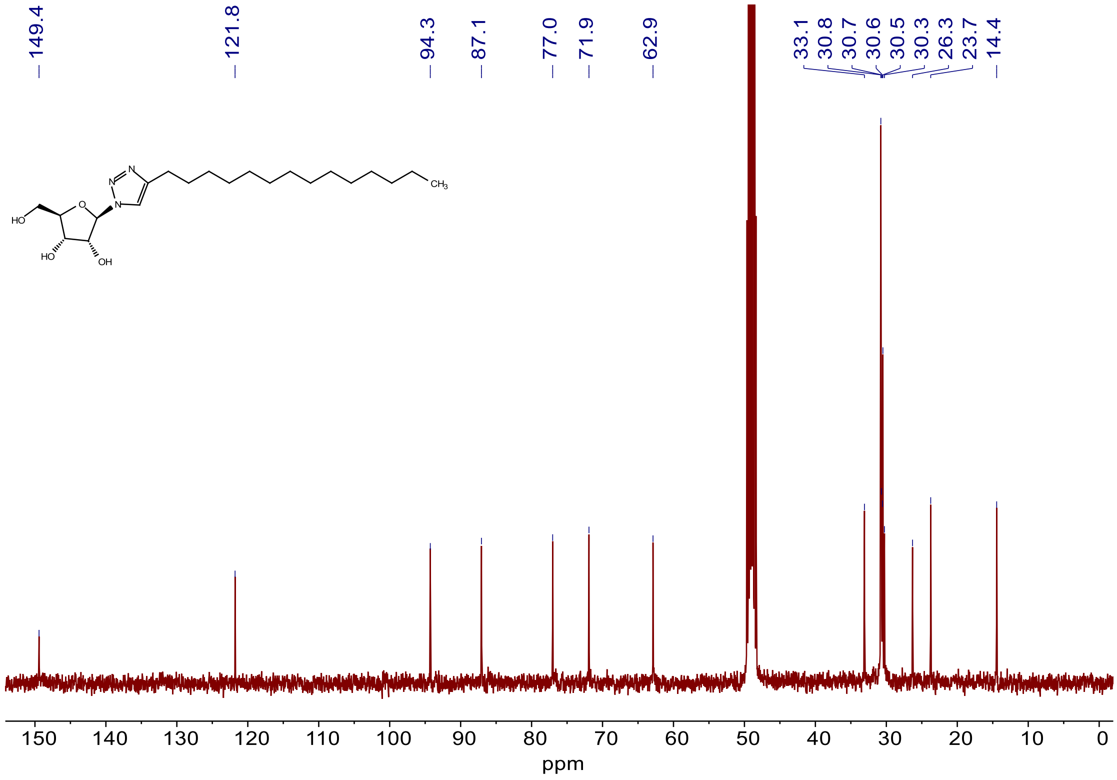

Figure S32. ^{13}C NMR spectrum of compound **8** ($CDCl_3$, 101 MHz, 295 K).

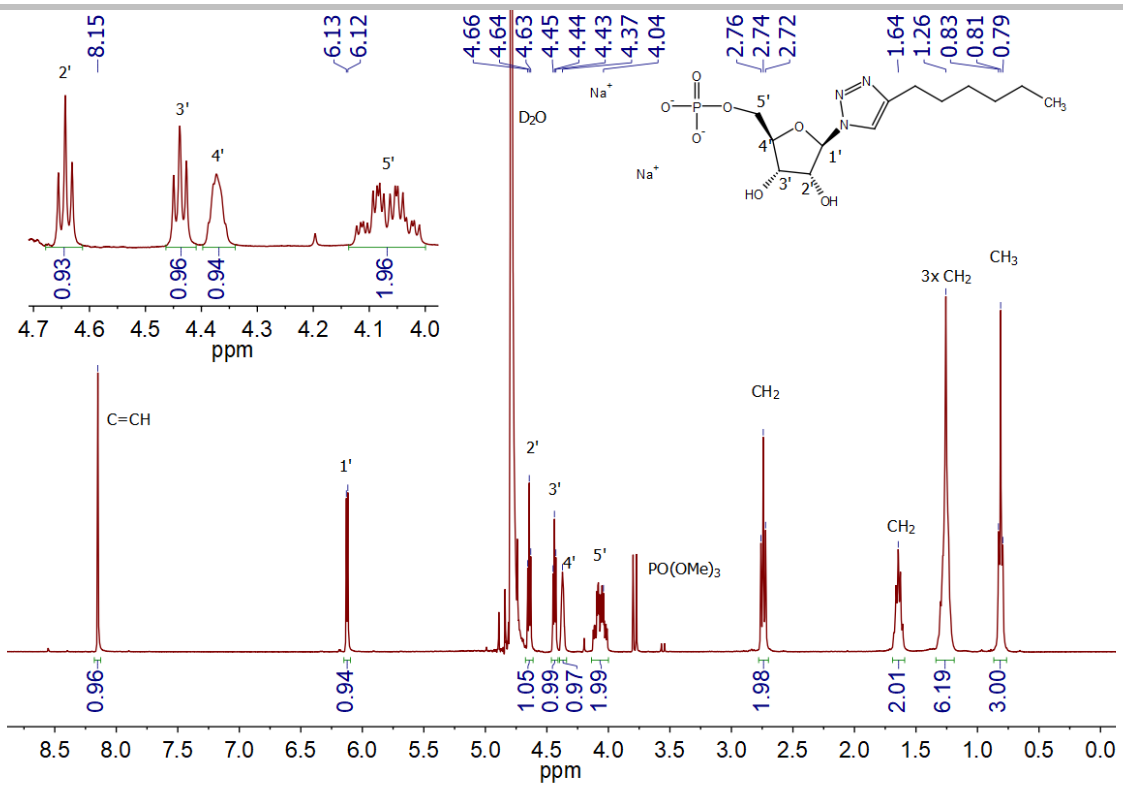

Figure S33. ^{1}H NMR spectrum of compound **9** ($CDCl_3$, 400 MHz, 295 K).

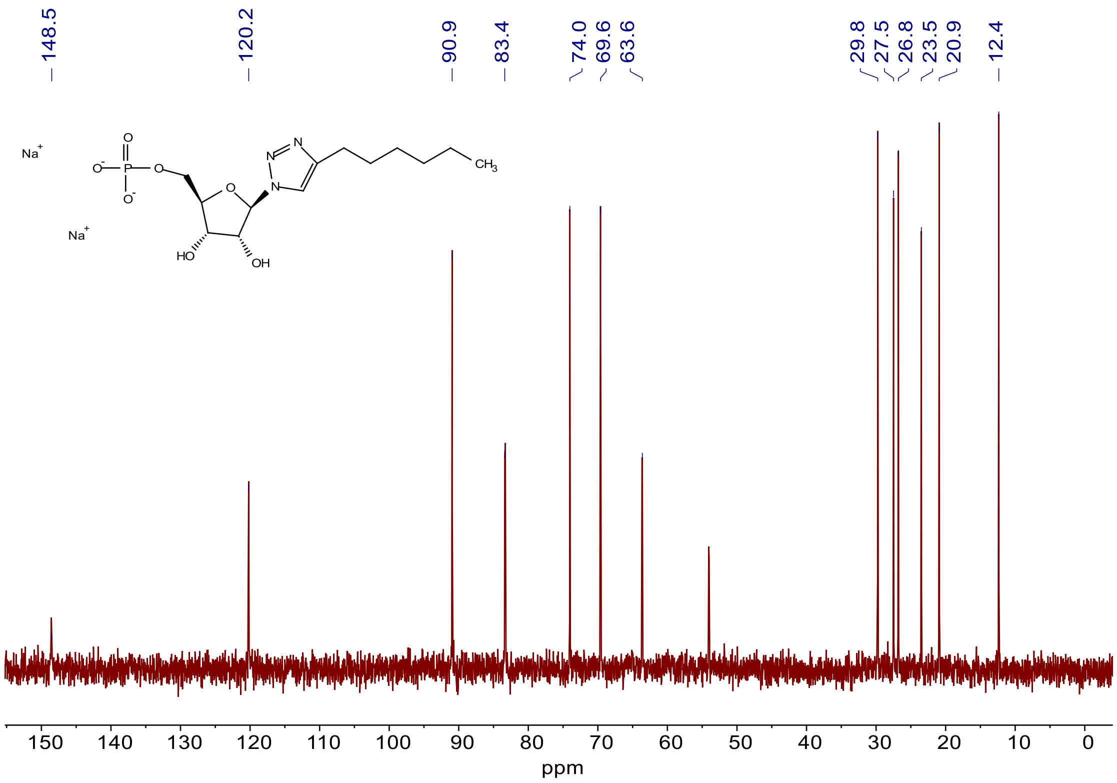

Figure S34. ^{13}C NMR spectrum of compound **9** ($CDCl_3$, 101 MHz, 295 K).

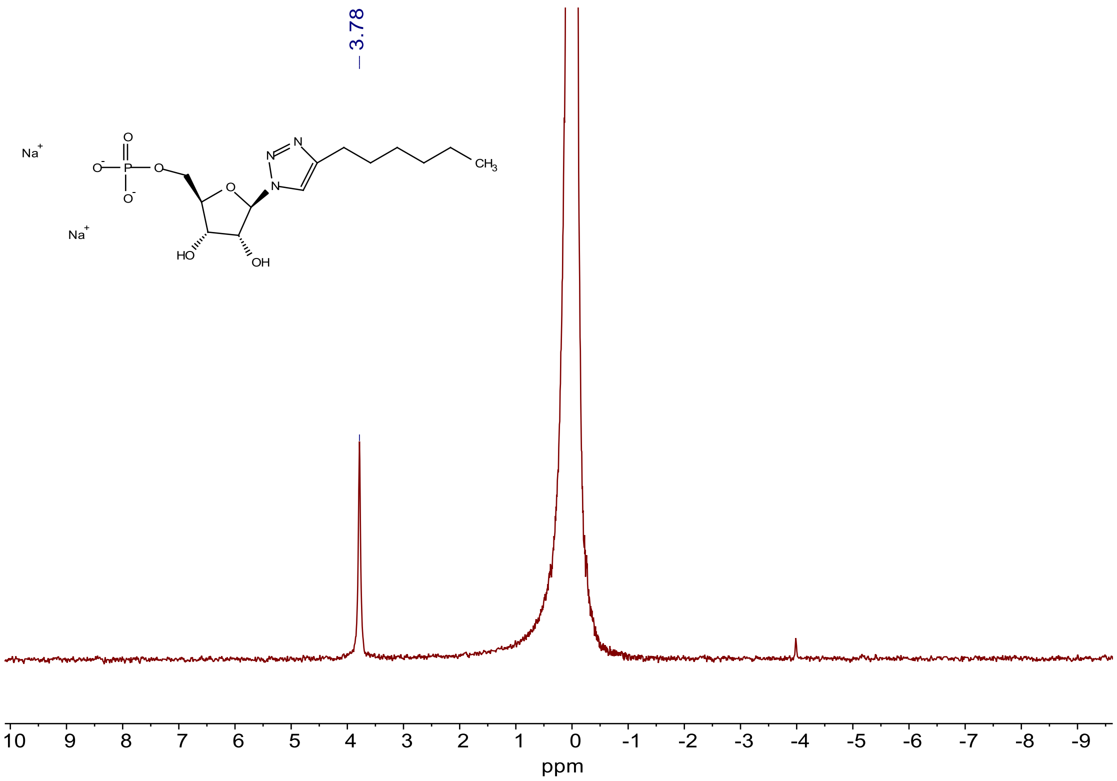

Figure S35. ^{31}P NMR spectrum of compound **9** (HEPES buffer pH = 7.50 (10% D_2O), against 85% H_3PO_4, 162 MHz, 295 K)

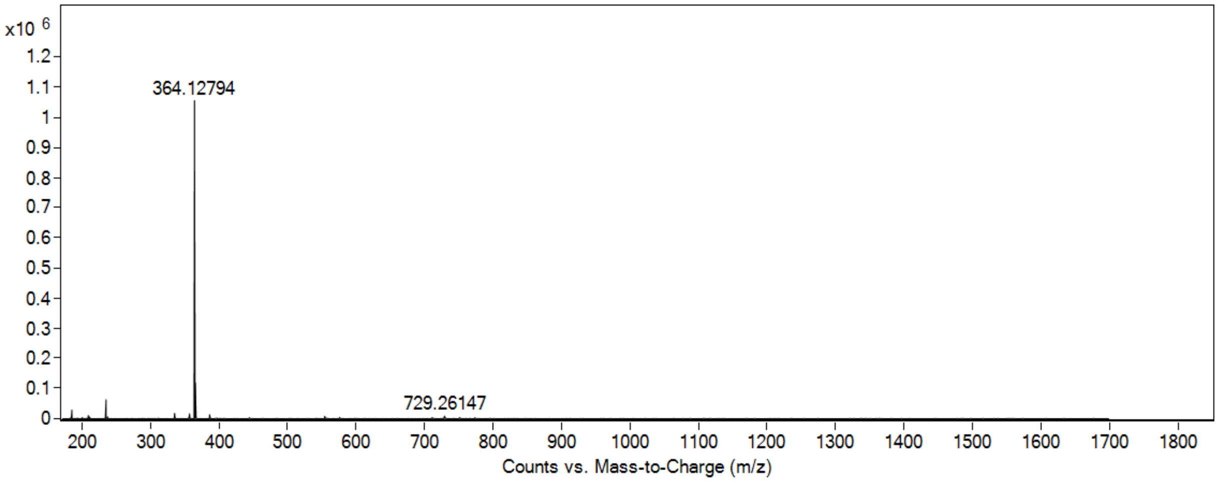

Figure S36. HR-MS (-) of compound **9**.

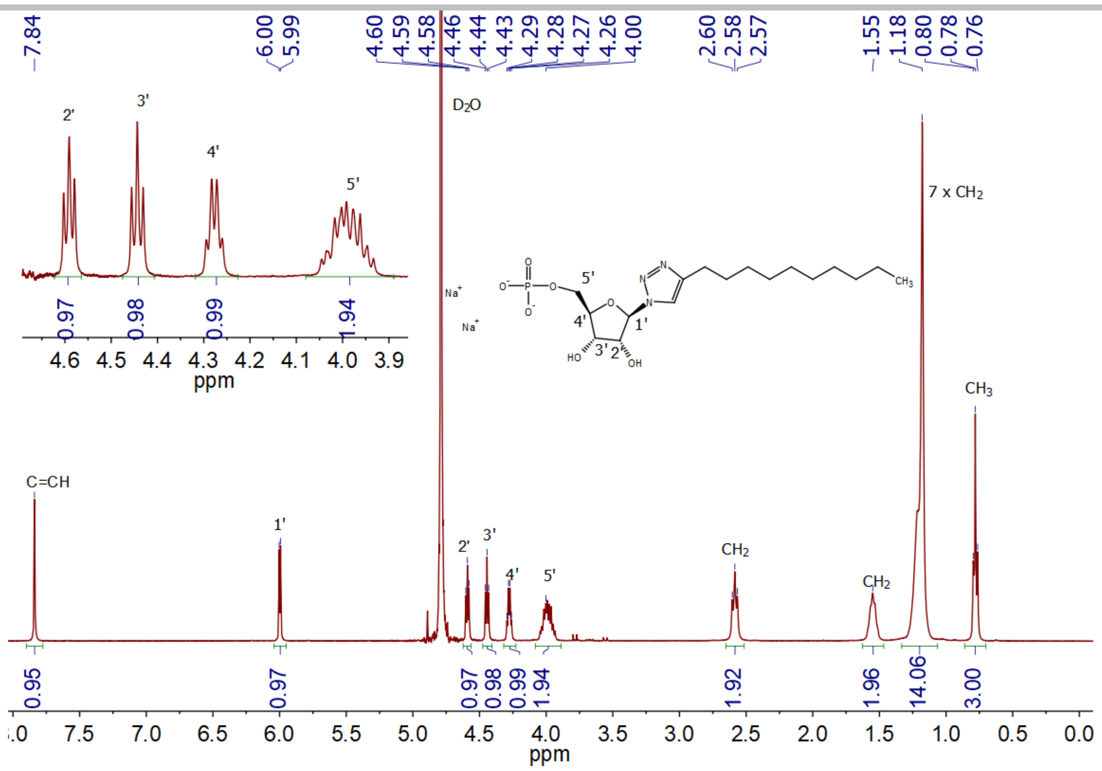

Figure S37. ^{1}H NMR spectrum of compound **10** (D_2O, 400 MHz, 295 K).

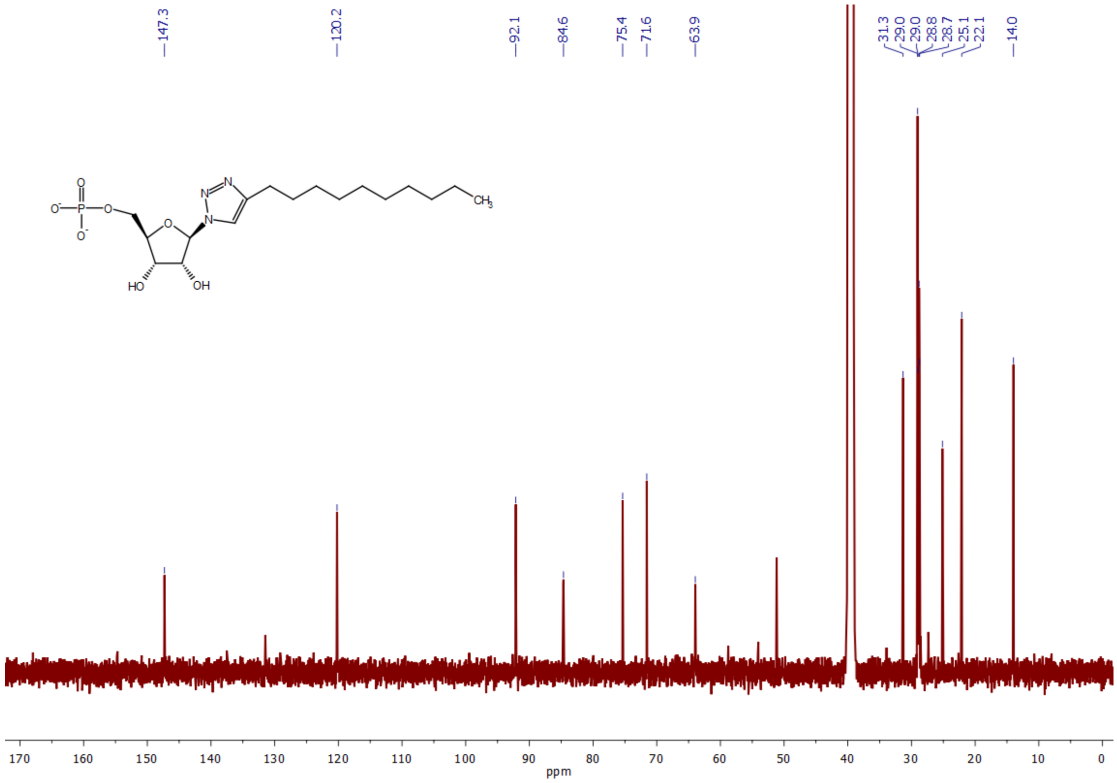

Figure S38. ^{13}C NMR spectrum of compound **10** (DMSO-d_6, 101 MHz, 295 K).

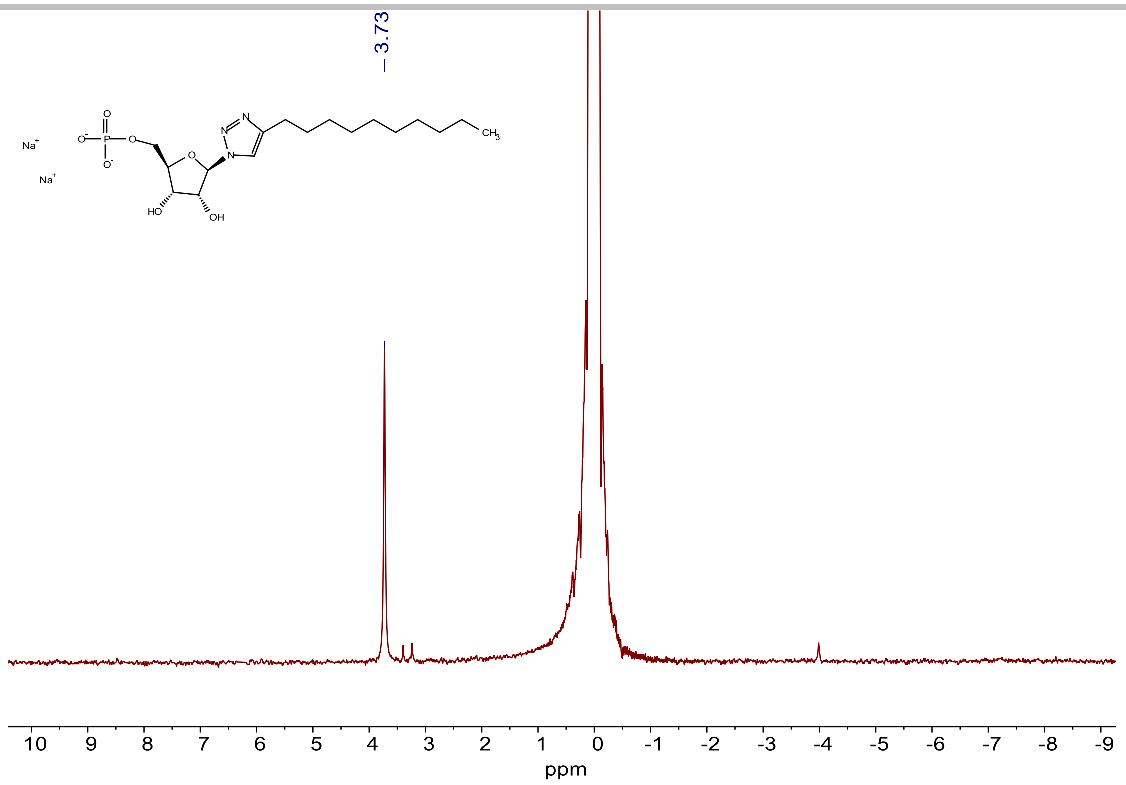

Figure S39. **^{31}P NMR** spectrum of compound **10** (HEPES buffer pH = 7.50 (10% D_2O), against 85% H3PO4, 162 MHz).

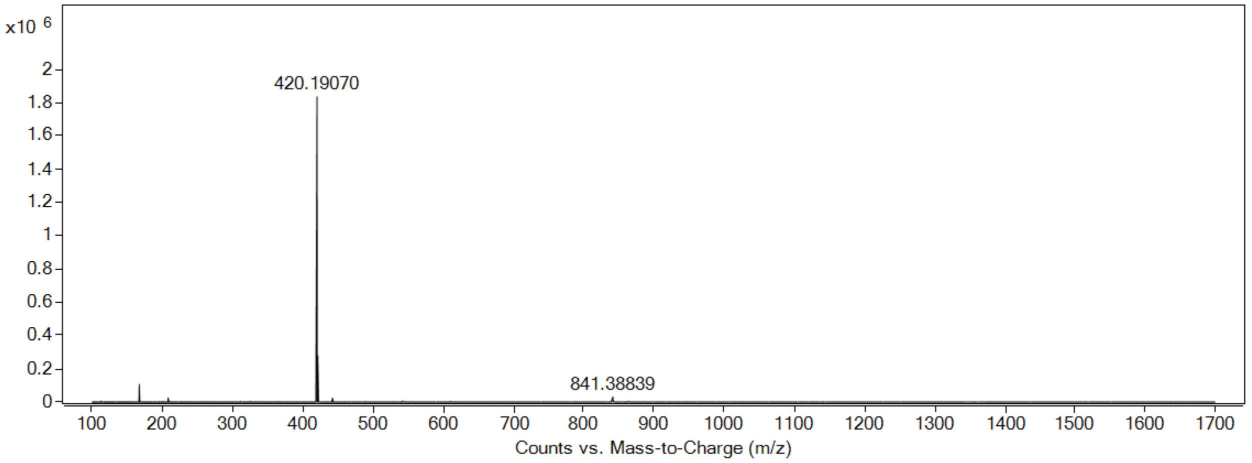

Figure S40. HR-MS (-) of compound **10**.

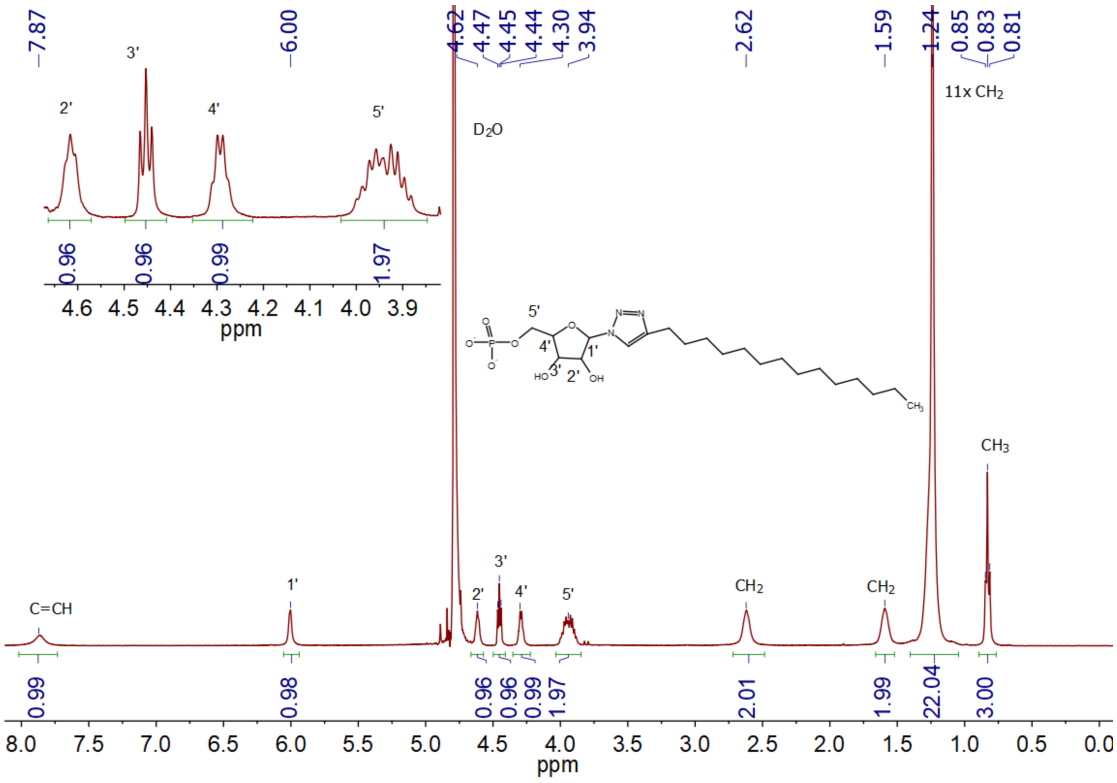

Figure S41. ^{1}H NMR spectrum of compound **11** (D_2O, 400 MHz, 295 K).

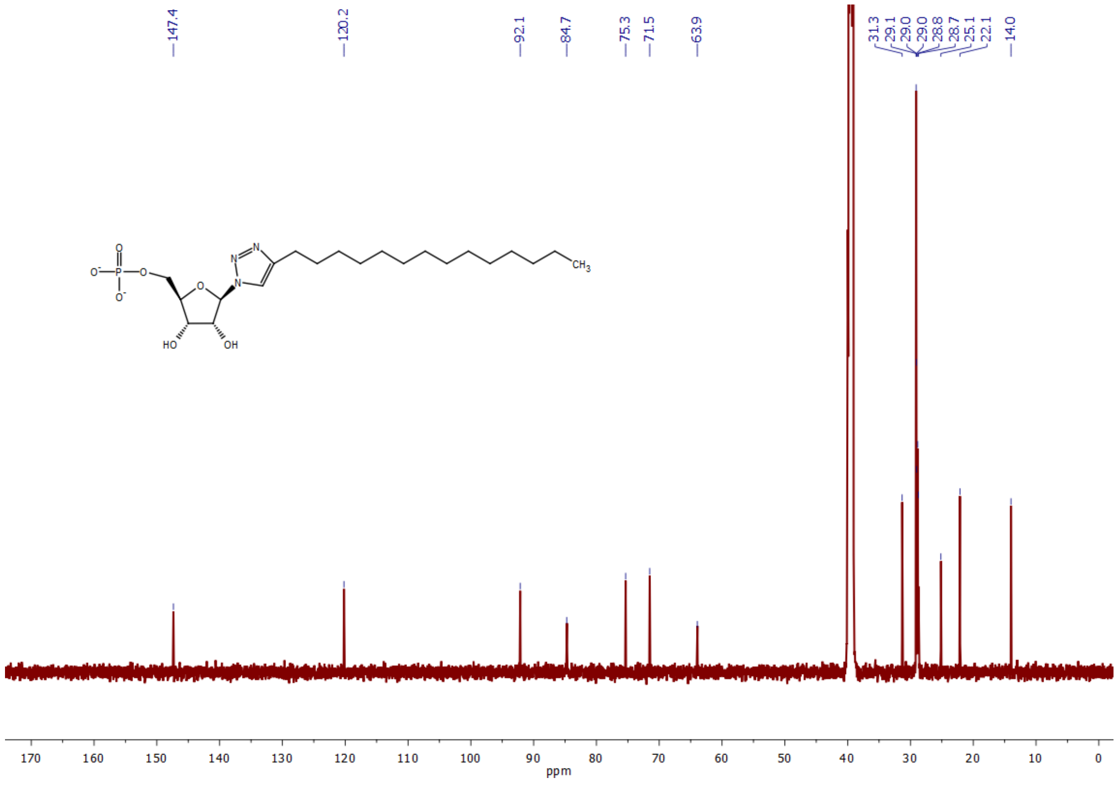

Figure S42. ^{13}C NMR spectrum of compound **11** (DMSO-d_6, 101 MHz, 295 K).

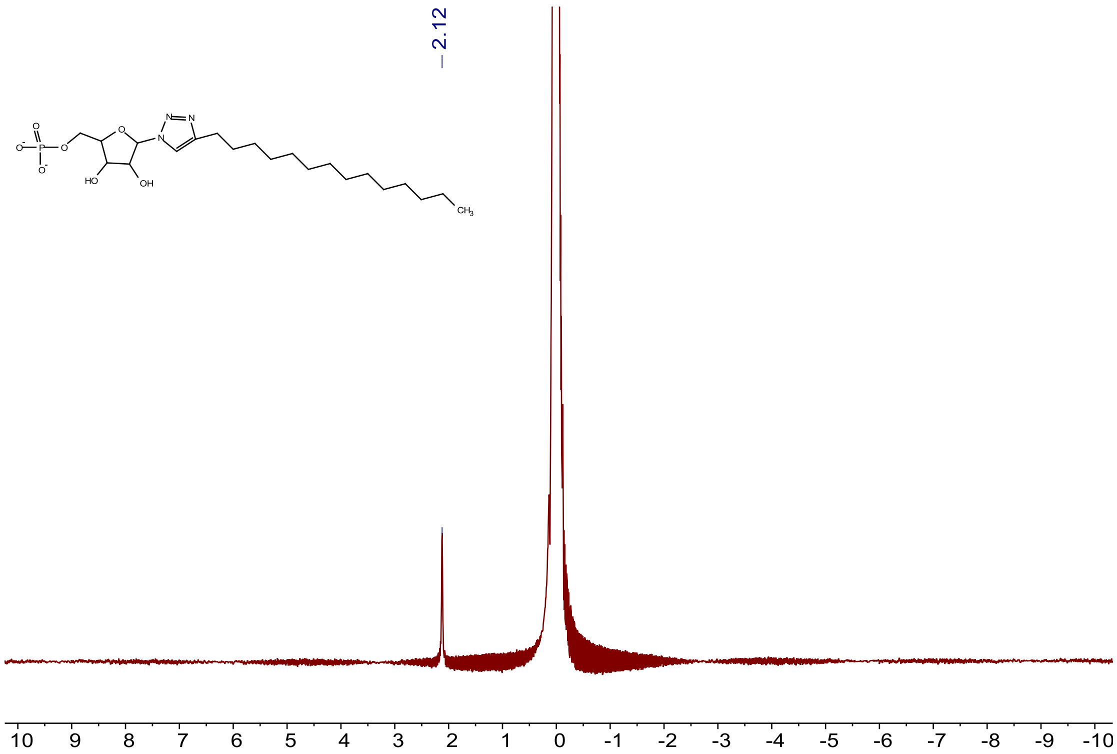

Figure S43. ^{31}P NMR spectrum of compound **11** (MeOH-d4, against 85% H_3PO_4, 162 MHz, 295 K)

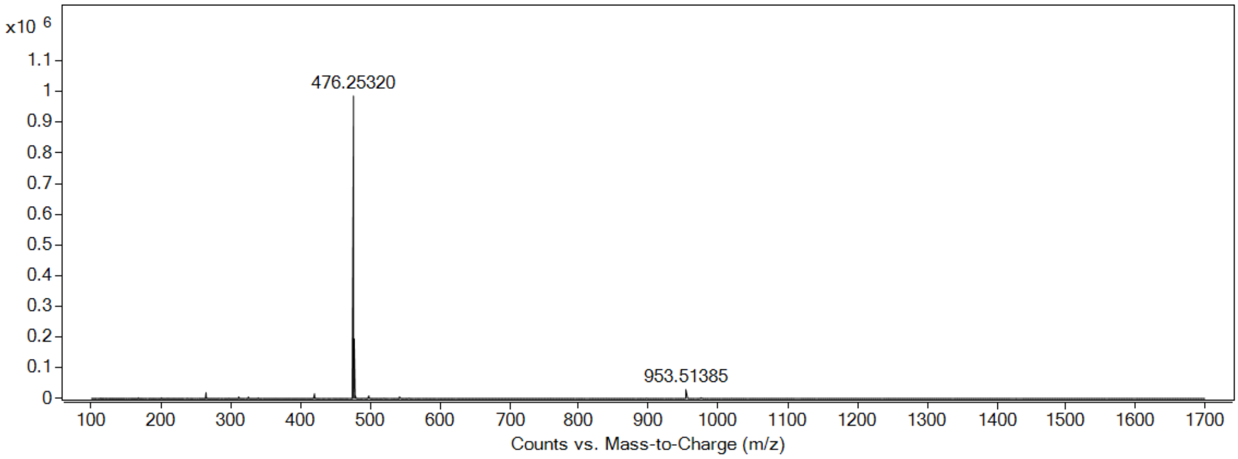

Figure S44. HR-MS (-) of compound **11**.

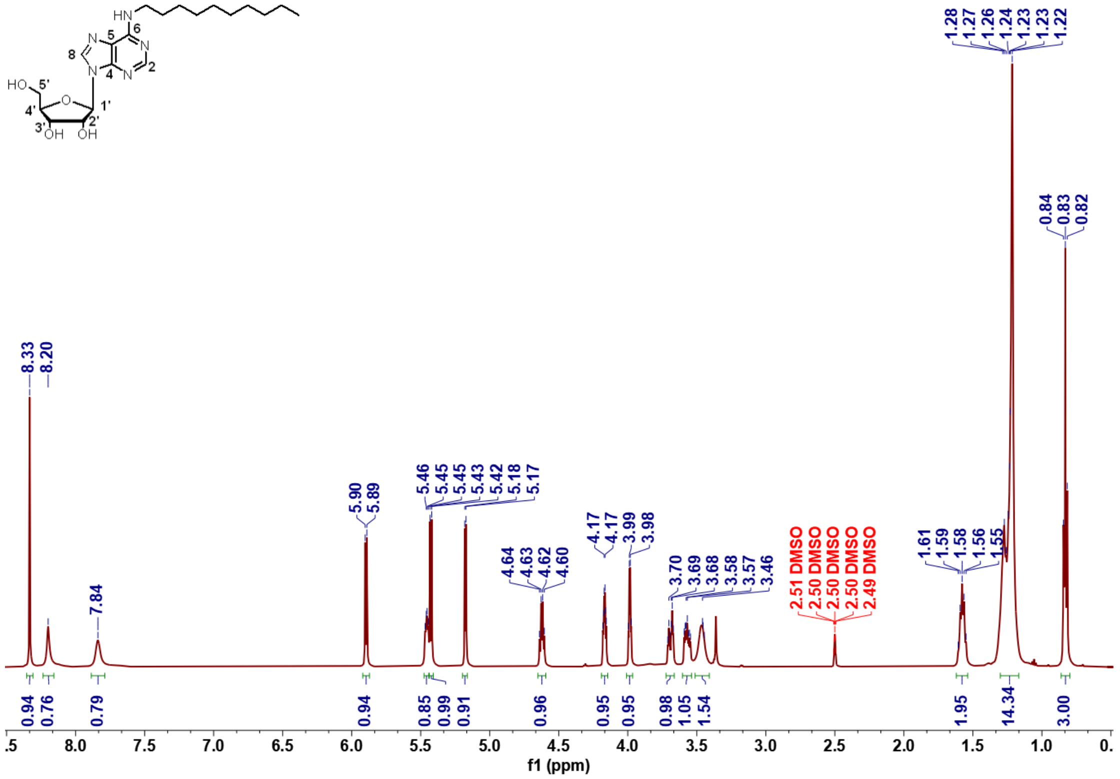

Figure S45. ^{1}H NMR spectrum of compound 13 (DMSO-d6, 400 MHz, 295 K).

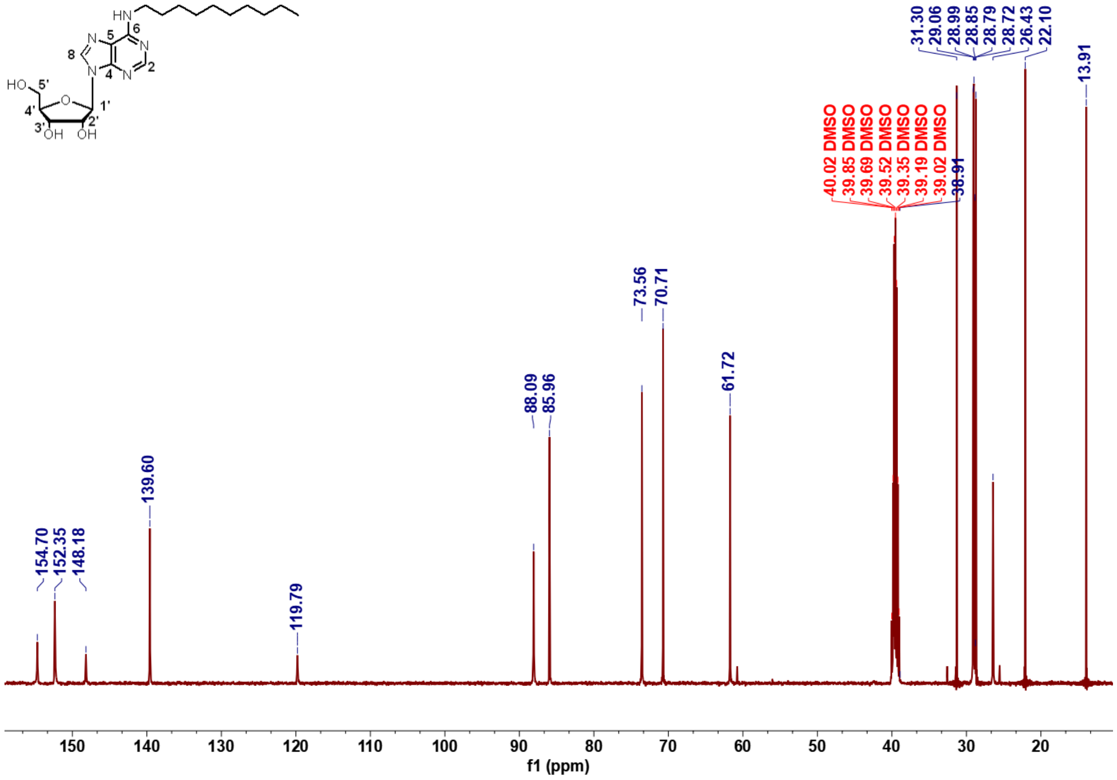

Figure S46. ^{13}C NMR spectrum of compound 13 (DMSO-d6, 100 MHz, 295 K).

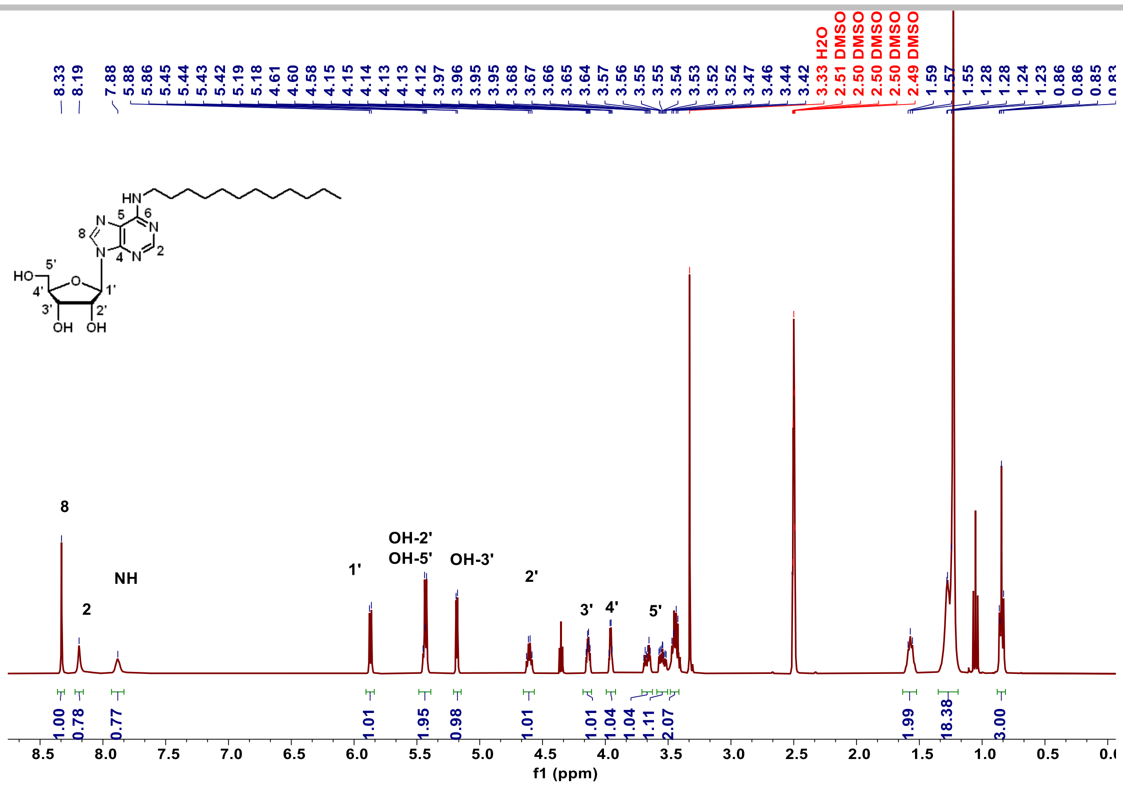

Figure S47. ^{1}H NMR spectrum of compound **14** (DMSO-d6, 400 MHz, 295 K).

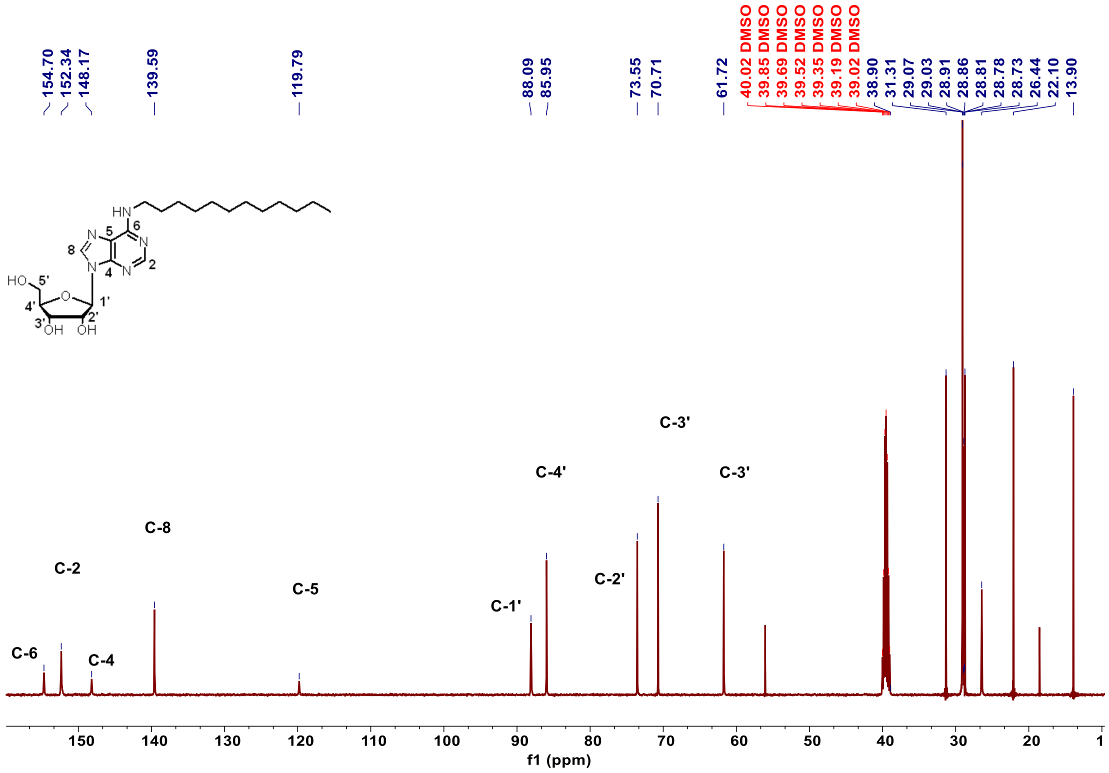

Figure S48. ^{13}C NMR spectrum of compound **14** (DMSO-d6, 100 MHz, 295 K).

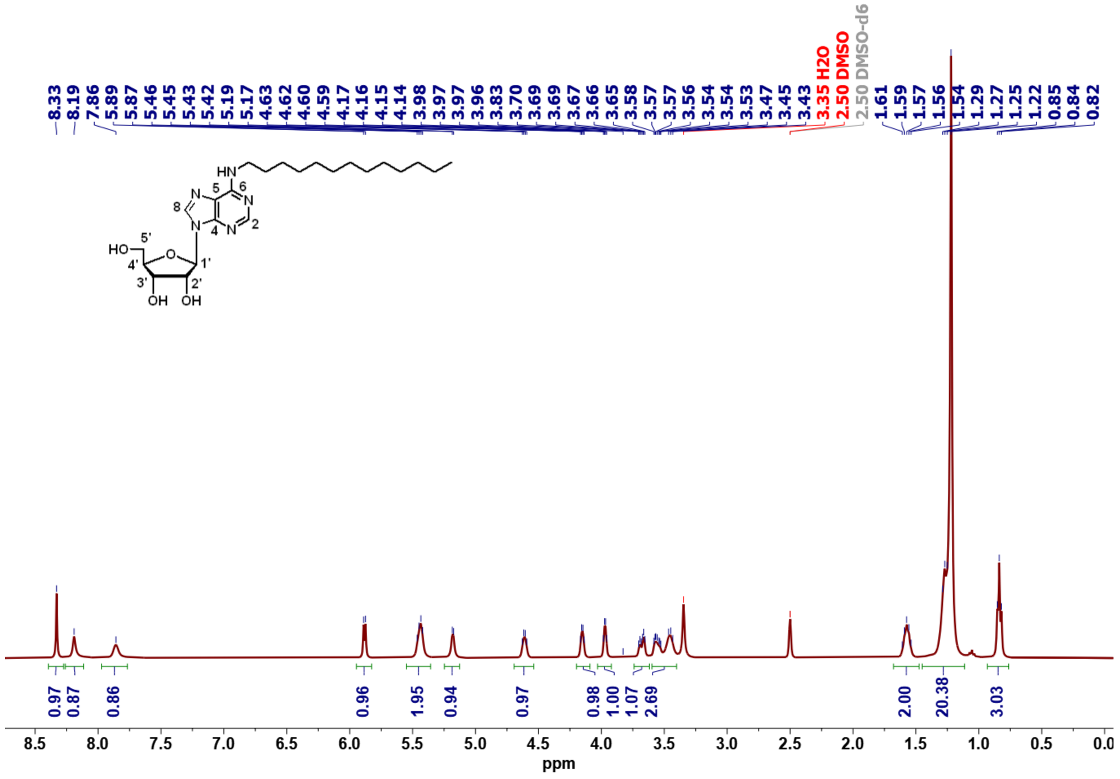

Figure S49. ^{1}H NMR spectrum of compound **15** (DMSO-d$_6$, 400 MHz, 295 K).

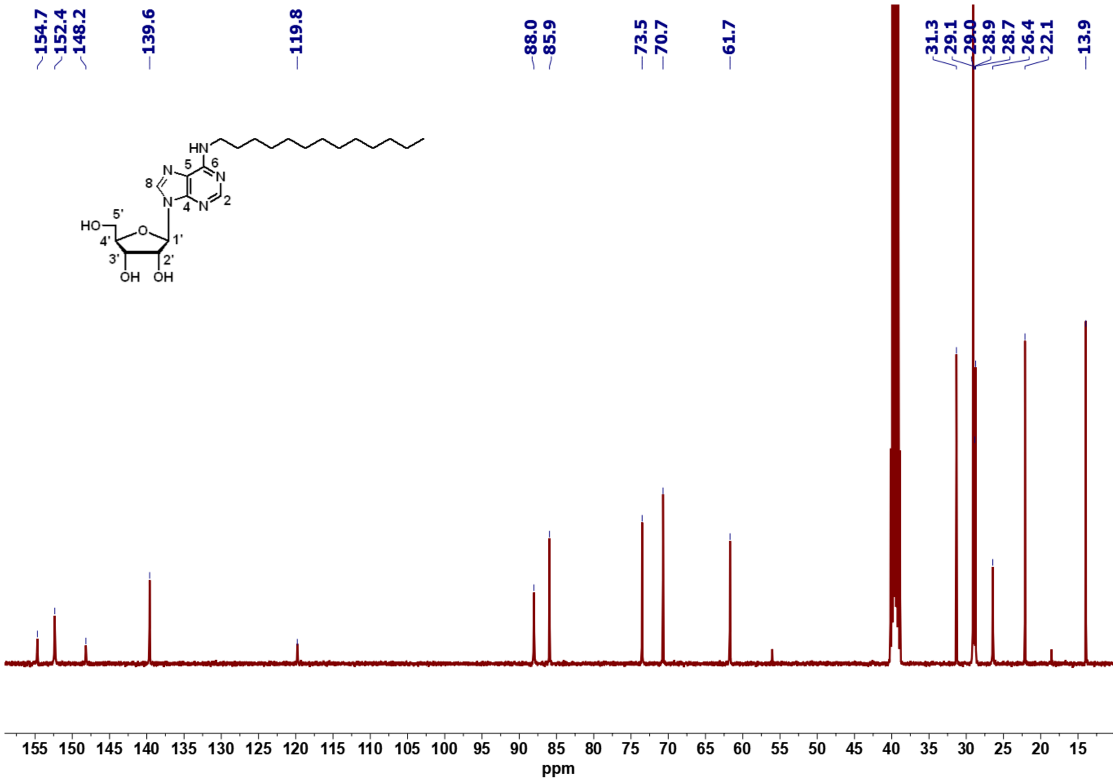

Figure S50. ^{13}C NMR spectrum of compound **15** (DMSO-d$_6$, 100 MHz, 295 K).

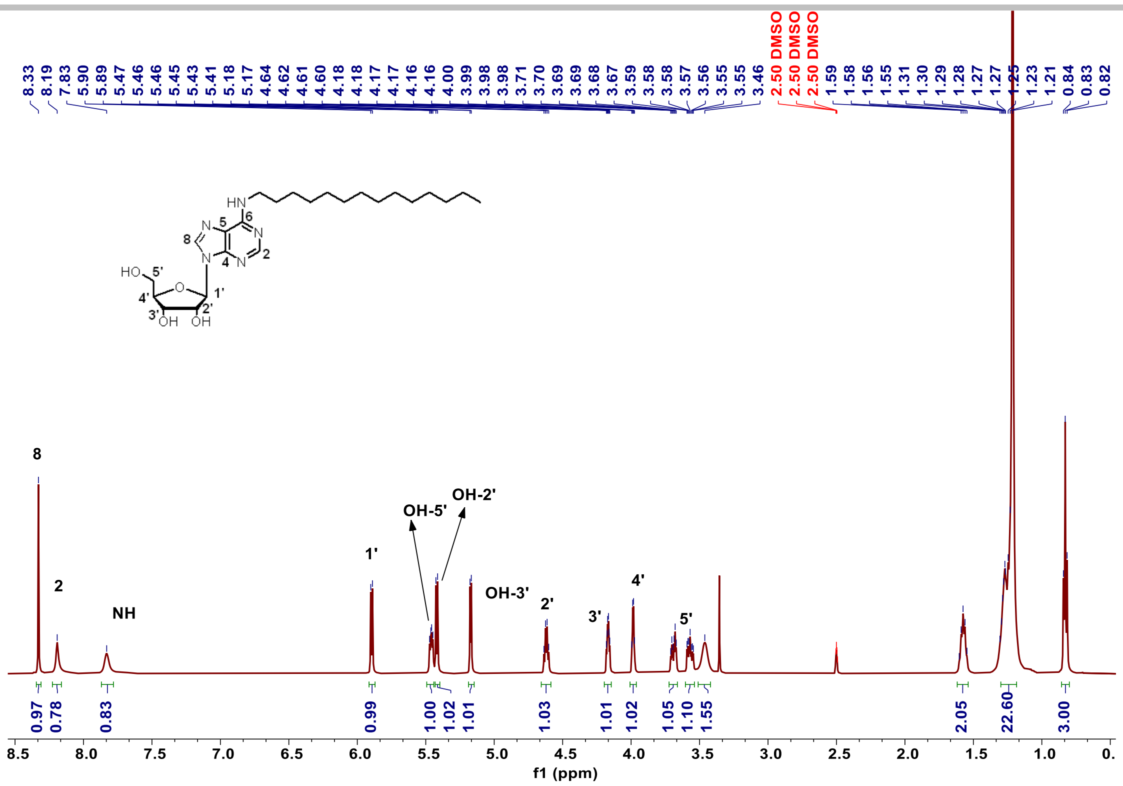

Figure S51. ^{1}H NMR spectrum of compound **16** (DMSO-d6, 400 MHz, 295 K).

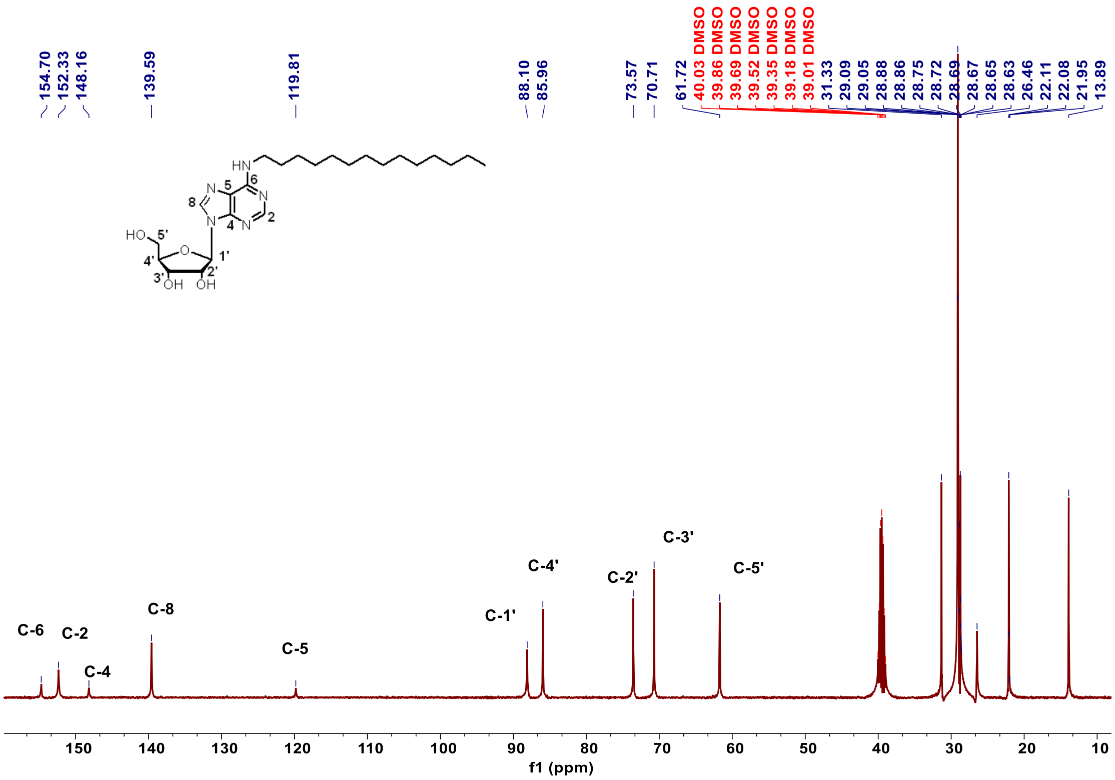

Figure S52. ^{13}C NMR spectrum of compound **16** (DMSO-d6, 100 MHz, 295 K).

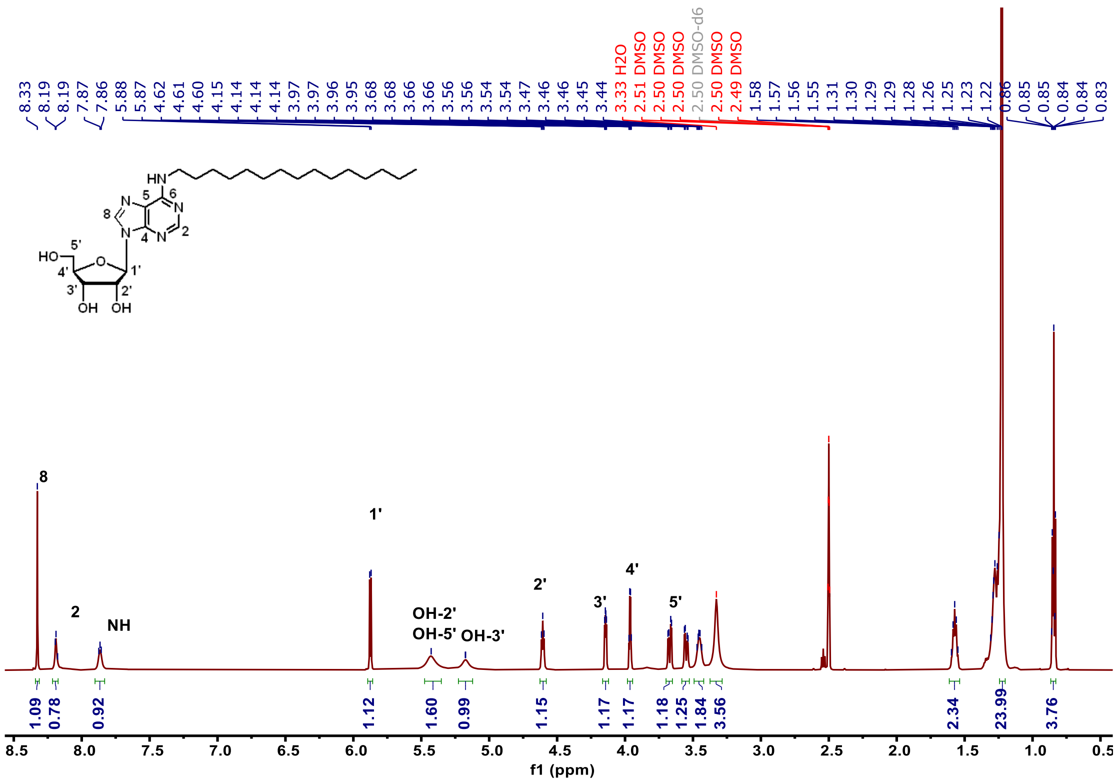

Figure S53. ^{1}H NMR spectrum of compound **17** (DMSO-d6, 400 MHz, 295 K).

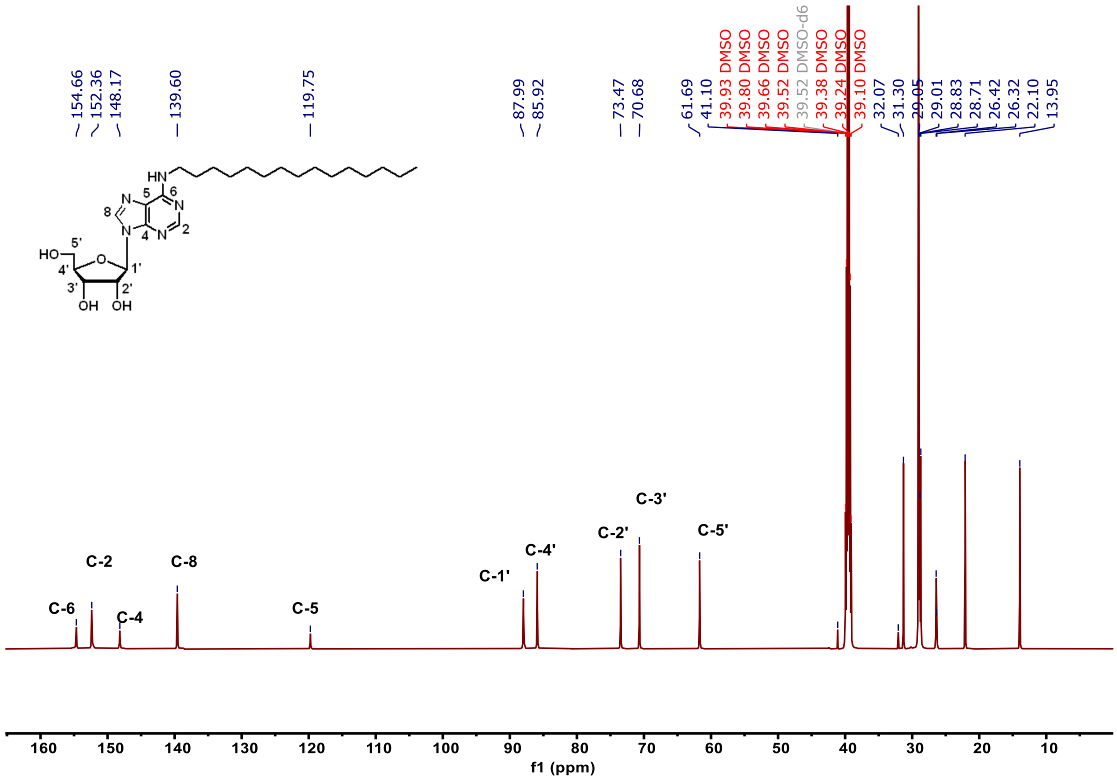

Figure S54. ^{13}C NMR spectrum of compound **17** (DMSO-d6, 100 MHz, 295 K).

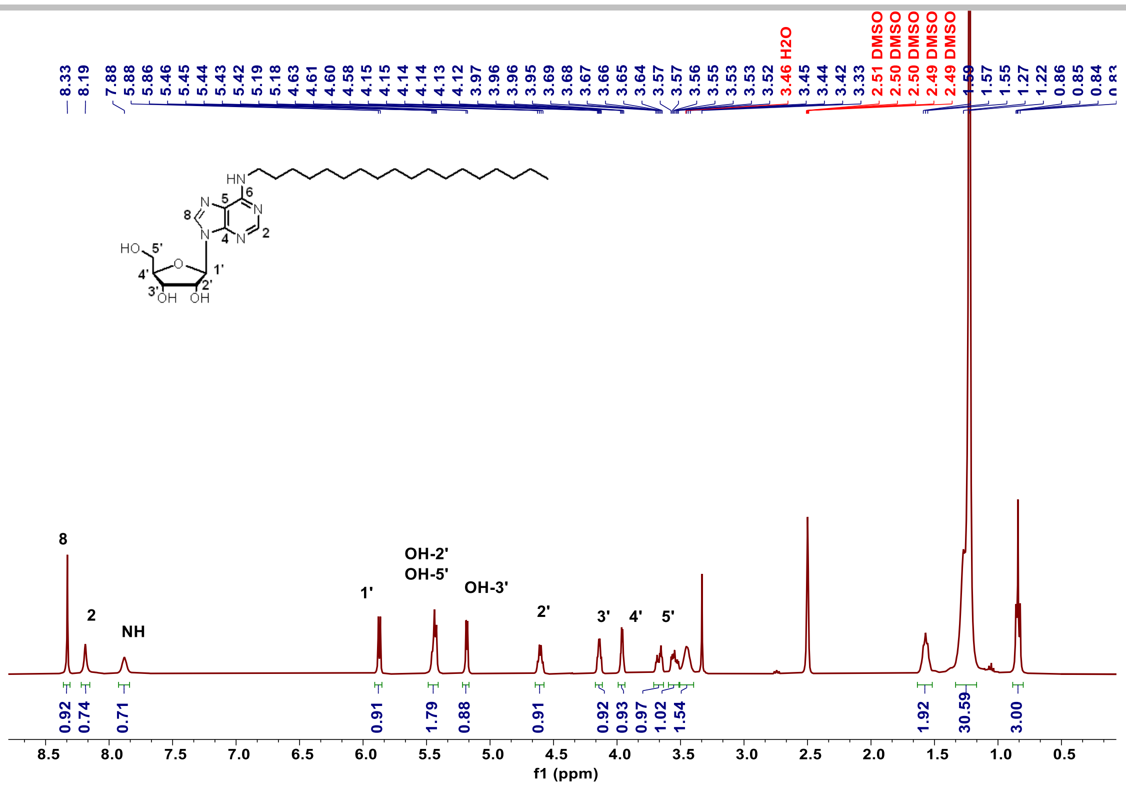

Figure S55. ^{1}H NMR spectrum of compound **18** ($DMSO-d_6$, 400 MHz, 295 K).

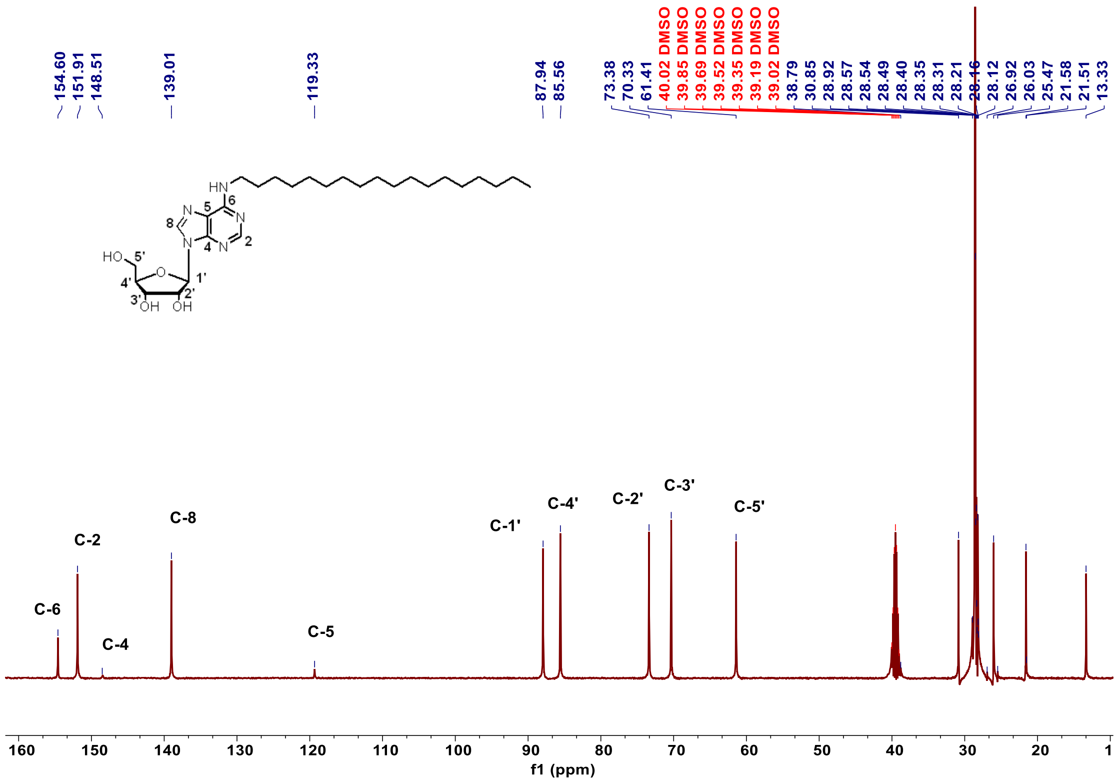

Figure S56. ^{13}C NMR spectrum of compound **18** ($DMSO-d_6$, 100 MHz, 295 K).

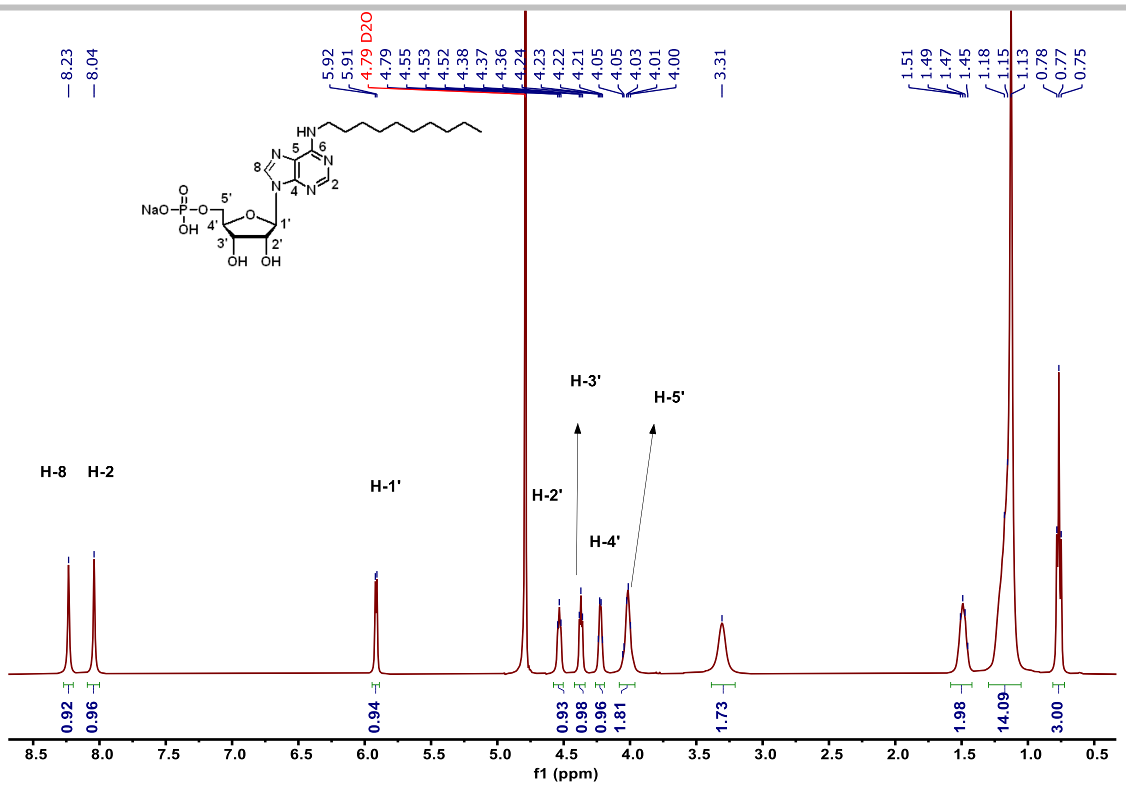

Figure S57. ^{1}H NMR spectrum of compound **19** (D_2O, 400 MHz, 295 K).

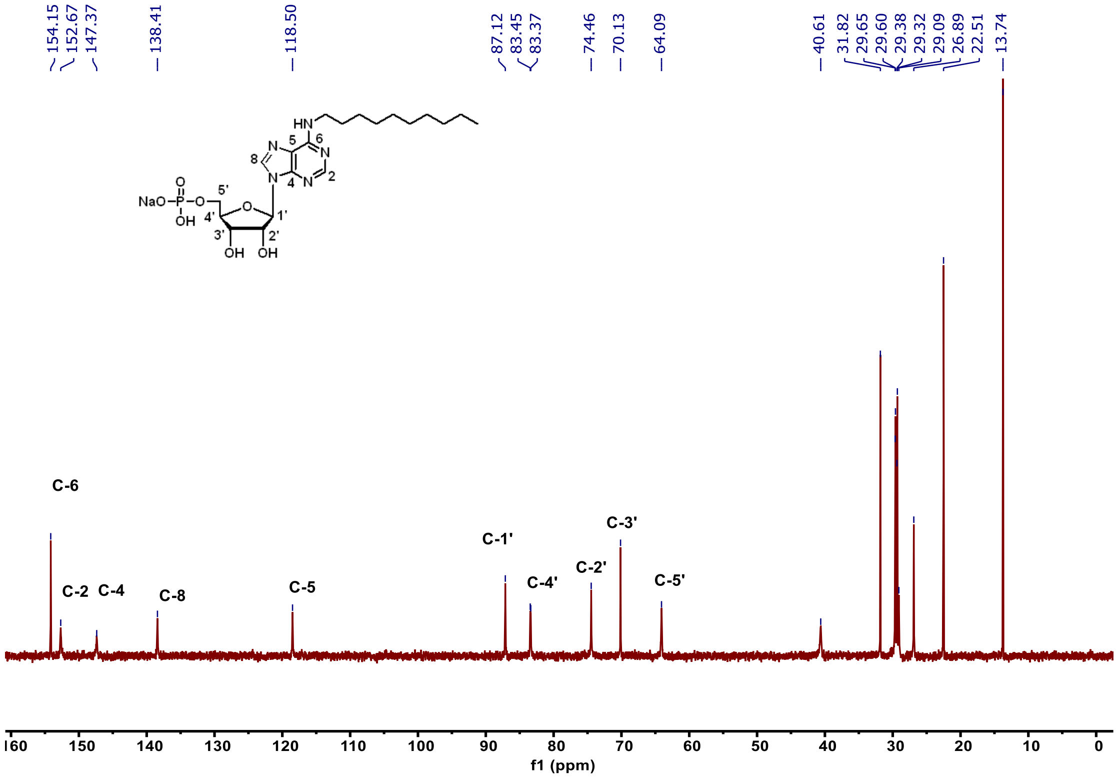

Figure S58. ^{13}C NMR spectrum of compound **19** (D_2O, 100 MHz, 295 K).

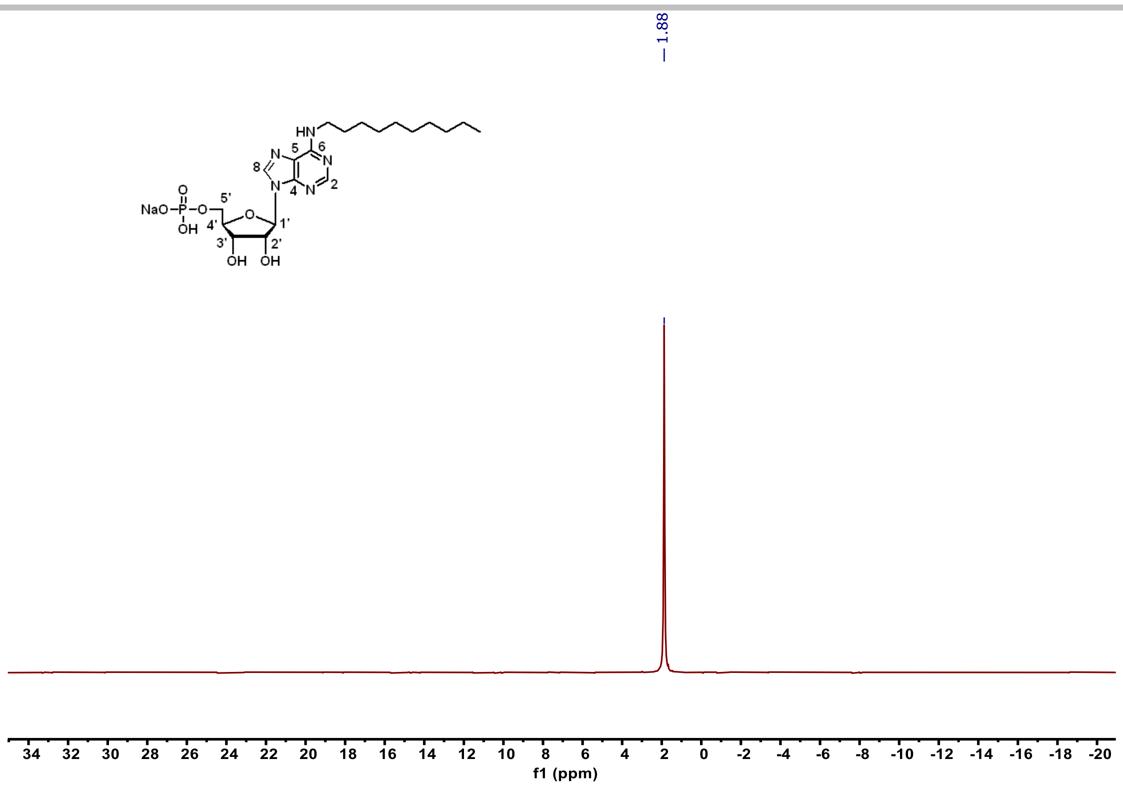

Figure S59. ^{31}P NMR spectrum of compound **19** (D_2O, 162 MHz, 295 K).

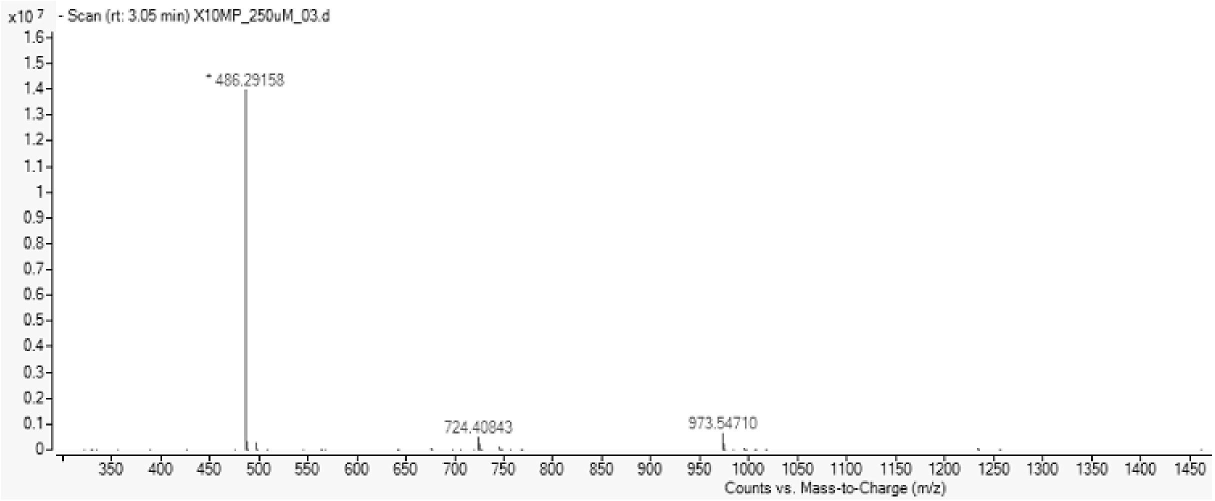

Figure S60. HR-MS (-) of compound **19**.

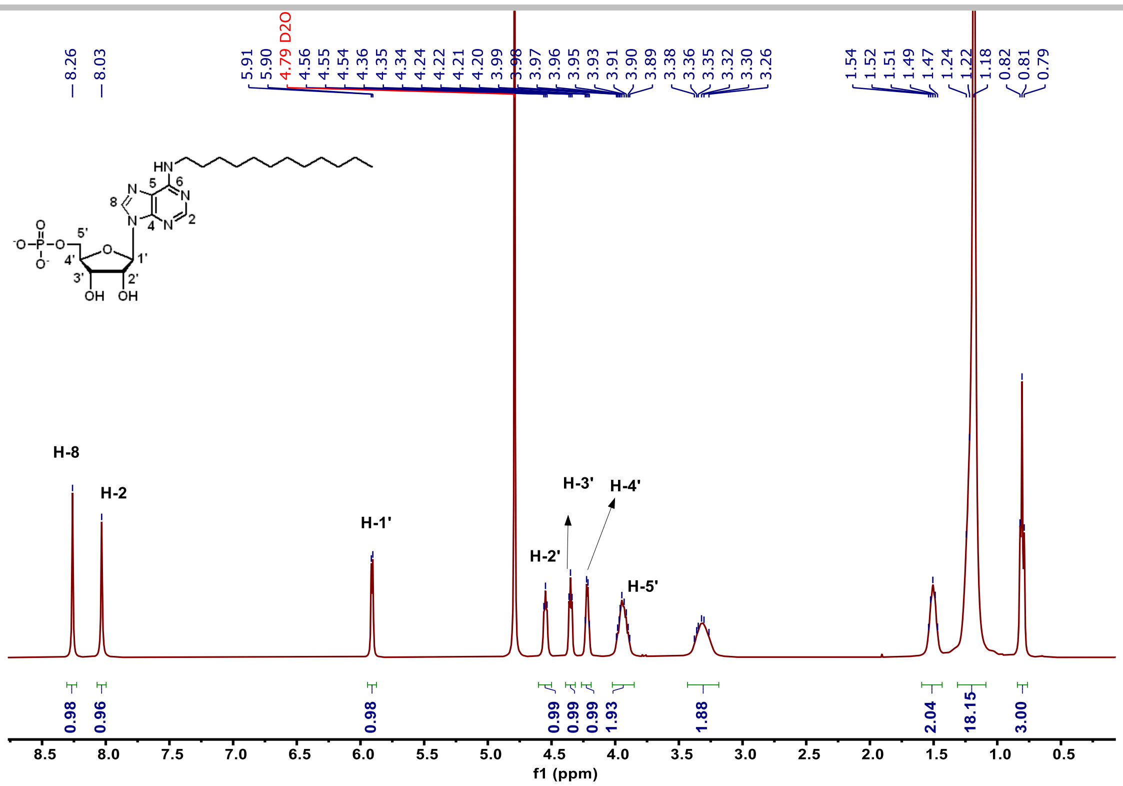

Figure S61. ^{1}H NMR spectrum of compound **20** (D_2O, 400 MHz, 295 K).

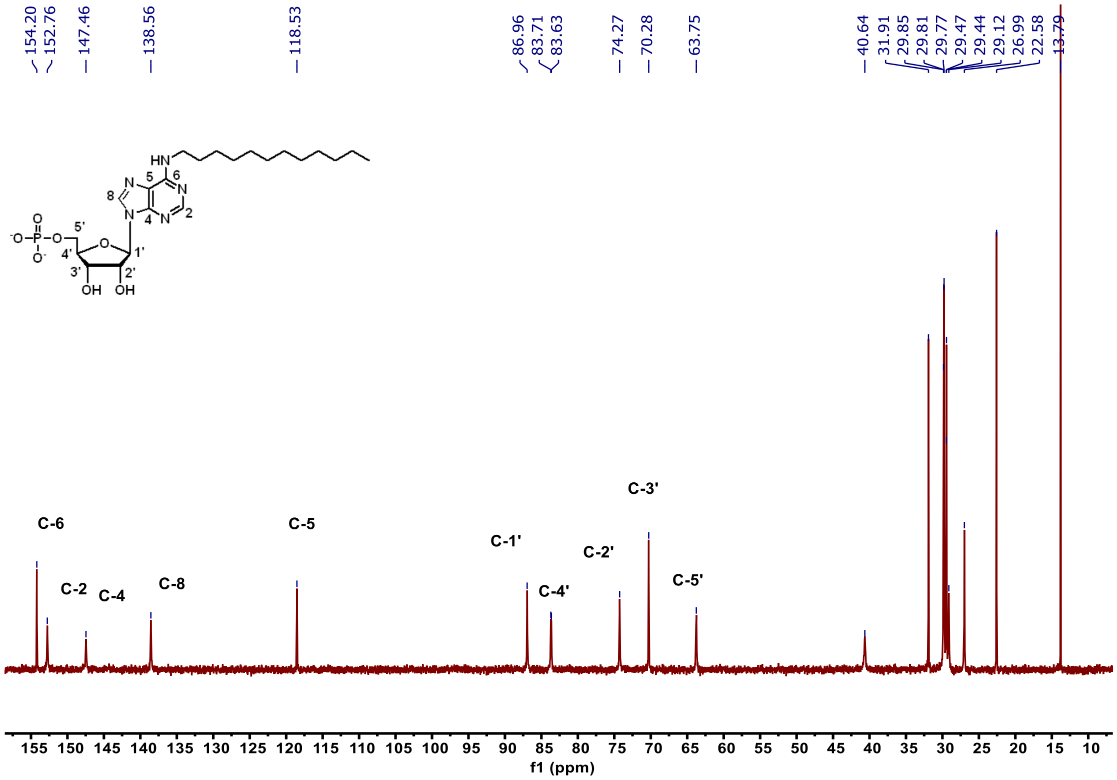

Figure S62. ^{13}C NMR spectrum of compound **20** (D_2O, 100 MHz, 295 K).

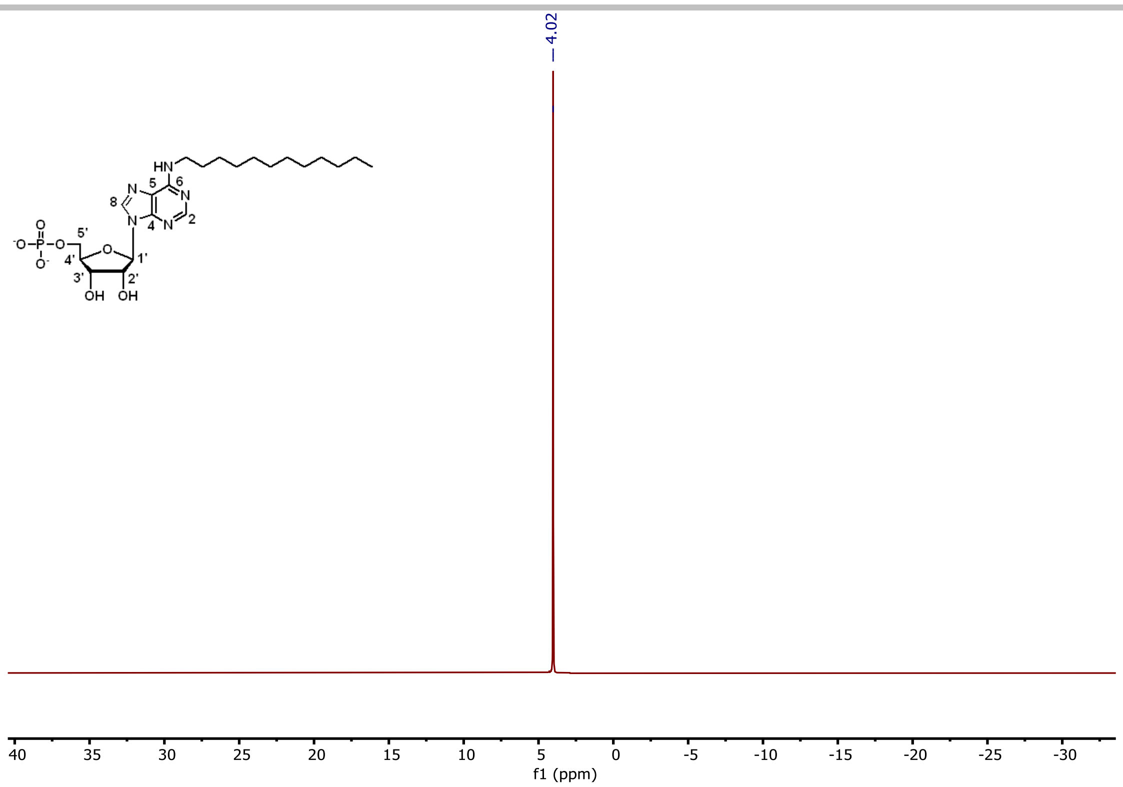

Figure S63. ^{31}P NMR spectrum of compound **20** (D_2O, 162 MHz, 295 K).

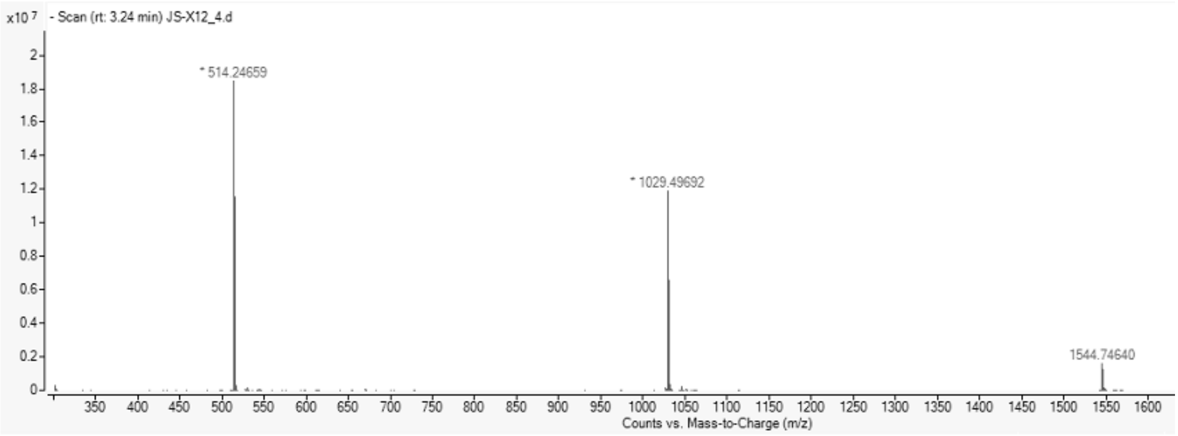

Figure S64. HR-MS (-) of compound **20**.

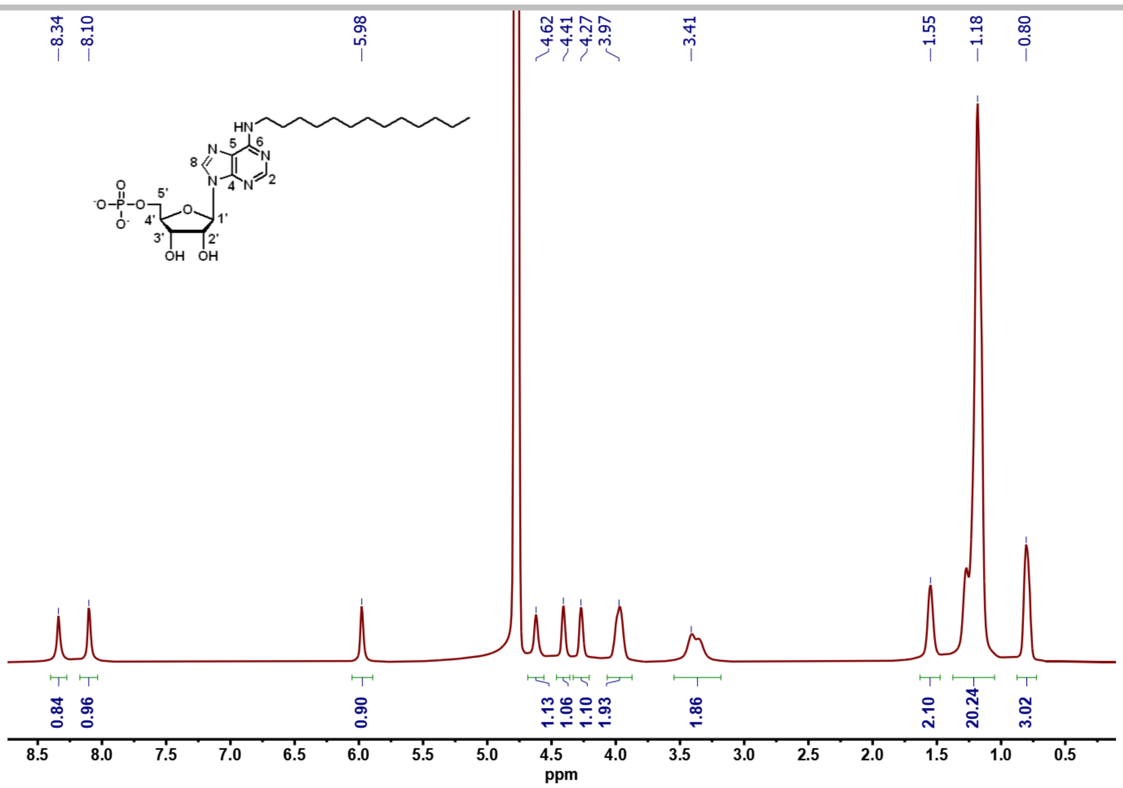

Figure S65. ^{1}H NMR spectrum of compound **21** (D_2O, 600 MHz, 295 K).

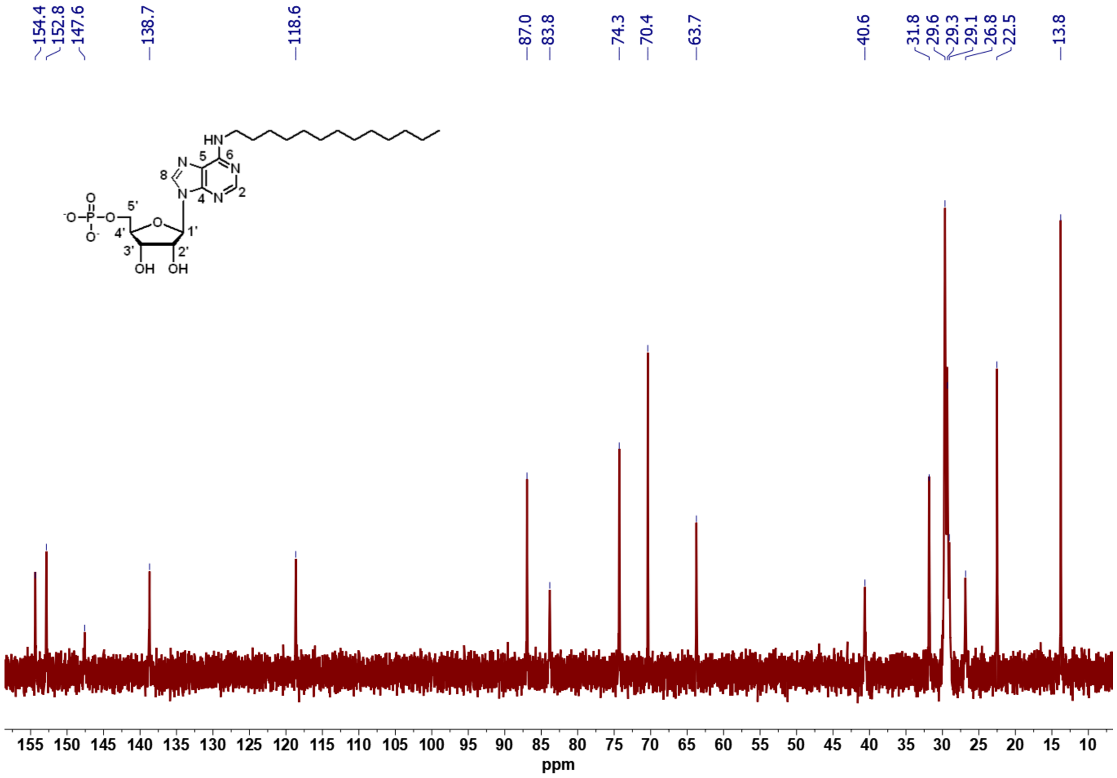

Figure S66. ^{13}C NMR spectrum of compound **21** (D_2O, 150 MHz, 295 K).

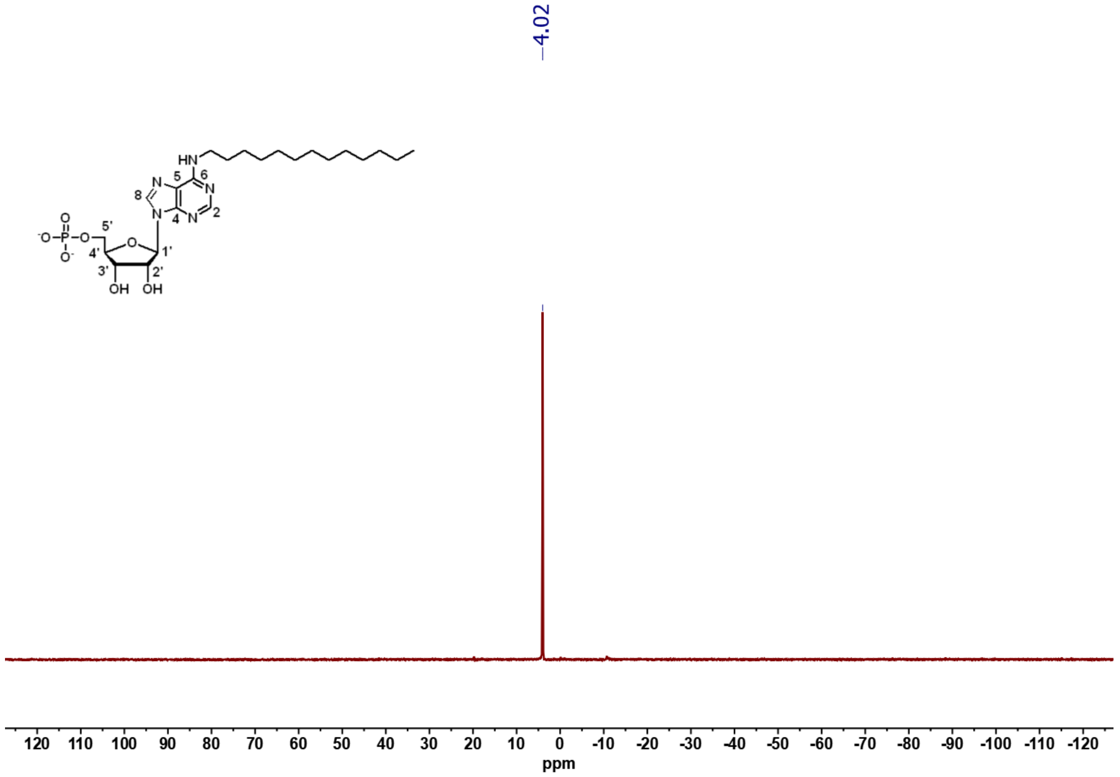

Figure S67. ^{31}P NMR spectrum of compound **21** (D_2O, 243 MHz, 295 K).

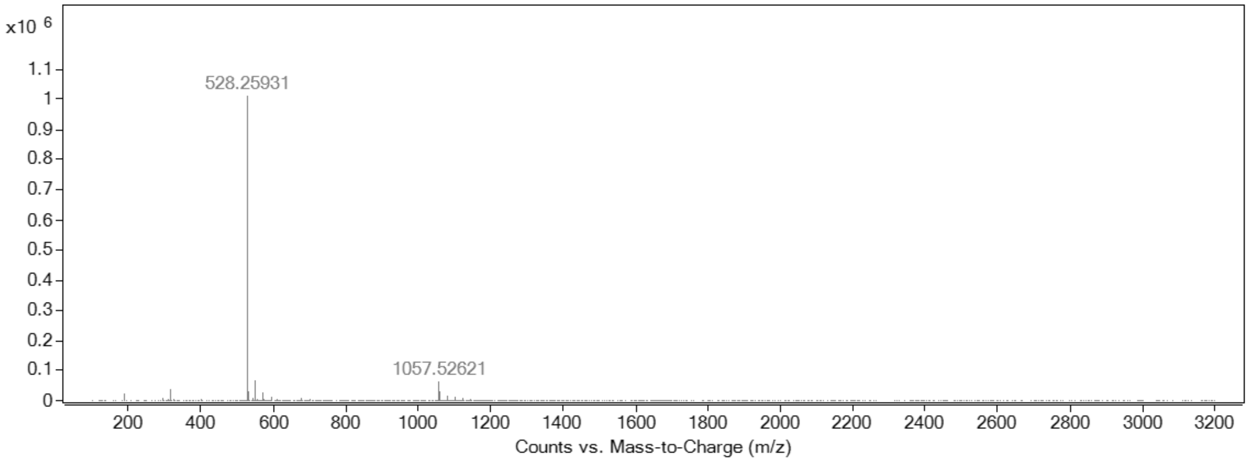

Figure S68. HR-MS (-) of compound **21**.

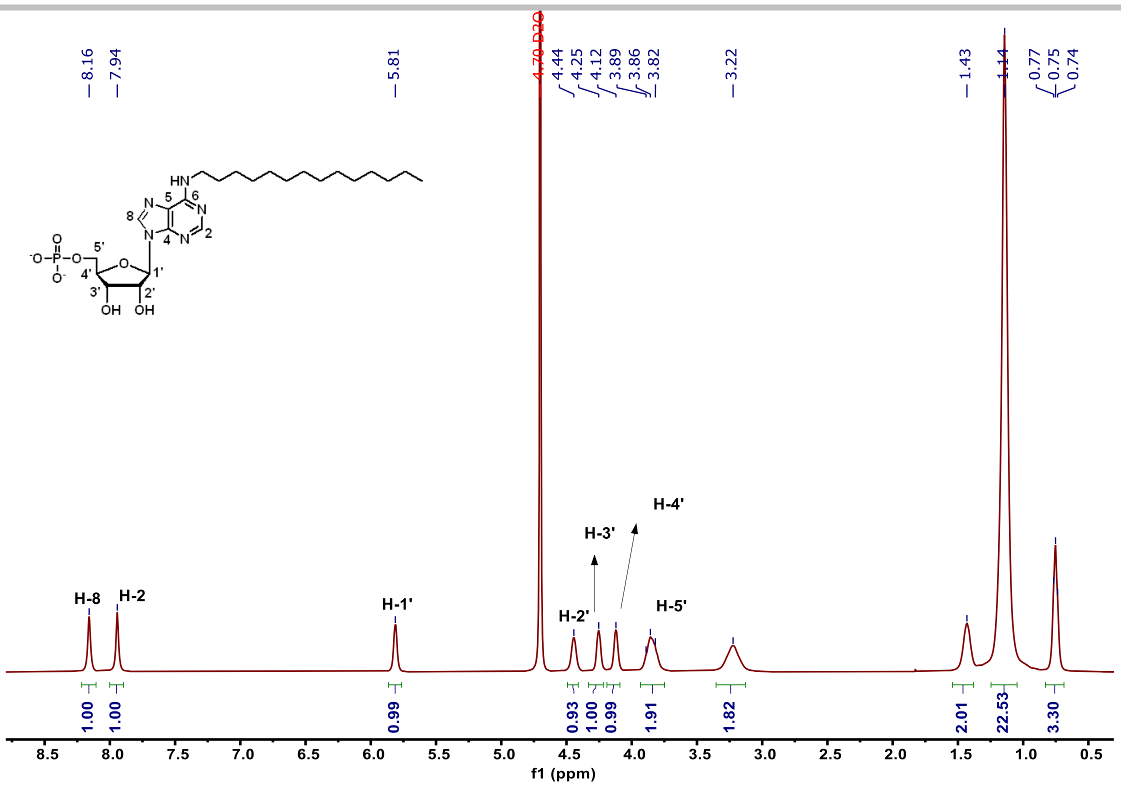

Figure S69. ^{1}H NMR spectrum of compound **22** (D_2O, 400 MHz, 295 K).

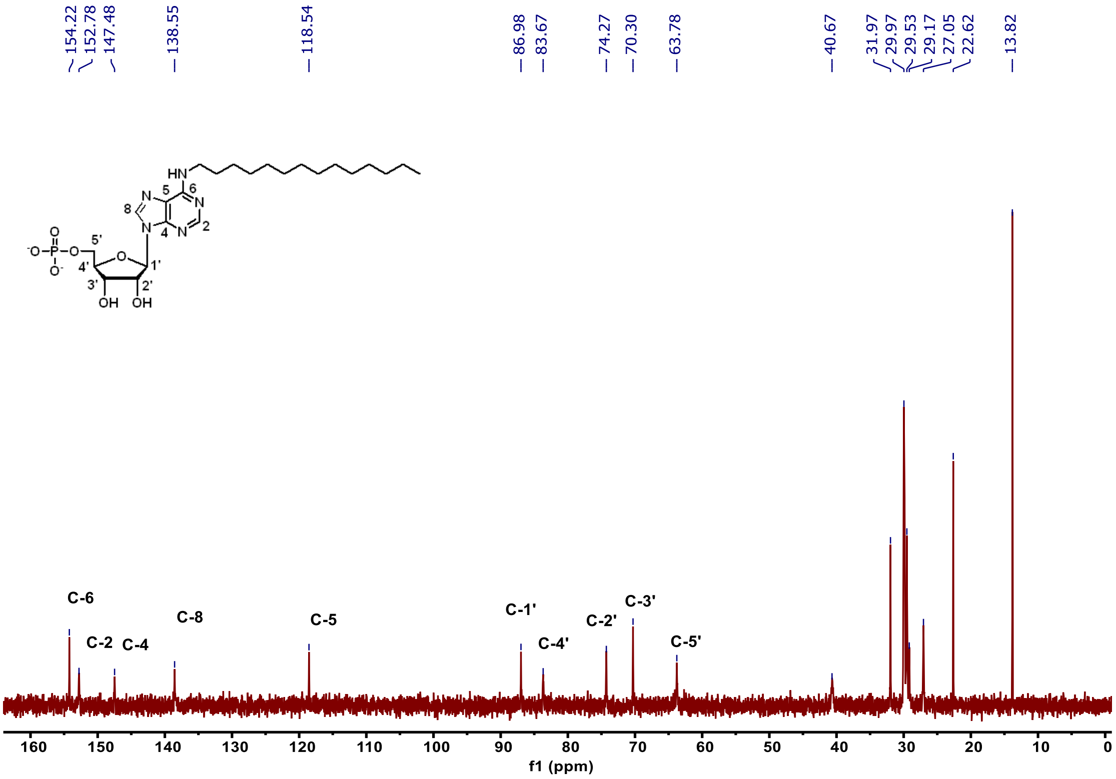

Figure S70. ^{13}C NMR spectrum of compound **22** (D_2O, 100 MHz, 295 K).

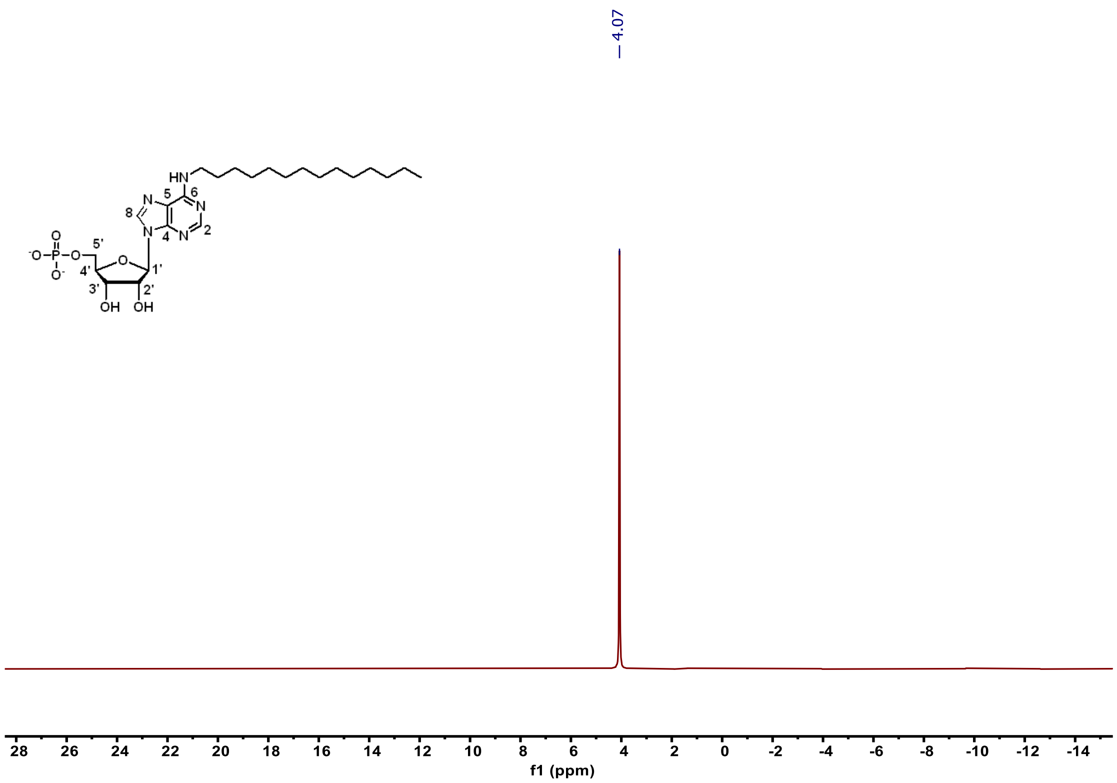

Figure S71. ^{31}P NMR spectrum of compound **22** (D_2O, 162 MHz, 295 K).

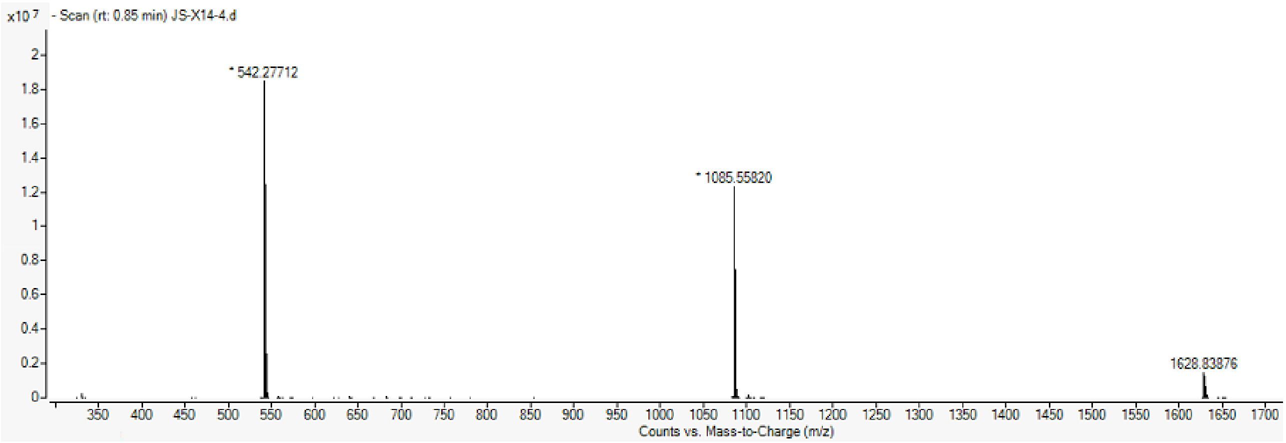

Figure S72. HR-MS (-) of compound **22**.

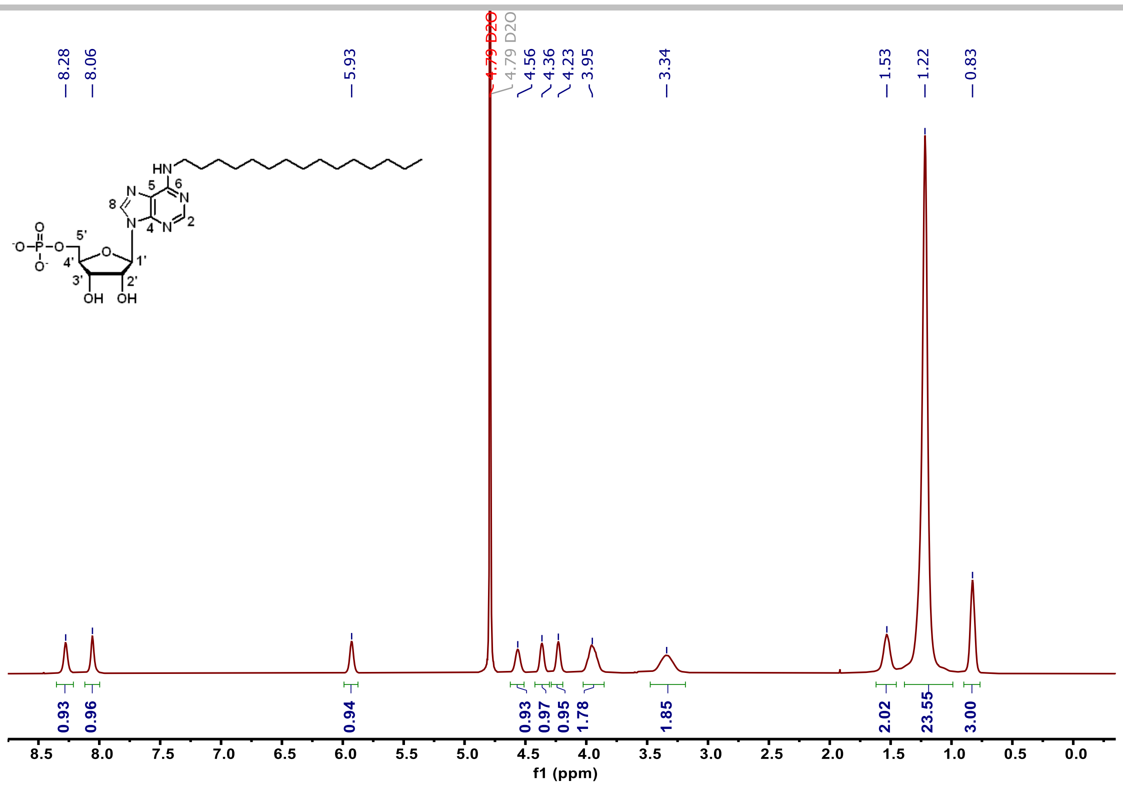

Figure S73. ^{1}H NMR spectrum of compound **23** (D_2O, 400 MHz, 295 K).

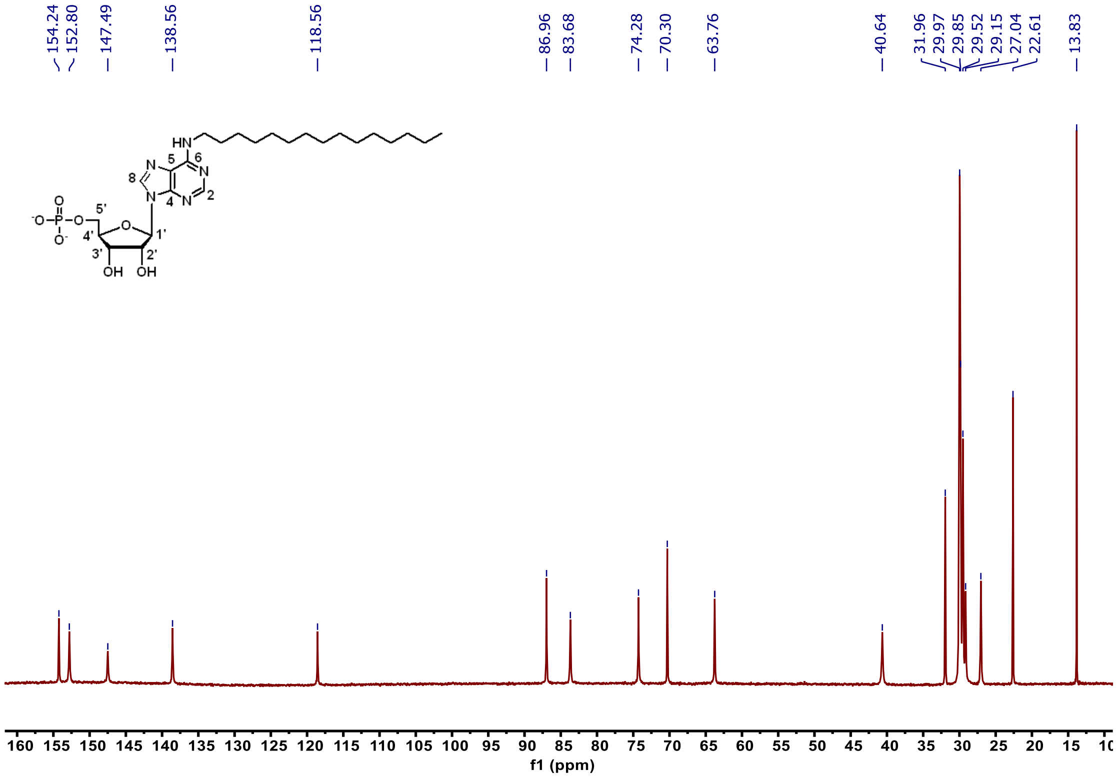

Figure S74. ^{13}C NMR spectrum of compound **23** (D_2O, 100 MHz, 295 K).

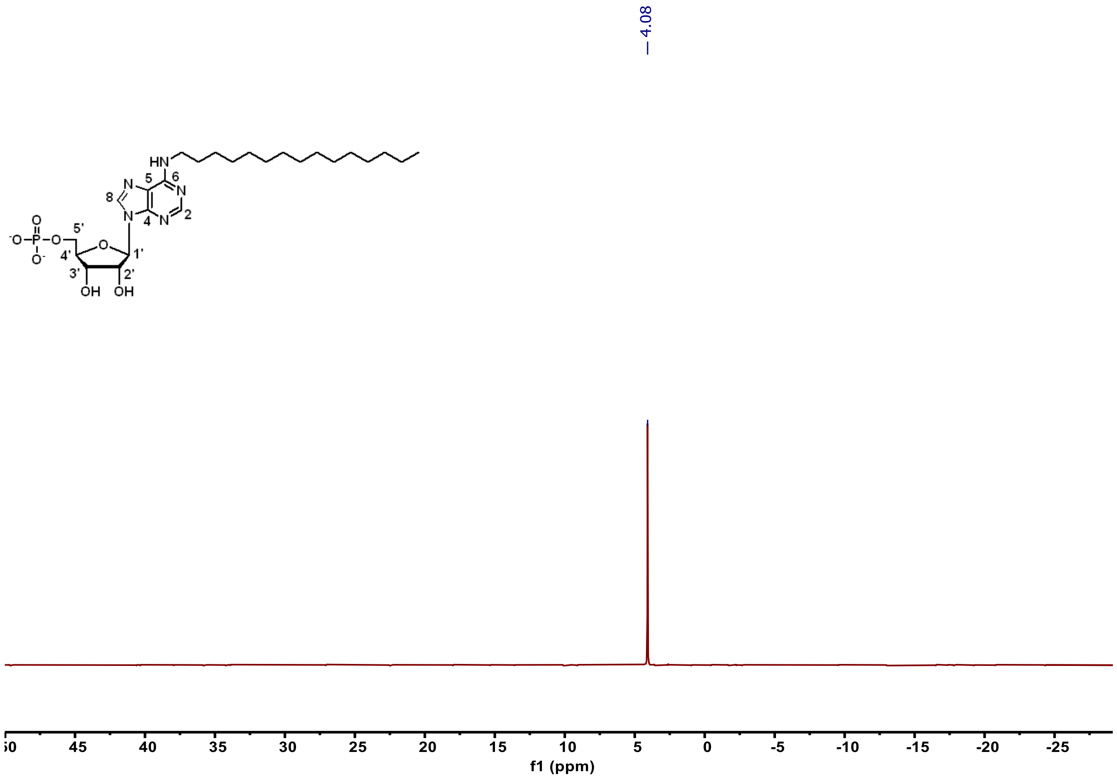

Figure S75. ^{31}P NMR spectrum of compound **23** (D_2O, 162 MHz, 295 K).

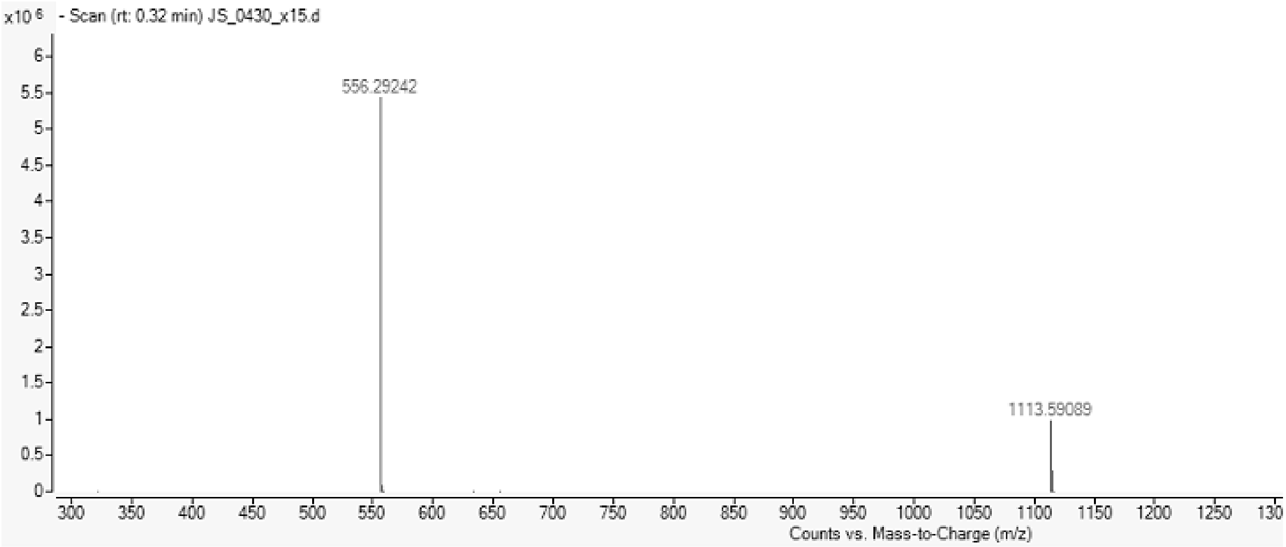

Figure S76. HR-MS (-) of compound **23**.

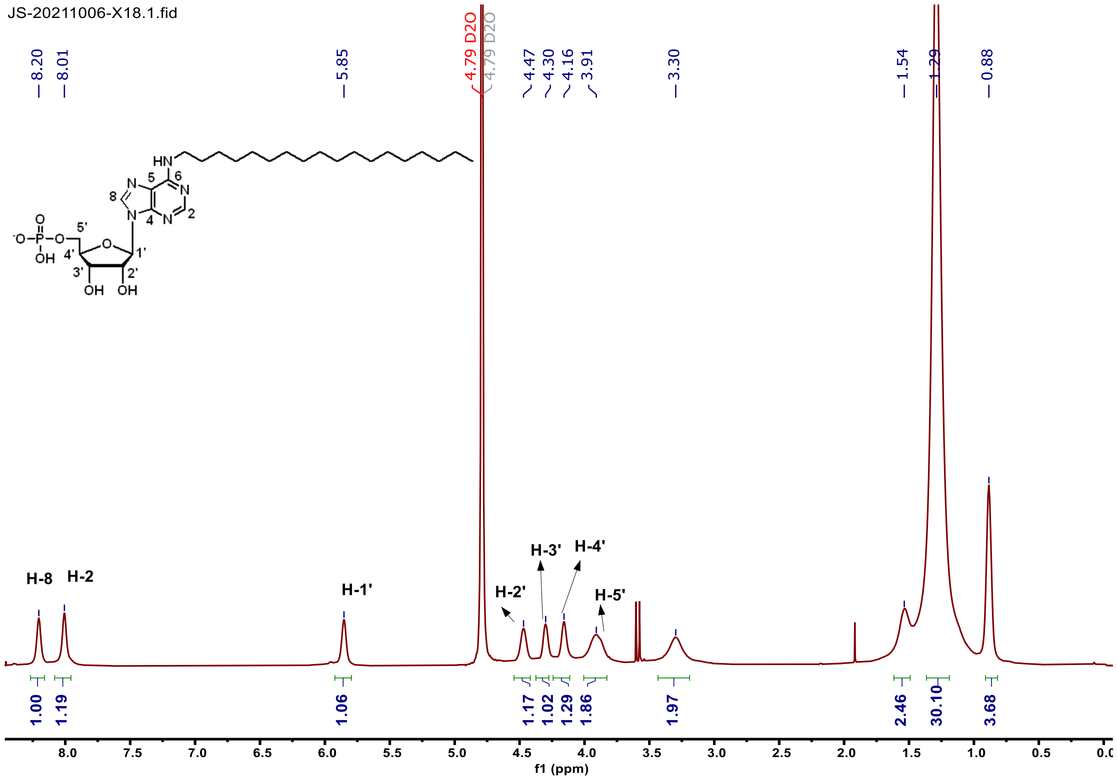

Figure S77. ^{1}H NMR spectrum of compound **24** (D_2O, 400 MHz, 295 K).

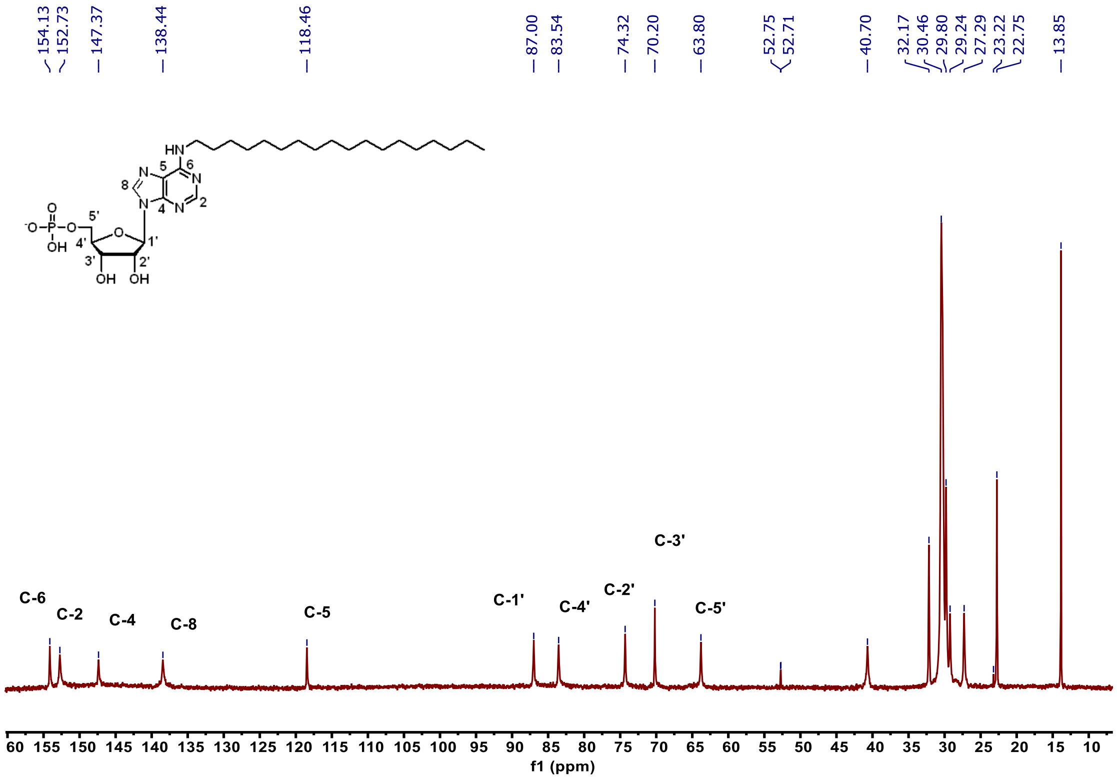

Figure S78. ^{13}C NMR spectrum of compound **24** (D_2O, 100 MHz, 295 K).

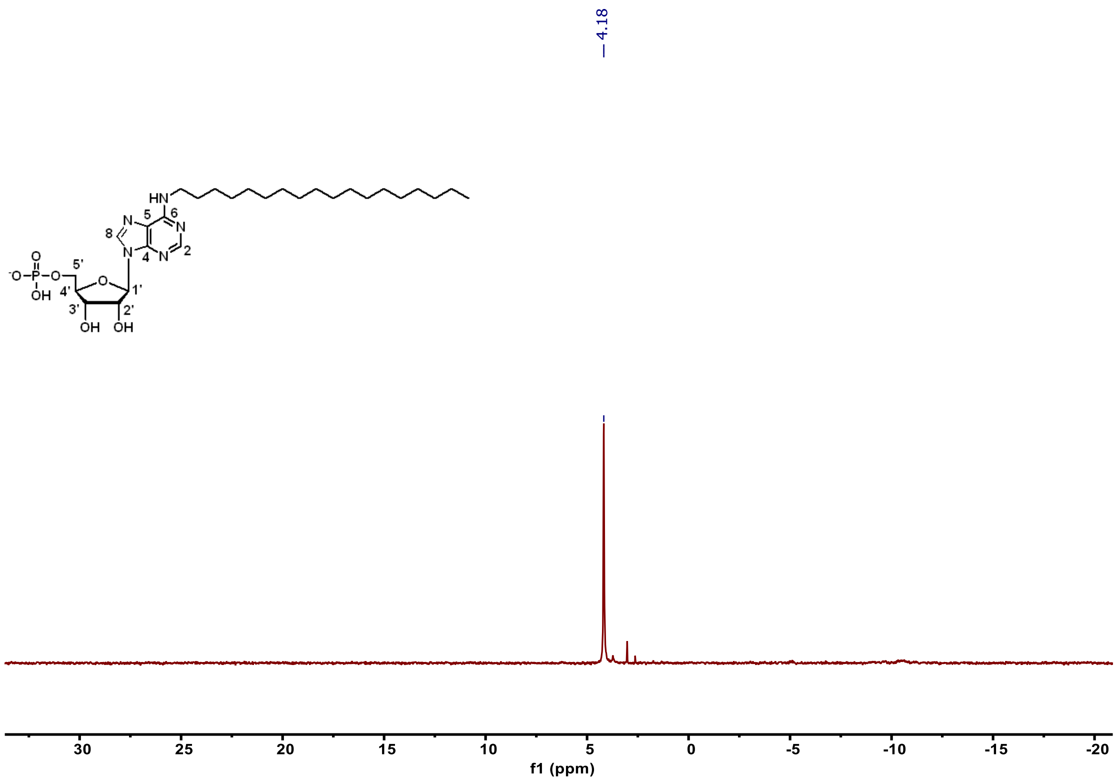

Figure S79. ^{31}P NMR spectrum of compound **24** (D_2O, 162 MHz, 295 K).

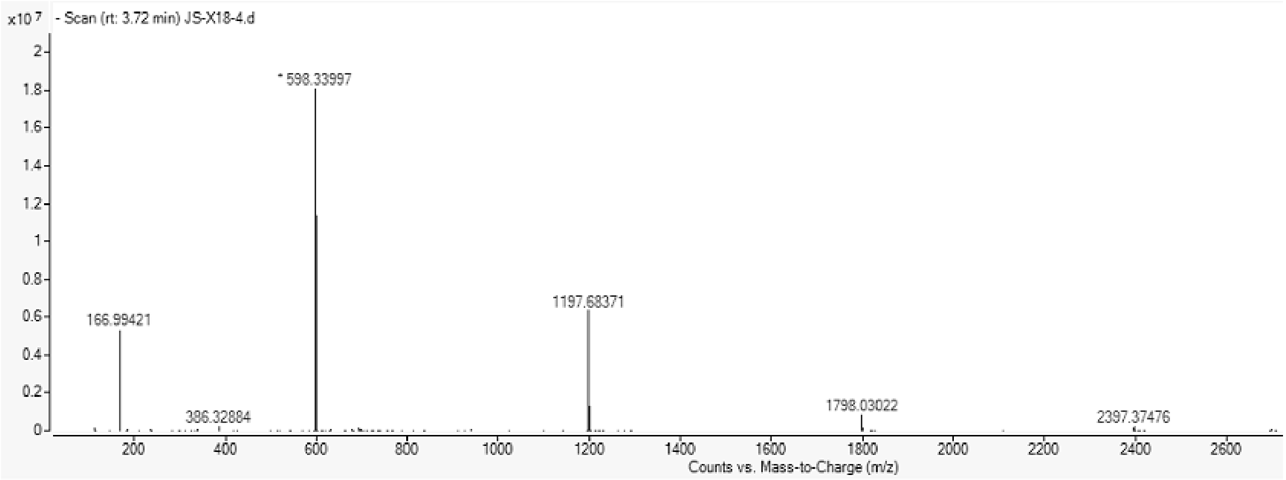

Figure S80. HR-MS (-) of compound **24**.

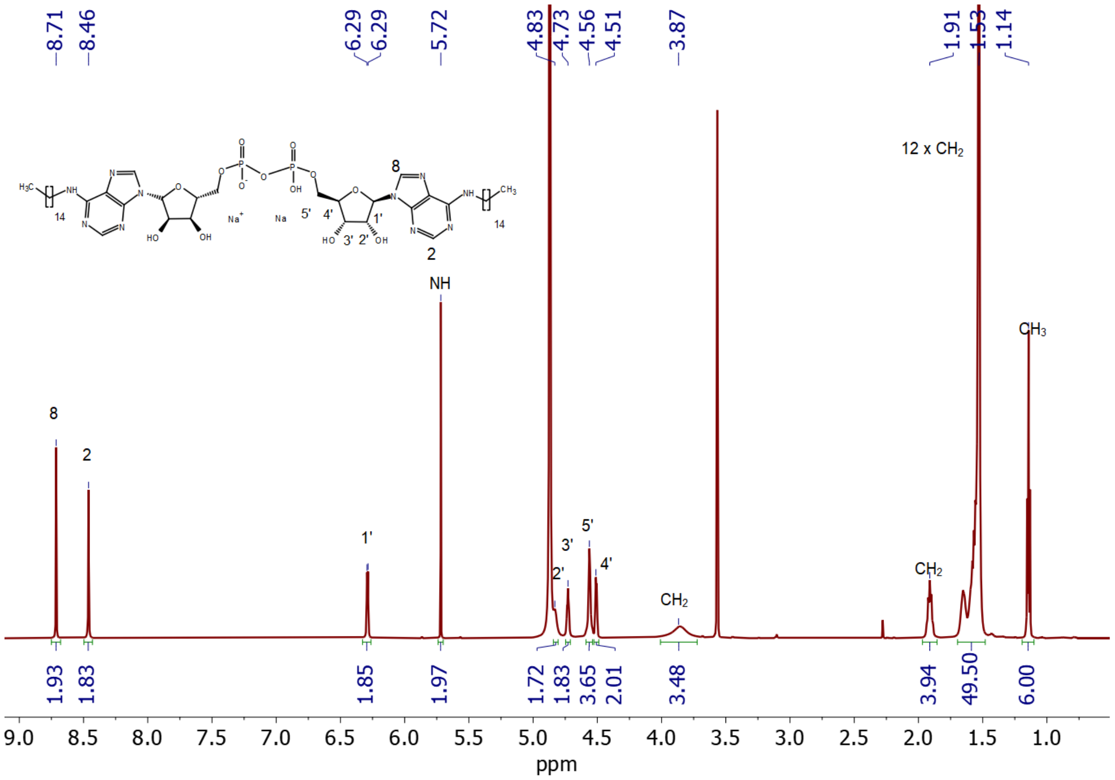

Figure S81. ^{1}H NMR spectrum of **X15ppX15** (MeOH-d4, 600 MHz, 323 K).

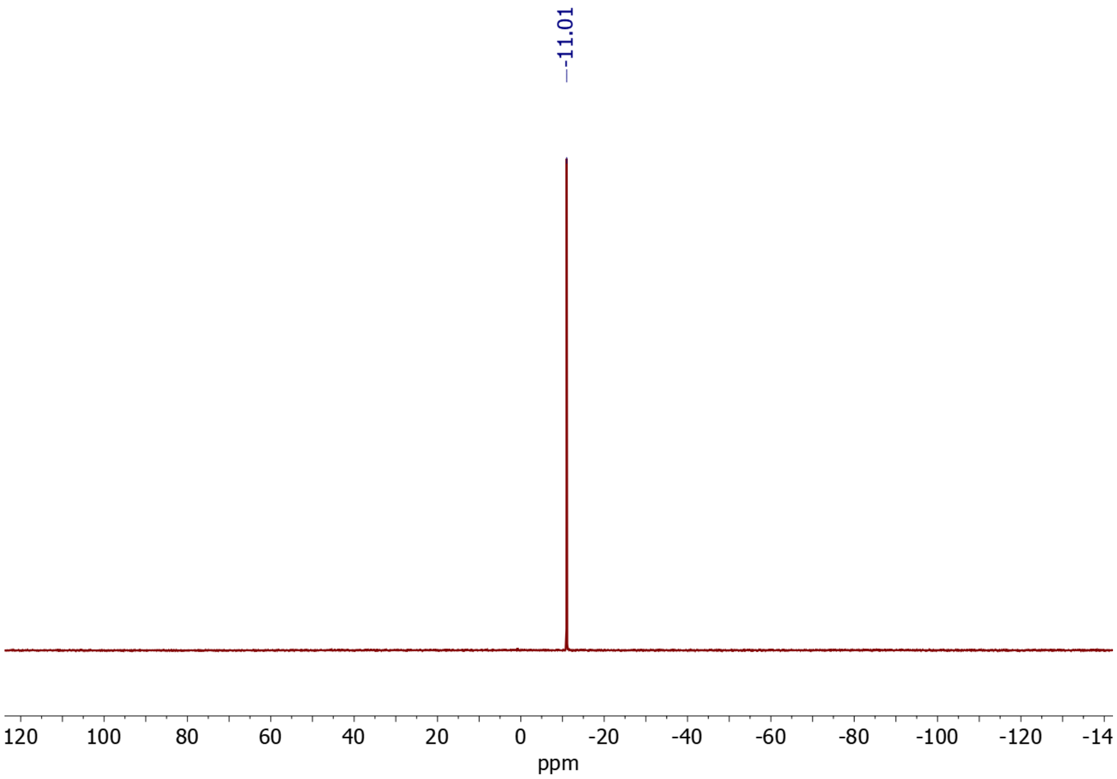

Figure 82. ^{31}P NMR spectrum of **X15ppX15** (MeOH-d4, 243 MHz, 323 K).

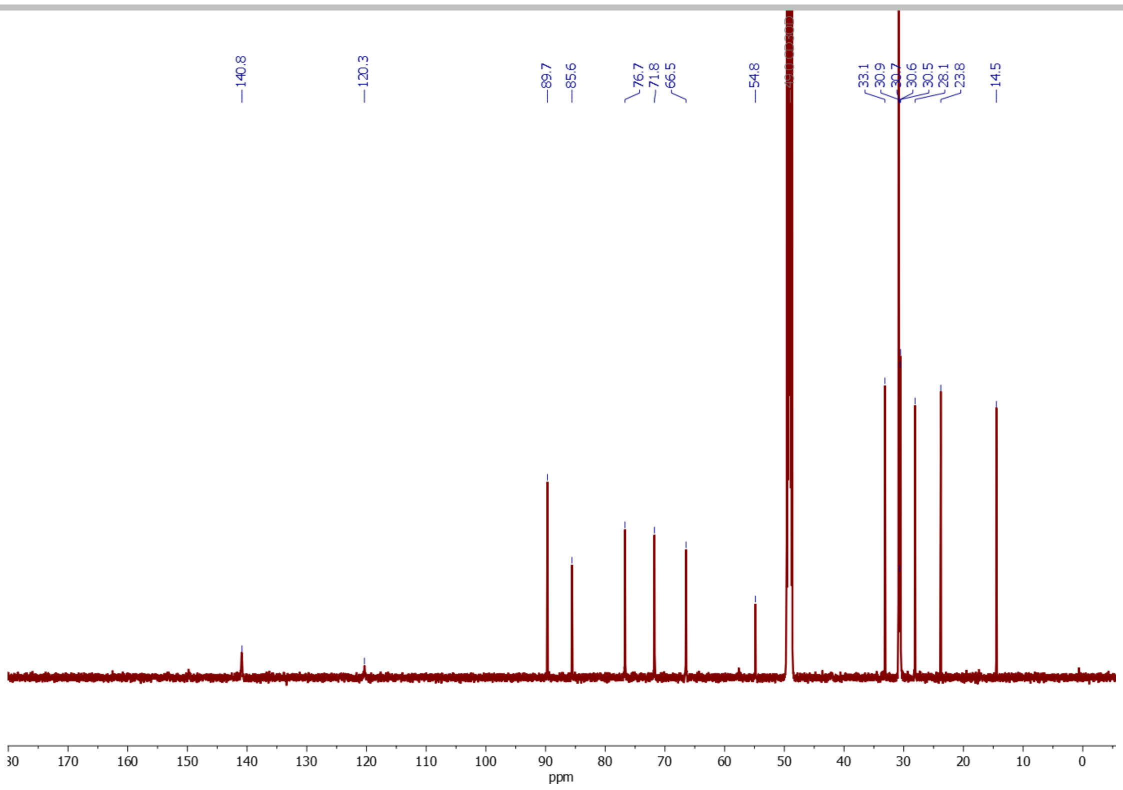

Figure 83. ^{13}C NMR spectrum of **X15ppX15** (MeOH-d_4, 150 MHz, 323 K).

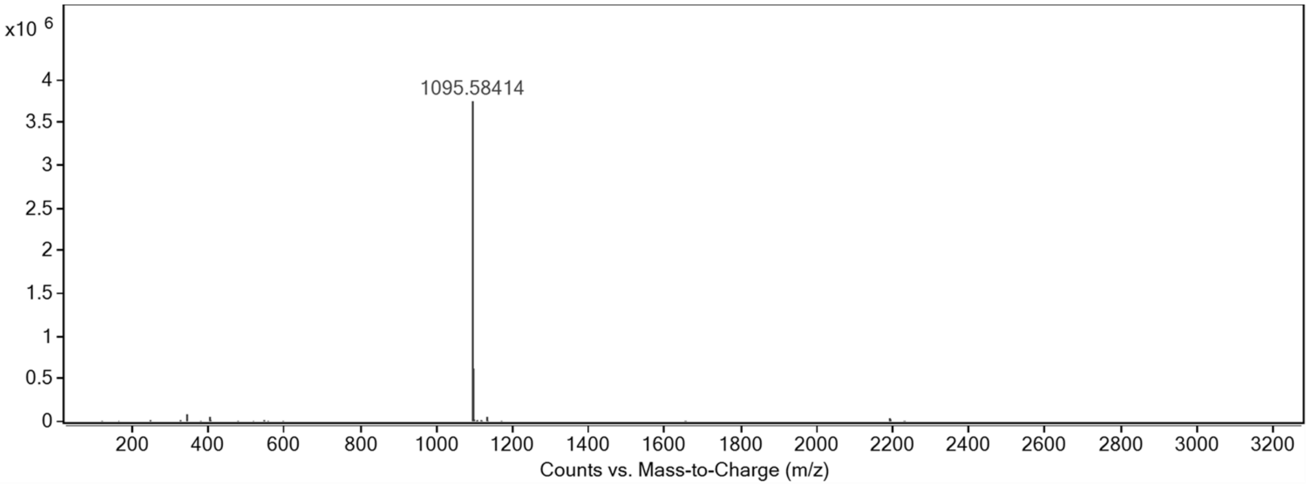

Figure 84. HR-MS (-) of **X15ppX15.**

References

[1] S. B. Salunke, N. S. Babu, C. T. Chen, *Chem. Commun.* **2011**, *47*, 10440–10442.

[2] K. El Akri, K. Bougrin, J. Balzarini, A. Faraj, R. Benhida, *Bioorg. Med. Chem. Lett.* **2007**, *17*, 6656–6659.

[3] B. L. Wilkinson, H. Long, E. Sim, A. J. Fairbanks, *Bioorg. Med. Chem. Lett.* **2008**, *18*, 6265–6267.

[4] S. Wallrodt, A. Buntz, Y. Wang, A. Zumbusch, A. Marx, *Angew. Chem. Int. Ed.* **2016**, *55*, 7660–7664.
